# E-CatBoost: An efficient machine learning framework for predicting ICU mortality using the eICU Collaborative Research Database

**DOI:** 10.1371/journal.pone.0262895

**Published:** 2022-05-05

**Authors:** Nima Safaei, Babak Safaei, Seyedhouman Seyedekrami, Mojtaba Talafidaryani, Arezoo Masoud, Shaodong Wang, Qing Li, Mahdi Moqri

**Affiliations:** 1 Department of Business Analytics and Information Systems, Tippie College of Business, University of Iowa, Iowa City, IA, United States of America; 2 Civil and Environmental Engineering Department, Michigan State University, East Lansing, MI, United States of America; 3 Department of Computer Science and Engineering, University of Nevada, Reno, NV, United States of America; 4 Department of Management, University of Tehran, Tehran, Iran; 5 Department of Industrial and Manufacturing Systems Engineering, Iowa State University, Ames, IA, United States of America; 6 Department of Information Systems and Business Analytics, Ivy College of Business, Iowa State University, Ames, IA, United States of America; Stanford University School of Medicine, UNITED STATES

## Abstract

Improving the Intensive Care Unit (ICU) management network and building cost-effective and well-managed healthcare systems are high priorities for healthcare units. Creating accurate and explainable mortality prediction models helps identify the most critical risk factors in the patients’ survival/death status and early detect the most in-need patients. This study proposes a highly accurate and efficient machine learning model for predicting ICU mortality status upon discharge using the information available during the first 24 hours of admission. The most important features in mortality prediction are identified, and the effects of changing each feature on the prediction are studied. We used supervised machine learning models and illness severity scoring systems to benchmark the mortality prediction. We also implemented a combination of SHAP, LIME, partial dependence, and individual conditional expectation plots to explain the predictions made by the best-performing model (CatBoost). We proposed E-CatBoost, an optimized and efficient patient mortality prediction model, which can accurately predict the patients’ discharge status using only ten input features. We used eICU-CRD v2.0 to train and validate the models; the dataset contains information on over 200,000 ICU admissions. The patients were divided into twelve disease groups, and models were fitted and tuned for each group. The models’ predictive performance was evaluated using the area under a receiver operating curve (AUROC). The AUROC scores were 0.86 [std:0.02] to 0.92 [std:0.02] for CatBoost and 0.83 [std:0.02] to 0.91 [std:0.03] for E-CatBoost models across the defined disease groups; if measured over the entire patient population, their AUROC scores were 7 to 18 and 2 to 12 percent higher than the baseline models, respectively. Based on SHAP explanations, we found age, heart rate, respiratory rate, blood urine nitrogen, and creatinine level as the most critical cross-disease features in mortality predictions.

## 1. Introduction

With the advent of the big data era, clinical practices have profoundly benefitted from data analytical techniques. Clinical analytics has helped in efficiently extracting and storing medical data, revealing hidden relationships and patterns, and providing invaluable insights into the diagnosis and treatment of diseases. Medical analytics has improved illness prediction accuracy, helped diagnose diseases at an early age, and enhanced the cure rate of infections [1]. The intensive care unit (ICU) is one of the primary clinical units in hospitals that has been remarkably affected by data analytics applications in recent years [2]. Patients in ICUs suffer from severe life-threatening injuries and illnesses which require intensive life-saving care and interventions. Accordingly, patients are strictly monitored in ICUs to detect deteriorating physiological changes, which continually generates enormous amounts of medical records, including vital sign measurements, care plan documentation, illness severity measures, and diagnosis and treatment information [3]. By applying various data-driven models to this big data monitoring, invaluable medical perspectives can be gained to enhance critical care services [4].

Predicting ICU mortality and estimating the length of hospitalization have tremendous significance in healthcare analytics. These analytical studies contribute significantly to enhancing patient outcomes, optimizing ICU resource utilization, and improving the financial performance of ICU management systems [5]. To accomplish these clinical analytics’ goals, several previous studies (e.g., [6, 7]) have applied the already developed models on their dataset. These studies have mainly utilized ICU scoring systems, often multivariate regression models, that merely combine predictive variables present at the time of ICU admissions to make predictions [8, 9]. The most commonly used illness severity scoring systems include Acute Physiology and Chronic Health Evaluation (e.g., APACHE IV/IVa [10]), Simplified Acute Physiology Score (e.g., SAPS III [11]), and Mortality Probability Model (e.g., MPM_0_III [12–14]). Also, several previous studies (e.g., [6, 15]) have considered a subset of patients with a specific disease type to investigate predictive factors among these patients. The main reason for such consideration is that identifying the prognostic factors of patients with particular diseases is insightful for evaluating medical intensive care and clinical decision-making [6].

Although the already proposed ICU scoring systems are acceptable standards for quantifying the ICU patients’ severity of illness and predicting their mortality status, researchers have recently argued about their limitations and weaknesses [16]. In this regard, researchers have strongly claimed that the current ICU scoring frameworks are not optimal, and they generalize sub-optimally [17]. Accordingly, an increasing interest has recently appeared in implementing machine learning (ML)-based approaches for ICU predictive analytics. However, these approaches often cannot provide explicit interpretable models [8]. "A model can be defined as interpretable if the behavior of the model can be explained verbally and that the model can be used for reasoning" [18]. Such models can describe why the framework results in a particular prediction [19], so medical staff can gain insights and derive rules over the roots of a specific risk or outcome (survival/death) [17]. Also, patients about whom algorithms make decisions can know the basis of the decisions and the factors influencing them [20]. Without any doubt, for clinical applications, model interpretability is a significant boon due to the complexity of the phenomena being analyzed and the potential repercussions of wrong decisions [18]. Most state-of-the-art ML-based mortality prediction models, which often propose highly accurate results, are black-box models and do not offer explainable frameworks. As such, these frameworks do not present actionable insights, and they might not be used within the relevant decision support systems in hospitals [19, 22]. Thus, it is essential to develop mortality prediction models that are both accurate and interpretable. Model interpretability can either be pursued by developing intrinsically interpretable models (e.g., Logistic Regression and Decision Tree) or by using interpretable surrogate models as post-hoc explanation tools (e.g., SHapley Additive exPlanations (SHAP) and Local Interpretable Model-Agnostic Explanations (LIME)) to explain the black box models.

Towards addressing this vital need, the current study proposes an accurate and explainable machine learning framework to predict the mortality of critically ill patients admitted to the ICU at the time of discharge. The authors investigate how the created models work for patients with different categories of diagnosed diseases. In summary, our study has five notable contributions to the literature, which is noteworthy considering the current needs in this area:

- We have proposed highly accurate and efficient mortality prediction models with superior performance to the widely used ICU scoring systems and state-of-the-art mortality prediction models. We have also provided extensive baseline performance evaluations and compared the results with our proposed model. Using post-hoc explanation tools, our proposed model (E-CatBoost) achieves comparable transparency to the clinical illness severity scoring systems while having similar performance to sophisticated black-box models in terms of accuracy. Additionally, our proposed model requires a significantly lower number of input features than most of the compared baselines.
- We have developed, tuned, and validated our models using the recently released eICU Collaborative Research Database v2.0, a multi-center extensive electronic database containing the data for over 200,000 ICU admissions at 208 hospitals throughout the United States.
- Most previous studies [21–24] have relied on proposing a single mortality prediction model for their entire patient population or have solely focused on a specific patient group. However, in this paper, we have meticulously divided the patient population into twelve representative groups based on their root hospitalization diseases and have proposed and tuned the mortality prediction models for each disease category. Thus, we have proposed optimal mortality prediction models for various disease types while covering almost the entire patient population.
- We have identified the most critical risk factors in increasing the patient mortality probability in ICUs across the defined disease groups. Moreover, we have analyzed how changing each underlying factor increases/decreases the mortality probability in each group. We have also revealed the critical ranges of values in each feature domain and the corresponding contribution of each feature to the patient mortality risk. Also, in addition to making inferences from group-level analyses, we have studied the important factors and their critical ranges of values associated with heightened mortality probability in individual patients. This helps medical specialists to have a clear understanding of the decision-making process of our proposed model.
- We performed feature selection on the entire set of biological, physiological, and medical variables and used the top ten most informative features for mortality prediction. In this way, we proposed E-CatBoost, a highly optimized and efficient CatBoost-based model with disease-specific features to predict patient mortality. Limiting the input information and tuning the models to make optimum predictions facilitated the mortality prediction without sacrificing accuracy.

This study is organized into five main sections. Section 2 briefly discusses the recent advancements in developing interpretable ML-based mortality prediction models. Section 3 discusses the methodology and briefly describes the data, the baseline models, and the explanation tools used in the study. Section 4 reports on the results of the analyses and provides a comprehensive description of the observations. Section 5 provides a conclusion about our findings and delineates the future goals of this research.

## 2. Related work

In the pertinent literature, previous studies have proposed interpretable machine learning models for predicting the mortality of ICU patients. Some of the recent studies related to the goals of this paper are described in this section.

Davoodi and Moradi [25] developed a Deep Rule-Based Fuzzy System (DRBFS) to propose a robust mortality prediction of ICU patients by employing fuzzy systems, deep learning, and mixed variable clustering. They used a modified supervised fuzzy k-prototype clustering to produce fuzzy rules and developed their model using the Medical Information Mart for Intensive Care (MIMIC-III) dataset. In DRBFS, the hidden layer in each unit was demonstrated by explainable fuzzy rules. The performance of the proposed method was compared with several baseline classifiers, including Naïve Bayes (NB), Decision Trees (DT), Gradient Boosting (GB), Deep Belief Networks (DBN), and Deep Takagi-Sugeno-Kang fuzzy classifier (D-TSK-FC). The results showed that DRBFS has superior performance to these classifiers despite being interpretable.

Nanayakkara et al. [22] developed accurate mortality prediction models for patients with records of cardiac arrest 24 hours before ICU admission. The models were developed based on Logistic Regression (LR) and other machine learning models, including Gradient Boosting Machine (GBM), Support Vector Classifier (SVC), Artificial Neural Network (ANN), and tree-based ensembles. They extracted the data from the Australian and New Zealand Intensive Care Society (ANZICS) Adult Patient Database. The outcomes of ML models were compared with the APACHE III and Australian and New Zealand Risk of Death (ANZROD) models. Also, explainable models were devised to recognize the most crucial physiologic attributes for a patient’s survival. The results indicated that nonlinear ML models improved mortality prediction following cardiac arrest and had better performance than LR and illness severity scoring frameworks.

Caicedo-Torres and Gutierrez [17] devised a visually interpretable deep learning framework to predict ICU mortality using Convolutional Neural Networks (CNNs) and Shapley values. They trained their deep multi-scale convolutional architecture using the MIMIC-III dataset. They designed their algorithm based on the notions from coalitional game theory. The results confirmed that the proposed model is competitive compared to the state-of-the-art deep learning mortality prediction models while remaining interpretable.

Chen et al. [26] introduced a new interpretable analysis framework that concurrently analyzes organ systems to predict the illness severity of ICU patients and their mortality risk. They developed an interpretable deep learning model (AMRNN) using Recurrent Neural Networks (RNNs) and the attention mechanism. They used a single Long-Short Term Memory (LSTM) unit to learn physiological attributes of each organ system in multivariate time series. The researchers also exploited a shared LSTM to use relationships across various learning tasks to enhance the prediction performance. They used the MIMIC-III dataset to conduct experiments and compared their results with the state-of-the-art baseline classifiers.

Huang et al. [27] developed a community-based federated machine learning (CBFL) algorithm. They assessed the performance of their method on non-identically independently distributed (non-IID) ICU electronic medical records (EMRs) to predict ICU mortality and length of stay. CBFL was built using the eICU-CRD. Their proposed method clustered the data into clinically significant groups based on diagnosis results and regional locations. The data was stored at hospitals throughout the learning process, while locally measured outcomes were put on a server. Assessment results indicated that CBFL was superior to the baseline federated machine learning (FL) algorithm.

Shickel et al. [28] proposed a novel real-time illness acuity scoring system (DeepSOFA) based on interpretable deep learning models and temporal measurements during the ICU stay. Researchers used a modified RNN with gated recurrent units (GRU). They trained their model using UFHealth and MIMIC datasets. They compared the performance of DeepSOFA with SOFA (Sequential Organ Failure Assessment) and found notably more accurate mortality predictions than the baseline model.

Deshmukh et al. [23] proposed an explainable machine learning model to predict the associated mortality with acute gastrointestinal (GI) bleeding. They used the eICU Collaborative Research Database to extract the data for patients admitted with GI bleed. They used a gradient boosting model (XGBoost) for mortality prediction and compared its predictive performance with APACHE IVa using AUROC scores. They used machine learning interpretability tools (SHAP) to explain the model’s prediction results. Their model showed superior performance in predicting the mortality of patients with GI bleed compared to the existing scoring systems.

Hu et al. [29] proposed an explainable machine learning model to predict mortality in influenza patients using the records of 336 patients at eight medical centers in Taiwan. They used XGBoost to construct the prediction model and evaluated its performance compared to Logistic Regression and Random Forest models. Moreover, the researchers measured the feature importance values and used SHAP plots to visualize the interpretation. According to the results, XGBoost outperformed the tested baseline models.

Pan et al. [24] constructed an ML-based model to analyze risk factors and predict mortality of ICU patients diagnosed with COVID-19. They used hypothesis testing, correlation, and factor analysis on 123 patients with COVID-19 to identify potential ICU risk factors. Then, they employed conventional logistic regression methods and four machine learning algorithms, including Adaptive Boosting (AdaBoost), Gradient Boosting Decision Tree (GBDT), eXtreme Gradient Boosting (XGBoost), and CatBoost to develop the risk prediction models. They used AUROC scores to measure the performance of these machine learning models. They also used calibration curves, SHAP, and LIME to explain and evaluate the risk prediction models. As a result, eight crucial risk factors were detected and included in the prediction model. Finally, they reported that the XGBoost model created using the eight important risk factors has the best performance in predicting the risk of death in ICU patients with COVID-19.

Jiang et al. [30] developed an explainable ML algorithm using the MIMIC-III database to predict mortality in sepsis survivors readmitted to the ICU within one year. The model could identify the indicative features correlated with mortality risk in the target group. Moreover, they visualized the association between the risk features and predicted mortality using Shapley values.

A methodological summary of the related works is provided in Table 1.

**Table 1:** A methodological overview of the related works.

Ref.	Algorithm(s)	A brief overview of the algorithm(s)	Limitations of the research methodology
[17]	ISeeU	A visually interpretable deep learning framework based on CNNs, Shapley values, and coalitional game theory	Using a high number of input features in developing the models; considering the same performance for the models across various disease groups; using a limited number of baselines for assessing the performance of the models
[22]	Logistic Regression; Gradient Boosting Machine; Support Vector Classifier; Artificial Neural Network; and tree-based ensembles	A set of machine learning models	Using a high number of input features in developing the models; using a limited number of baselines for assessing the performance of the models
[23]	XGBoost	A gradient boosting machine learning model	Using a high number of input features in developing the models; using a limited number of baselines for assessing the performance of the models
[24]	Logistic Regression; AdaBoost; GB trees; XGBoost; and CatBoost	A set of machine learning models	Using a limited number of baselines for assessing the performance of the models; using a small data sample for developing the models
[25]	A deep rule-based fuzzy system	A modified supervised fuzzy k-prototype clustering model	Developing explainable but not highly accurate models; using a high number of input features in developing the models; considering the same performance for the models across various disease groups; using a limited number of baselines for assessing the performance of the models
[26]	Attended multi-task recurrent neural networks	An interpretable deep learning model based on RNNs and the attention mechanism	Using a high number of input features in developing the models; considering the same performance for the models across various disease groups
[27]	A community-based federated machine learning algorithm	A method for clustering the data into clinically significant groups based on diagnosis results and regional locations	Using a limited number of baselines for assessing the performance of the models
[28]	DeepSOFA	A modified RNN with GRU units and temporal measurements	Using a high number of input features in developing the models; considering the same performance for the models across various disease groups; using a limited number of baselines for assessing the performance of the models
[29]	XGBoost	A gradient boosting machine learning model	Using a high number of input features in developing the models; using a limited number of baselines for assessing the performance of the models; using a small data sample for developing the models
[30]	LightGBM	A gradient boosting tree algorithm	Using a high number of input features in developing the models; using a limited number of baselines for assessing the performance of the models; using a small data sample for developing the models

Source: elaborated by the authors.

## 3. Materials and methods

### 3.1. Data description

We extracted the data for this study from the eICU Collaborative Research Database v2.0 (eICU-CRD v2.0). This database was created through the work of Philips eICU Research Institute (eRI) in a telehealth program to assist healthcare providers and researchers by facilitating their access to the patients’ medical records. The database includes medical information about 200,000 ICU admissions across 208 U.S. hospitals in 2014 and 2015. The original database is a collection of 31 tables, 6 of which are used in this study. We merged the selected tables using patient unit stay IDs. S1 Table provides the list and a brief description of these tables.

**Fig 1:**
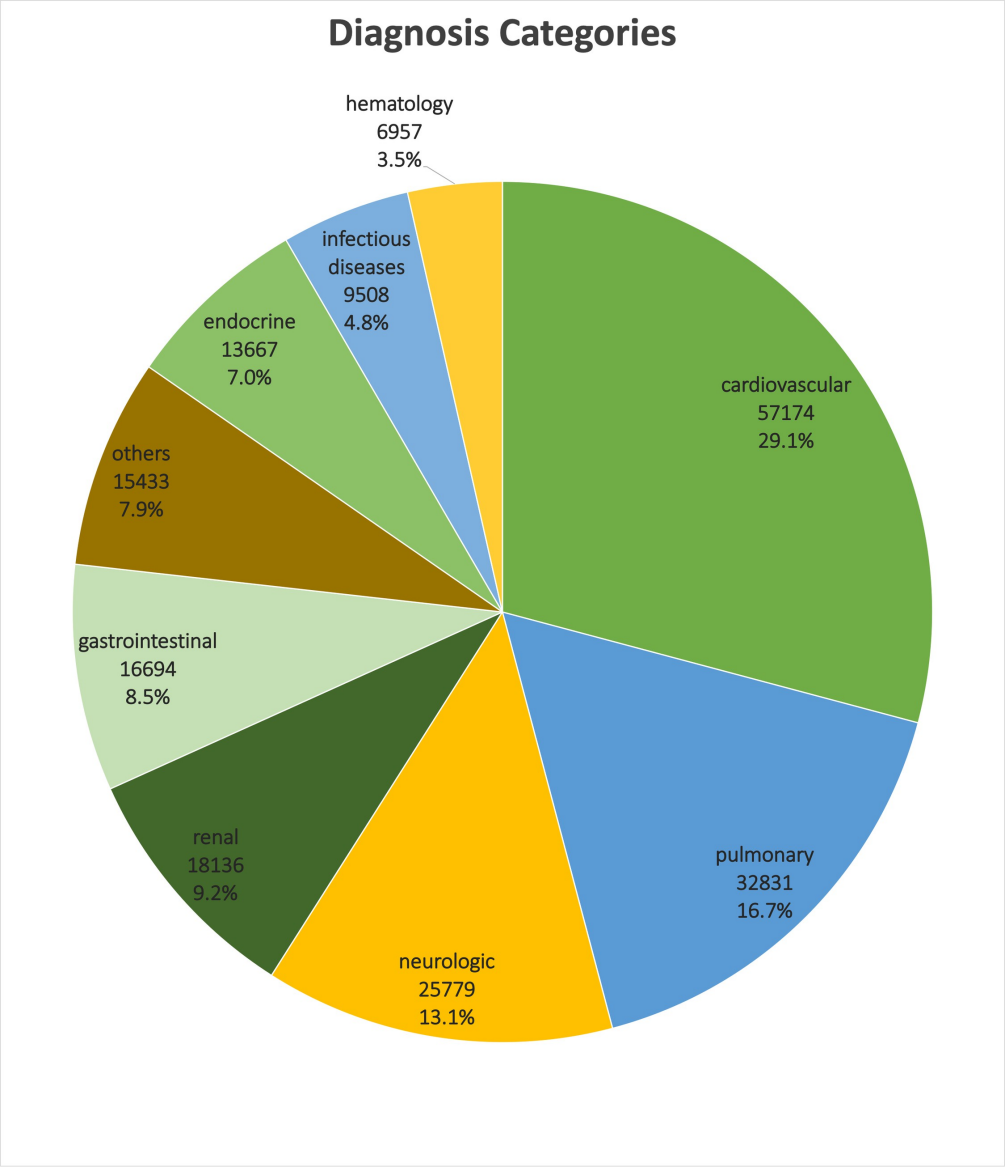
The relative frequency of twelve diagnosed disease groups in the eICU-CRD v2.0.

Notably, this study focuses on the patient data recorded before and up to 24 hours after admission to the ICUs. There are 26 categorical and 39 numerical features in this dataset. Due to the disparate pathophysiology of diseases and different medical characteristics of admitted patients based on their diagnosed conditions, we divided the dataset into twelve disease categories to analyze each group separately (Fig 1). The categories were identified by considering the patients’ diagnosis and diagnosis priority types; only the "major" and "primary" diagnosis groups were used in the analysis. In cases where multiple disease types were recorded for a patient, only the most recent diagnosis and the one with the highest priority (i.e., "primary") were considered for each diagnosis category. Thus, a patient can be categorized into multiple diagnosis groups. Disease categories with less than 500 patients were excluded from the study.

While preparing the dataset, we made significant efforts to clean and preprocess the data. In this regard, the valid and possible ranges for each column (e.g., lab test features) were extracted from medical resources and used to identify outliers and errors in data. For instance, the data points with irrational values were counted as errors. Null and misrecorded data were considered as missing information. Then, features with over 30 percent missing values were excluded from the analysis; this resulted in 29 numerical and 26 categorical features (including mortality status). To access a complete list of features used in this study, refer to S2 Table. Missing values for numerical features were imputed with the mean value in each disease group. In addition, missing values for categorical features were imputed as a separate “missing” category. Finally, the cleaned tables were merged into a master database for the subsequent analysis. The descriptive statistics summaries of the numerical and categorical features across different diagnosis groups are provided in S3–S26 Tables (For brevity purposes, the diagnosis string feature, which contains a full path string of about 3,000 diagnosed conditions in eCareManager, is not shown in the descriptive statistics tables).

### 3.2. Benchmarking mortality prediction using machine learning models and illness severity scoring systems

Twelve machine learning models and six most used illness severity scoring systems were trained and tested using 10-fold cross-validation to predict patient mortality in each defined disease group. This helped us conduct a comprehensive comparative analysis to predict patient mortality and find the best model based on accuracy. This also enabled us to benchmark the mortality prediction models and compare the performance of the proposed model with them. The baseline models were divided into non-gradient boosting baseline models, gradient boosting ensemble models, and illness severity scoring systems.

#### 3.2.1. Non-gradient boosting baseline models

The baseline models discussed in this part are typical non-gradient boosting machine learning algorithms with various complexity and interpretability levels. They provide a suitable foundation for gauging the strength of more advanced models against them. We used the Weka data mining software to train and test these baseline classifiers [31]. The baseline models used in this part are:

1. Naive Bayes (NB):
  Naive Bayes is among the simplest Bayesian network models. It applies the Bayes theorem and assumes strong independence among the features. NB quantifies uncertainty in predictions by providing posterior probability estimates [32].
2. Logistic Regression (LR):
  Logistic Regression is a discriminative classification model that calculates the posterior probability estimates without making any assumptions regarding class conditional probability. In this study, we used LogitBoost with a simple regression function as a base learner to fit the logistic model. The automatic attribute selection is performed by cross-validation and using the optimal number of LogitBoost iterations [33].
3. Support Vector Machine (SVM):
  Support Vector Machine is a linear classifier that can solve linear and nonlinear problems. SVM can be formulated as a convex quadratic optimization function subject to linear constraints. In this study, we used a one-class SVM from the Libsvm library. The kernel type and complexity parameters were optimized using cross-validation. Based on the hyperparameter tuning results, we chose the radial-based function as the kernel type for the SVM model [34].
4. Artificial Neural Network (ANN):
  Artificial Neural Networks consist of stacks of neurons that provide a hierarchy of features at multiple abstraction levels. This study used a neural network classifier with backpropagation to learn a multi-layer perceptron (neural network). The optimum architecture consisted of a one-layer ANN architecture with $\sqrt{\#\;of\;features}$ hidden nodes. Sigmoid was chosen as the activation function with optimized hyperparameters (learning rate = 0.3, momentum = 0.2). A decay mechanism was used to reduce the learning rate during each learning iteration. Using a decay mechanism helps stop the network’s diversion from the target output and improve the network’s general performance [35].
5. K-nearest Neighbor (KNN):
  K-nearest Neighbor is a model-free non-parametric instance-based learning algorithm that makes decisions based on the proximity of the test point to the training data. The KNN classifier used in this study selects the appropriate value of K based on cross-validation. The brute force search algorithm was used for the nearest neighbor search and a distance weight of $\frac{1}{distance}$ was implemented for distance weighting [36].
6. Decision Tree (DT):
  Decision Tree is a non-parametric classification algorithm that does not make any prior assumptions regarding the probability distribution of class labels and attributes; thus, it applies to a wide range of classification problems. DT is created by splitting the root node into various child nodes through a process known as recursive partitioning. A pruned C4.5 Decision Tree which is robust against noise and outliers was used in this study to predict the target class without overfitting [37].
7. AdaBoost:
  Boosting is an ensemble method that uses bootstrap samples created based on one another in a sequential manner. It has an iterative learning process that adaptively changes the distribution of hard-to-classify data points. The algorithm uses majority voting to make the final classification decision. In this study, the AdaBoost M1 method was used as an effective and efficient boosting technique to boost a nominal classifier of a pruned C4.5 decision tree [38].
8. Bagging:
  Bagging is an ensemble method that uses bootstrap samples generated independently in a parallel manner using a uniform distribution. In this study, a bagging method was used for bagging a nominal class classifier of a pruned C4.5 decision tree [39].
9. Random Forest (RF):
  Random Forest is an ensemble method that uses decorrelated unpruned decision trees to make the final classification decision. The algorithm manipulates both training instances (Bagging) and input attributes (using different features at each node). In this study, an RF with 500 trees was used to randomly select partitioning features out of 25 features available in the dataset at every node and construct an ensemble (forest) of random trees [40].

#### 3.2.2. Gradient boosting ensemble models

Gradient boosting trees (GBTs) have become popular algorithms for processing structured data. GBT algorithms differ based on the implementation of boosted tree algorithms, compatibilities, and limitations. This section describes the three most famous variations of GBTs for predicting ICU patient mortality: XGBoost, LightGBM, and CatBoost.

XGBoost stands for extreme gradient boosting. It started as a research project by Tianqi Chen in 2014 [41] and became famous in 2016. XGBoost is an ensemble of decision trees; it sequentially builds short and simple decision trees as each tree tries to improve the performance of the previous one. XGBoost implements gradient boosting machines with significant improvements; it also parallelizes each tree’s training and speeds up the process. XGBoost has been widely used to solve machine learning problems in health care. It has successfully been used to predict hypertension outcomes [42], diabetes, cardiovascular [43], and coronary heart diseases [44]. It has also recently been used for mortality prediction of patients diagnosed with COVID-19 [45], acute influenza [29], cardiopulmonary arrest [46], and Sepsis [47] in ICUs [48].

LightGBM is a tree-based gradient boosting model with fast training speed and high accuracy [49]. Recently, researchers have successfully implemented LightGBM on various medical analyses, such as driver mental state classification [50], blood glucose prediction [51], and chemical toxicity identification [52]. Like other tree-based gradient boosting methods, LightGBM makes inferences by adding up the outputs of multiple decision trees. For example, the predicted probability of patient expiration equals the summation of probabilities of multiple decision trees. The LightGBM algorithm is optimized by iteratively creating a new decision tree on the gradient of the trained trees. LightGBM adopts two exclusive techniques: Gradient-based One-Side Sampling (GOSS) and Exclusive Feature Bundling (EFB) to speed up the training process. Through GOSS, instances of the data with small gradients are excluded during the training of LightGBM. In 2017, Ke et al. [49] proved that GOSS achieves appropriate model accuracy because data instances with larger gradients are more important than those with smaller gradients. EFB bundles features that rarely take non-zero values simultaneously into a single feature without losing much information. In general, GOSS and EFB speed up the training process of LightGBM by reducing the amount of data and the number of features, respectively.

CatBoost, or categorical boosting, is a relatively new open-source machine learning tool developed by the Russian internet service company named Yandex in mid-2017 [53]. It is a robust algorithm among gradient boosted decision trees (GBDT) and has yielded beneficial results in standard ML-related problems. The CatBoost model was developed to address the downsides of the standard gradient boosting algorithms, such as target leakage and prediction shift. The CatBoost model alters the classical gradient boosting algorithm through ordered boosting and flexible handling of categorical features during the training phase; this is considered one of the boldest advancements of CatBoost compared to regular GBTs [53]. While incorporating different data sources and dealing with non-numerical or categorical features, CatBoost has proven to operate better than other gradient boosting algorithms such as XGBoost and LightGBM [53, 54]. Unlike deep learning models, CatBoost provides valuable results even with limited training data and computational power.

The easy-to-use and innovative format of CatBoost reduced the consumed time, chances of overfitting, and the burden of detailed hyper-parameter tuning [55]. Since the inception of the CatBoost model, researchers have used it in a diverse set of medical studies, the results of which have demonstrated the exemplary performance of this model. In a study performed by Postnikov et al. [56], the CatBoost algorithm was applied and trained on clinical data to predict drug resistance in patients diagnosed with pulmonary tuberculosis. The study results showed the algorithm’s high utility when dealing with highly scattered medical data. CatBoost is an excellent choice for clinical data since categorical features are prevalent in these datasets [57]. Shuwen et al. [58] used six algorithms, including Logistic Regression, Random Forest, SVM, GBDT, ANNs, and CatBoost, to detect liver metastasis in the early stages of colorectal cancer (CRC). Their analysis demonstrated the optimal performance of the CatBoost model for the early diagnosis of patients with liver metastasis. These findings are in line with other medical studies that have observed the satisfying performance of the CatBoost model while operating on clinical data [59, 60]. Since the eICU-CRD dataset has many non-numerical features, the CatBoost model is suitable for predicting mortality probabilities using this dataset.

#### 3.2.3. Illness severity scoring systems used in ICUs

Since 1980, numerous illness severity scoring systems have been tailored for the ICUs to predict survival/mortality risks, determine the severity of diseases and degree of organ dysfunction, optimize resource use and enhance patient outcomes. The proposed illness severity scoring systems that are frequently used to evaluate the conditions of critically ill patients are categorized into two major groups: 1- Methods that predict mortality at the time of ICU admissions, such as Acute Physiology and Chronic Health Evaluation (APACHE), Simplified Acute Physiology Score (SAPS), and Oxford Acute Severity of Illness Score (OASIS). 2- Methods that evaluate organ dysfunction degrees in admitted patients, such as Multiple Organ Dysfunction Score (MODS), Sequential Organ Failure Assessment (SOFA), and Logistic Organ Dysfunction System (LODS). This section briefly discusses these systems.

1. Acute Physiology and Chronic Health Evaluation (APACHE): The original APACHE was developed in 1981 to assess the illness degree and categorize patients based on their illness severity [61]. So far, the statistical methodology and the physiological variables APACHE uses have been revised multiple times. This has resulted in the release of APACHE IV/IVa as the most recent version of APACHE and one of the world’s most widely used ICU scoring systems. APACHE IV was developed using the information on 100,000 patients from 104 ICUs in 45 US hospitals. It uses 142 patient variables, and for each physiological variable, it uses the worst value during 24 hours of admission [10].
2. Simplified Acute Physiology Score (SAPS): The original SAPS was developed in 1984 and used age and thirteen other physiological variables to predict patient mortality [62]. SAPS II is a modified version of SAPS that uses a logistic regression analysis using 17 patient variables (twelve physiological variables, three disease variables, age, and admission). SAPS II was developed using patient information from 137 ICUs in 12 countries [63]. A more recent version, SAPS III, was created using a sophisticated statistical technique with the data gathered from 303 ICUs over 35 countries [11].
3. Oxford Acute Severity of Illness Score (OASIS): With an attempt to simplify the mortality predictions of illness severity scoring indices, OASIS was developed in 2013, which only requires ten variables routinely collected in ICUs (e.g., no laboratory test results required) to accelerate the mortality prediction [64].
4. Multiple Organ Dysfunction Score (MODS): Using the results of more than 30 published reports and a comprehensive literature review on organ dysfunction between 1969 and 1993, MODS was proposed to measure the severity of multiple organ failure syndromes including five organs (respiratory, renal, hepatic, hematologic, and central nervous systems) [65]. Due to the high correlation between the dysfunction of multiple organs with mortality probability, MODS is widely used for illness severity scoring and ICU mortality prediction.
5. Sequential Organ Failure Assessment (SOFA): The European Society of Intensive Care and Emergency Medicine developed SOFA at a consensus conference in 1994. Like MODS, SOFA measures the severity of six failed organs (cardiovascular, hepatic, renal, respiratory, coagulation, and central nervous system), which is highly predictive of the patients’ discharge status [66].
6. Logistic Organ Dysfunction System (LODS): Using the physiological data of 13,520 admission records from 137 adult patients across 12 countries, LODS assesses the severity level of six organ failures by conducting a logistic regression analysis. The system considers renal, neurologic, and cardiovascular failures as the most severe organ failure types and pulmonary, hematologic, and hepatic failures as the least severe organ failures. In this way, LODS considers the relative severity of organ failure in addition to the failure severity degrees in an individual organ to calculate the illness severity score and mortality probability [67].

The illness severity scoring systems proposed in this section are used as baselines to compare the performance of the proposed mortality prediction model with them. Although most of these clinical scoring systems have simple methodologies, they are still widely used in ICUs to evaluate patients’ illness severity and survival chances due to their high degrees of explainability to physicians and medical experts. Proposing a model that can effectively learn complex relationships between patient features using a significantly smaller amount of input information is a promising objective this paper pursues.

### 3.3. Explaining the ICU mortality risk factors using SHAP and LIME

In recent years, there has been massive interest in applying interpretation tools on tree-based ensemble models (such as Random Forest and Gradient Boosting Trees) used for ICU mortality prediction [68, 69]. Ensemble tree-based models generally result in more accurate predictions than simpler models like Logistic Regression. However, the black-box nature of these models does not disclose the decisive factors in determining the patient’s discharge status (survival or expiration), whether these factors are protective or dangerous, and what ranges of values in each factor affect the mortality probability the most. Thus, these models are not in favor of clinicians. Consequently, in this research, we applied SHapley Additive exPlanations (SHAP) and Local Interpretable Model-Agnostic Explanations (LIME) as surrogate explanation tools to our best-performing mortality prediction model to improve the model’s explainability. Using these post-hoc explanation tools enabled our model to maintain its high accuracy while providing decent explanations about the model. A detailed description of the results is provided in the results and discussion section (Section 4).

Shapley values are based on cooperative game theory and were introduced by Shapley in 1953. These values demonstrate the contribution of each feature in the outcomes of both classification and regression models. While calculating the Shapley values, all permutations of feature values are evaluated to calculate the average marginal impact of a feature value on prediction results. This process is repeated for all features to calculate their Shapley values. Features with higher Shapley values are identified as more important features because they make a higher contribution to the model’s prediction results. There are numerous methods in the literature for interpreting model predictions and estimating Shapley values; however, finding the most efficient method for various scenarios has been open to scientific debates. In 2016, Lundberg and Lee introduced SHAP (Shapley Additive exPlanations) values as a unified “measure for feature importance” and proposed several efficient and consistent methods for estimating them. SHAP values “are the Shapley values of a conditional expectation function of the original model” [70]. This paper uses TreeSHAP [71], a fast estimation technique suitable to compute Shapley values in tree-based models such as Gradient Boosting Trees and Random Forests.

Local Interpretable Model-Agnostic Explanations (LIME) is a general framework that can locally interpret any black-box machine learning model. LIME has been widely used in various medical applications, such as explaining machine learning models to predict hypertension [72], mortality risk following cardiac arrest [22], and mortality of influenza patients in ICUs [29]. LIME explains which variables make the prediction decision and reveals the contribution of each feature for local instances. LIME is implemented in five steps: 1- Based on a particular instance that we will explain later in this paper, LIME generates similar samples by tweaking some feature values. 2- It predicts class labels of the new samples with the trained black-box model. 3- It weighs the new samples and their class labels by proximity to the target instance. 4- It trains an interpretable model, such as Logistic Regression, on the weighted samples and labels. 5- It explains the feature importance using the interpretable model.

### 3.4. Partial dependence (PD) and individual conditional expectation (ICE) plots

Partial dependence (PD) and individual conditional expectation (ICE) plots are famous for gaining insight into the black box models. We use these plots to study the mortality prediction’s sensitivity to the monotonic changes in the underlying feature values. PD plots map the marginal effect of the selected variables to uncover the linear, nonlinear, or monotonic relationship between the response variable and the individual feature variables [73]. PD accounts for the nonlinearity of the data, and by averaging out the effect of other features, represents the global average prediction of the monotonic model as a function of different values of a specific feature. PD plots improve the transparency of the black-box model and enable debugging and validation across the feature domains. On the other hand, ICE plots are used to disaggregate the average prediction of monotonic models; they perform nonlinear sensitivity analysis and show the changes in the model predictions for an individual instance as a result of modifying a specific feature value in its domain. For each instance (patient), ICE plots represent the functional relationship between an individual feature and the predicted target value. In other words, PD plots average the individual lines of ICE plots [73].

## 4. Results and discussion

In this section, first, we present cross-validation results of individual mortality prediction models for the twelve overrepresented disease groups and also for the entire population of admitted patients; in doing so, we focus on answering the following question: how does the performance of the best model compare to the performance of other baseline models (NB, LR, SVM, ANN, etc.), including ensembles (AdaBoost, Bagging, RF), gradient boosting trees (LightGBM, XGBoost, etc.), and the illness severity scoring systems (APACHE IVa, SOFA, SAPS II, etc.). First, we evaluated the performance of the mortality prediction models through 10-fold cross-validation using AUROC as the validation metric. We conducted hyperparameter tuning by holding out 10 percent of the data as the validation set in each cross-validation fold. Next, a group of explanation tools (SHAP, LIME, etc.) was applied to the best-performing mortality prediction model to identify the most critical risk factors affecting the mortality probability of patients in various disease groups. Additionally, a more in-depth analysis of the feature importance was provided using force plots to study the most critical ranges of feature values for mortality prediction. Furthermore, for each of the important features identified in the previous steps, the partial dependence (PD) and individual conditional expectation (ICE) plots were drawn to show how the monotonic increase of these features affects mortality probability across the entire patient groups. Finally, the efficiency of the best-performing model was improved by conducting feature selection and limiting the number of input features to ten in each of the overrepresented disease groups. This has resulted in the proposal of a highly optimized and efficient framework to accelerate mortality prediction without sacrificing much prediction accuracy.

### 4.1. Prediction of ICU discharge status

Table 2 shows the mean AUROCs using 10-fold cross-validation using the non-GBT baseline model settings defined in the methodology section. The feature values are normalized only during the training and testing of Logistic Regression, KNN, and ANN models. The original feature scales are maintained during training and testing for the rest of the models in the paper.

**Table 2:** Cross-validation results (AUROC) of the non-GBT baseline models using 10-fold cross-validation.

Method Disease	NB	Logistic Regression	SVM	ANN	KNN	AdaBoost	Bagging	Random Forest	Decision Tree
**burns-trauma**	0.88	0.89	0.81	0.89	0.76	0.84	0.88	0.90	0.66
**cardiovascular**	0.87	0.88	0.79	0.88	0.79	0.84	0.87	0.89	0.74
**endocrine**	0.88	0.90	0.80	0.86	0.80	0.82	0.86	0.89	0.70
**gastrointestinal**	0.85	0.86	0.77	0.72	0.76	0.85	0.85	0.88	0.75
**hematology**	0.85	0.86	0.76	0.74	0.76	0.79	0.75	0.88	0.70
**infectious diseases**	0.81	0.83	0.73	0.71	0.70	0.76	0.81	0.83	0.69
**neurologic**	0.87	0.88	0.79	0.78	0.80	0.86	0.83	0.90	0.70
**oncology**	0.82	0.83	0.73	0.81	0.71	0.79	0.86	0.89	0.70
**pulmonary**	0.80	0.83	0.74	0.79	0.69	0.77	0.80	0.84	0.69
**renal**	0.84	0.86	0.75	0.79	0.73	0.79	0.82	0.86	0.70
**surgery**	0.87	0.76	0.77	0.76	0.81	0.81	0.80	0.88	0.65
**toxicology**	0.91	0.90	0.79	0.80	0.84	0.79	0.78	0.90	0.53

Based on the results shown in Table 2, Random Forest outperforms all other non-GBT baseline models in predicting ICU patient mortality.

Using the GBT models described in the methodology section, we evaluated the performance of XGBoost, LightGBM, and CatBoost via AUROC scores. The results are summarized in Table 3.

**Table 3:** Cross-validation results (mean AUROC [standard deviation AUROC]) of GBT models using 10-fold cross-validation.

Disease group	Model	AUROC	Disease group	Model	AUROC
**burns-trauma**	LightGBM	0.91 [0.03]	**neurologic**	LightGBM	0.90 [0.04]
	XGBoost	0.93 [0.02]		XGBoost	0.90 [0.03]
	CatBoost	0.92 [0.02]		CatBoost	0.91 [0.01]
**cardiovascular**	LightGBM	0.90 [0.04]	**oncology**	LightGBM	0.86 [0.04]
	XGBoost	0.90 [0.03]		XGBoost	0.87 [0.02]
	CatBoost	0.91 [0.01]		CatBoost	0.87 [0.03]
**endocrine**	LightGBM	0.89 [0.03]	**pulmonary**	LightGBM	0.85 [0.03]
	XGBoost	0.91 [0.01]		XGBoost	0.84 [0.04]
	CatBoost	0.90 [0.01]		CatBoost	0.86 [0.02]
**gastrointestinal**	LightGBM	0.89 [0.05]	**renal**	LightGBM	0.87 [0.02]
	XGBoost	0.89 [0.02]		XGBoost	0.88 [0.03]
	CatBoost	0.89 [0.01]		CatBoost	0.88 [0.01]
**hematology**	LightGBM	0.88 [0.04]	**surgery**	LightGBM	0.88 [0.05]
	XGBoost	0.88 [0.03]		XGBoost	0.86 [0.03]
	CatBoost	0.89 [0.02]		CatBoost	0.89 [0.04]
**infectious diseases**	LightGBM	0.84 [0.05]	**toxicology**	LightGBM	0.94 [0.09]
	XGBoost	0.85 [0.02]		XGBoost	0.92 [0.08]
	CatBoost	0.86 [0.02]		CatBoost	0.92 [0.07]

The three introduced GBT models differ in main characteristics, including the splitting technique, leaf growth, and categorical feature handling. For splitting, XGBoost does not use any weighted sampling techniques; on the other hand, LightGBM uses GOSS that performs splitting using all the points with huge gradients and a random sample of points with small gradients. GOSS reduces the number of instances and balances accuracy and speed. CatBoost implements a novel splitting technique called Minimal Variance Sampling (MVS). This sampling mechanism occurs at the tree level, maximizing split scoring accuracy. XGBoost backwardly prunes the tree for leaf growth and cuts it down with no additional positive gain. Like XGBoost, LightGBM performs leaf-wise tree growth; this grows the leaf with the minimum loss and leads to an unbalanced tree. CatBoost, on the other hand, grows a balanced tree; at each tree level, the splitting pair is chosen to result in the minimum loss and then is used in all nodes of the layer. XGBoost has an internal mechanism for handling categorical features, and the operator should perform feature encoding. LightGBM divides categorical features into two subsets. The categories are sorted according to the training objective at each partition. This technique has not proven to be more effective than one-hot-encoding. CatBoost is more sophisticated in handling categorical features. It combines one-hot-encoding with advanced mean encoding and has an auxiliary process for mitigating the overfitting problem. Although the implemented encoding method has proven to improve the model performance, it slows down the process.

Based on the validation results (AUROC scores) shown in Tables 2 and 3, CatBoost outperforms the other ML-based non-GBT baselines and performs similar to or better than XGBoost and LightGBM in most of the disease groups. It is also vital to observe and compare the performance of the CatBoost model to the most widely used ICU scoring systems; Table 4 compares the cross-validation results of CatBoost with APACHE IVa, SAPS II, OASIS, MODS, SOFA, and LODS.

**Table 4:** Cross-validation results (AUROC) of CatBoost and ICU illness severity scoring systems using 10-fold cross-validation.

Disease group	Model	AUROC	Disease group	Model	AUROC
**burns-trauma**	APACHE IVa	0.88 [0.04]	**neurologic**	APACHE IVa	0.88 [0.02]
	SAPS II	0.87 [0.05]		SAPS II	0.83 [0.02]
	OASIS	0.87 [0.04]		OASIS	0.83 [0.03]
	MODS	0.80 [0.08]		MODS	0.77 [0.03]
	SOFA	0.84 [0.07]		SOFA	0.81 [0.02]
	LODS	0.80 [0.09]		LODS	0.78 [0.03]
	**CatBoost**	**0.92 [0.02]**		**CatBoost**	**0.91 [0.01]**
**cardiovascular**	APACHE IVa	0.87 [0.03]	**oncology**	APACHE IVa	0.83 [0.02]
	SAPS II	0.83 [0.06]		SAPS II	0.77 [0.03]
	OASIS	0.82 [0.06]		OASIS	0.78 [0.03]
	MODS	0.78 [0.10]		MODS	0.75 [0.04]
	SOFA	0.80 [0.08]		SOFA	0.77 [0.04]
	LODS	0.80 [0.07]		LODS	0.76 [0.05]
	**CatBoost**	**0.91 [0.01]**		**CatBoost**	**0.87 [0.03]**
**endocrine**	APACHE IVa	0.88 [0.02]	**pulmonary**	APACHE IVa	0.88 [0.02]
	SAPS II	0.84 [0.03]		SAPS II	0.78 [0.03]
	OASIS	0.84 [0.03]		OASIS	0.75 [0.02]
	MODS	0.80 [0.04]		MODS	0.73 [0.03]
	SOFA	0.82 [0.04]		SOFA	0.74 [0.03]
	LODS	0.80 [0.05]		LODS	0.75 [0.04]
	**CatBoost**	**0.90 [0.01]**		**CatBoost**	**0.86 [0.02]**
**gastrointestinal**	APACHE IVa	0.85 [0.02]	**renal**	APACHE IVa	0.83 [0.01]
	SAPS II	0.82 [0.03]		SAPS II	0.80 [0.02]
	OASIS	0.80 [0.03]		OASIS	0.79 [0.01]
	MODS	0.78 [0.04]		MODS	0.75 [0.02]
	SOFA	0.79 [0.04]		SOFA	0.77 [0.03]
	LODS	0.79 [0.05]		LODS	0.77 [0.03]
	**CatBoost**	**0.89 [0.01]**		**CatBoost**	**0.88 [0.01]**
**hematology**	APACHE IVa	0.86 [0.03]	**surgery**	APACHE IVa	0.88 [0.05]
	SAPS II	0.81 [0.04]		SAPS II	0.82 [0.05]
	OASIS	0.80 [0.05]		OASIS	0.82 [0.06]
	MODS	0.78 [0.05]		MODS	0.76 [0.06]
	SOFA	0.80 [0.04]		SOFA	0.77 [0.05]
	LODS	0.77 [0.06]		LODS	0.74 [0.06]
	**CatBoost**	**0.89 [0.02]**		**CatBoost**	**0.89 [0.04]**
**infectious diseases**	APACHE IVa	0.82 [0.02]	**toxicology**	APACHE IVa	0.92 [0.06]
	SAPS II	0.79 [0.04]		SAPS II	0.86 [0.08]
	OASIS	0.78 [0.03]		OASIS	0.87 [0.08]
	MODS	0.74 [0.03]		MODS	0.82 [0.09]
	SOFA	0.76 [0.04]		SOFA	0.85 [0.09]
	LODS	0.75 [0.05]		LODS	0.78 [0.10]
	**CatBoost**	**0.86 [0.02]**		**CatBoost**	**0.92 [0.07]**

Table 4 shows the superior performance of CatBoost compared to some popular illness severity scoring systems across the studied disease groups. APACHE IVa, one of the most widely used illness severity scoring systems in ICUs, has the best performance among other illness severity scoring systems. The AUROC score of CatBoost is up to 6 percent higher than the AUROC of the most accurate illness severity scoring system (APACHE IVa) in almost all the disease groups (except Pulmonary). If evaluated aggregately across the population of entire patients regardless of their diagnosed diseases, the cross-validation result (mean AUROC) of the CatBoost model (0.91 [std:0.004]) is 7 to 18 percent higher than the tested illness severity scoring systems (APACHE IVa = 0.85 [std:0.006], LODS = 0.78 [std:0.008], MODS = 0.77 [std:0.007], OASIS = 0.81 [std:0.006], SAPS II = 0.82 [std:0.006], SOFA = 0.79 [std:0.005]). Clinical illness scoring models can mostly be viewed as logistic regression analysis; thus, we can improve their predictive performance by replacing their simple additive nature with more complex supervised learning models such as CatBoost.

It is important to compare the performance of the CatBoost model with some state-of-the-art ML-based models proposed for mortality prediction. Due to their complex structures, these methods are generally more accurate than the ICU scoring systems. However, this increase in performance is often accompanied by a loss of transparency and explainability to the medical experts, which hampers their implementation in a comprehensive, practical setting in ICUs. The cross-validation result (mean AUROC) of the CatBoost model across the entire patient population (0.91) is higher than or almost equal to the reported validation scores for some state-of-the-art ML-based models developed and/or validated using the eICU-CRD v2.0 database (BiLSTM [74] = 0.84, community-based federated learning (CBFL) [27] = 0.70–0.75, Discharge Readiness Score (DRS) [75] = 0.86–0.94, and two-level attention-based LSTM [76] = 0.89).

In the following subsection, we implement multiple post-hoc explanation tools, including SHAP, LIME, partial dependence (PD), and individual conditional expectation (ICE) plots into the best-performing model (CatBoost) to improve its transparency and interpretability. The results help us perform feature selection and propose an alternative CatBoost-based model to perform mortality prediction using the most important features.

### 4.2. Identifying the most critical features using SHAP

**Fig 2:**
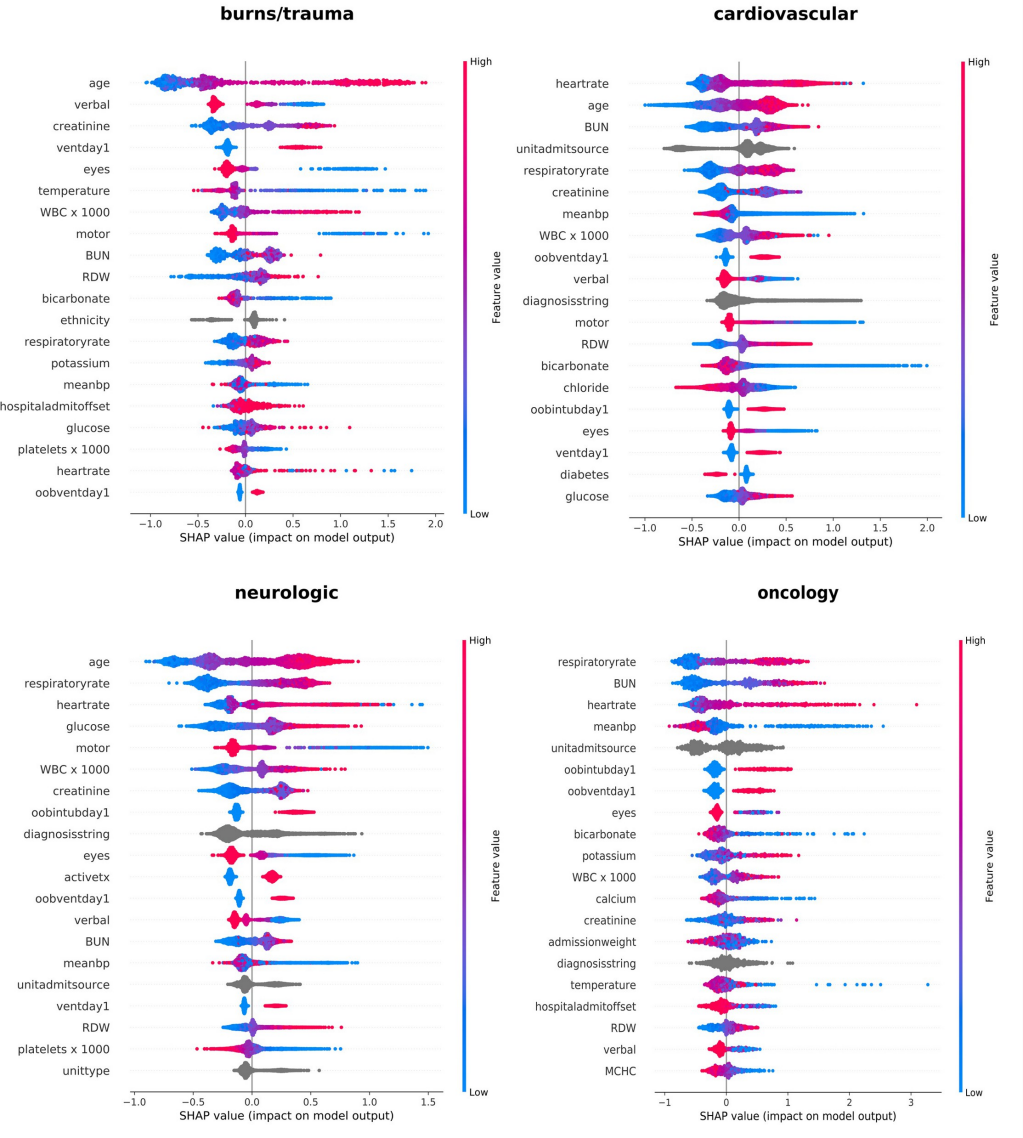
Feature importance plots based on Shapley values for burns/trauma, cardiovascular, neurologic, and oncology disease groups.

Fig 2 shows the feature importance graph for four of the twelve disease categories based on the calculated Shapley values. Graphs for the rest of the disease groups are provided in S1 and S2 Figs. Each row in these plots combines a series of points showing the Shapley values. The y-axis demonstrates features in an ordered format, with their importance increasing from bottom to top. In other words, features located higher on the y-axis contribute more to the prediction results. For instance, for patients in the cardiovascular category, heart rate, age, and BUN (blood urea nitrogen) have the highest contribution to the model prediction outcomes. The x-axis, on the other hand, is sorted according to Shapley values, increasing from origin to left or right. The color bar ranging from blue to red refers to the feature values. Since our prediction target is discharge status, larger Shapley values relate to higher mortality probability. For example, according to Fig 2, older patients in the burns/trauma category have a higher mortality risk than younger patients. Similar conclusions can be derived for patients in other disease categories (S1 and S2 Figs).

Table 5 shows the top three most important features for mortality prediction in each disease category.

**Table 5:** Top three most important features in mortality predictions across various disease categories based on Shapley values.

Disease group	Three most important features
endocrine	diabetes, age, respiratoryrate
gastrointestinal	meanbp, respiratoryrate, creatinine
hematology	platelets × 1000, heartrate, creatinine
infectious diseases	age, oobventday1, heartrate
neurologic	age, respiratoryrate, heartrate
oncology	respiratoryrate, BUN, heartrate
pulmonary	age, heartrate, BUN
renal	heartrate, age, WBC × 1000
surgery	age, admissionweight, verbal
toxicology	glucose, hospitaladmitoffset, age
burns-trauma	age, verbal, creatinine
cardiovascular	heartrate, age, BUN

The most important features (key risk factors) identified in this study (Table 5) are in line with the clinical knowledge in the area. Age is the most important factor based on having the highest frequency among the features in Table 5. It requires no stretch of credulity to imagine that age is a significant factor in ICU mortality, especially for patients older than 75 [77]. Five out of twelve disease groups have "age" as the most important feature in predicting the discharge status. Nine out of twelve disease groups have "age" among the top three most important features. Based on Table 5, heart rate was the second most important factor. Seven out of twelve disease groups have heart rate among the top three most important features in predicting mortality. On the first day of ICU admission, elevated heart rate value has a verified association with increased mortality probability [78, 79]. Respiratory rate was the third most important factor. A respiratory rate between 12 and 25 per minute is considered a normal range for this variable, and abnormal values outside this range have been verified as a critical factor in increasing mortality probability [80, 81]. Blood urea nitrogen (BUN) was the fourth most important factor. BUN is not included in most clinical ICU severity scoring systems like APACHE and SOFA. However, high BUN levels are associated with renal failure [82], congestive heart failure [83], bioenergetic muscle failure, and chronic multi-organ failure [84], which lead to an increased patient mortality risk [85]. Our study verified the importance of BUN in the mortality of the patients in the cardiovascular, pulmonary, and oncology disease groups.

**Fig 3:**
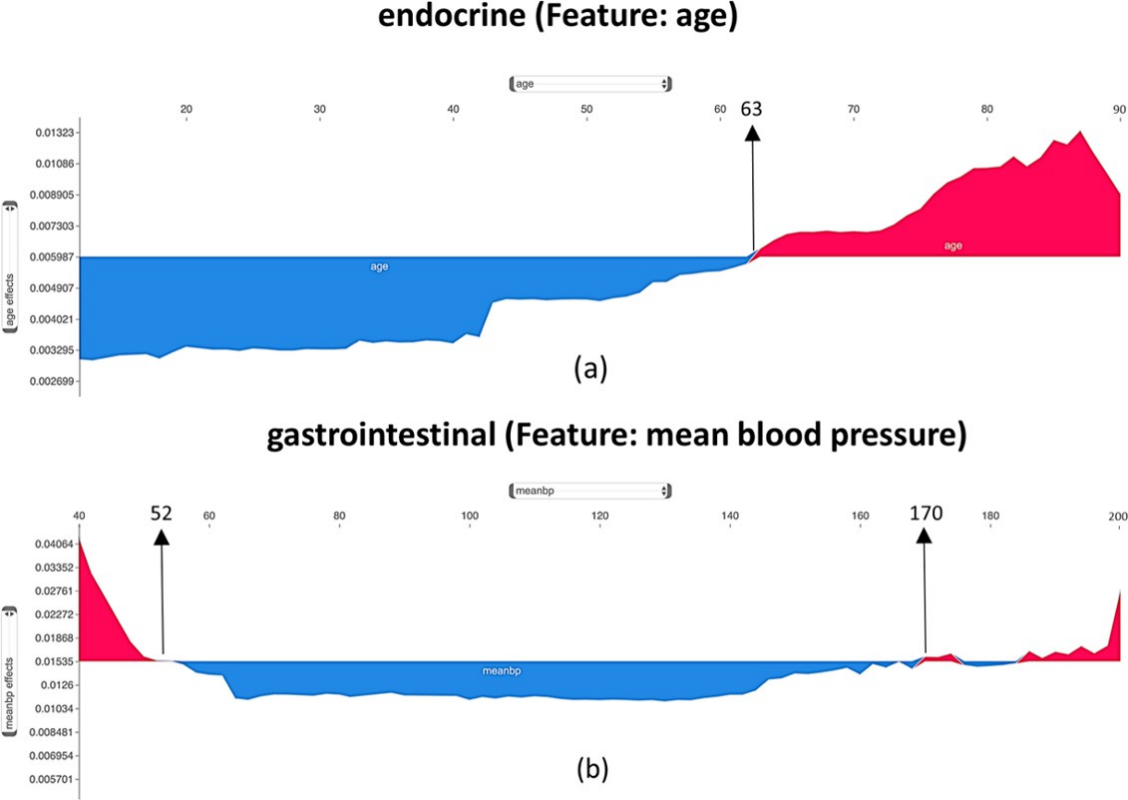
Force plots of the most important features in mortality prediction of patients in endocrine and gastrointestinal disease groups.

Furthermore, to explain the feature importance in a more detailed format, we have created a series of plots (commonly known as force plots). Two force plots for endocrine and gastrointestinal disease groups are shown in Fig 3; force plots for rest of the disease groups are shown in S3–S7 Figs.

Fig 3 explains the contribution of the most important numerical feature to model predictions in endocrine and gastrointestinal disease groups. The y-axis shows the model output values, and the x-axis refers to the values of the selected feature. The origin of the plot on the y-axis shows the base value for the model results. The base value is the mean of the predicted mortality probability for all patients in each category. Parts of these plots with blue color show the range of feature values that negatively affect the mortality risk (i.e., lower chances of death). Similarly, parts of the plot colored in red are for values that positively influence the mortality risk. S3–S7 Figs, parts k, a, d, e, g, and i refer to disease categories having age as their most important feature. According to these plots, the negative and positive impacts of age values balance each other at 63 to 68 years old. While referring to the burns-trauma category as an example, it can be understood that the age value in patients older than 68 has a positive contribution to the mortality probability. For patients younger than 68, age negatively contributes to the mortality probability.

Heart rate was identified as the most important feature in the mortality prediction of patients in the cardiovascular disease category (Fig 2). Patients with heart rates between 32 and 110 per minute have a lower mortality risk than patients with heart rate values outside this range (S7 Fig part (l)). Also, if a patient’s heart rate is in the extreme ranges (lower than 32 or higher than 110 per minute), it will increase the patient’s mortality risk (S7 Fig part (l)). Previous research found heart rate values higher than 100 per minute during the first day of ICU admission associated with an increased mortality rate [86].

Based on Fig 3 part (b), blood pressure improves the chance of survival if it is approximately between 52 and 170 for patients in the gastrointestinal disease group. According to S3 Fig part (c), platelets count higher than 132,500 in the hematology group decreases the patient mortality risk. Based on S4 Fig part (f), the effects of respiratory rate, as the most important feature in the mortality prediction of patients in the oncology disease group, are balanced on the value of 29. This feature increases the mortality risk for patients with a respiratory rate higher than 29 per minute. S5 Fig part (h) shows the effects of different heart rate ranges on the mortality prediction of the patients in the renal disease group. According to this plot, a heart rate value between 35 and 117 per minute reduces the mortality risk of patients in the renal group. Finally, based on S6 Fig part (j), a blood glucose level higher than 110 has a positive association with the mortality probability of the patients in the toxicology group.

### 4.3. Detailed feature importance explanation for individual patients in each disease category using SHAP and LIME

**Fig 4:**
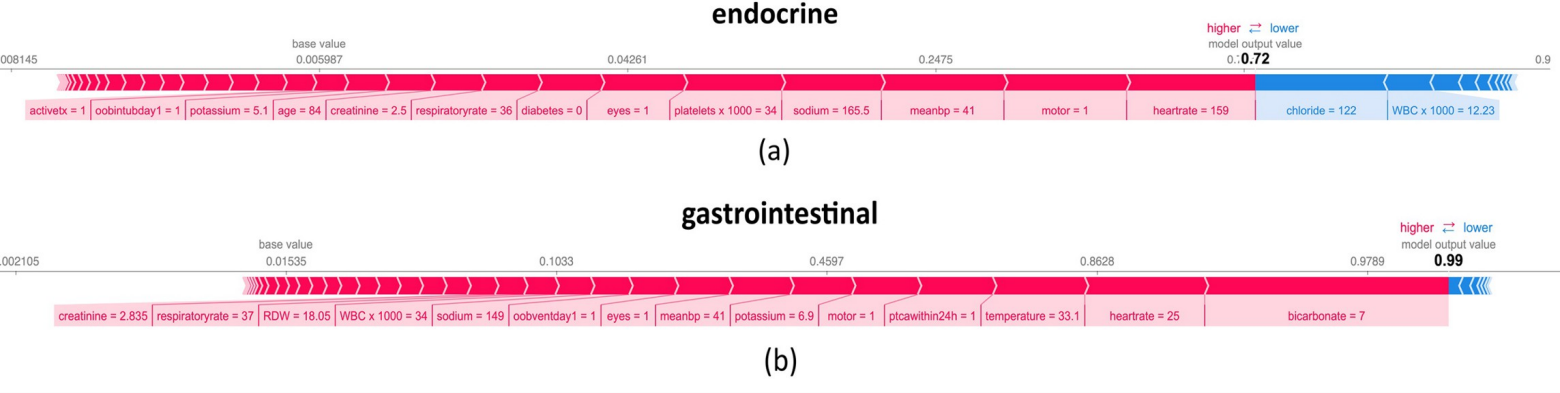
A detailed explanation of patient features with the highest mortality probability in endocrine and gastrointestinal disease groups using Shapley values (force plots).

In this section, patients with the highest mortality risk in each disease category were chosen as samples to assess their features’ attribution to mortality probability using Shapley values. The evaluation results are shown as force plots in Fig 4 for the endocrine and gastrointestinal disease groups and S8 and S9 Figs for the rest of the disease groups. Each feature’s contribution to the mortality prediction is shown in the form of an arrow. Each feature’s Shapley value either increases or decreases the mortality prediction results. Red arrows show features increasing the prediction results (i.e., mortality risk), and blue arrows show those decreasing the prediction values. These arrows balance each other on a point that is the prediction outcome for the chosen patient. The plots also show the base value, the mean mortality probability for the patients inside each related disease category.

S9 Fig part (l) shows a patient with a 98 percent mortality probability in the cardiovascular category. For this patient, the blood bicarbonate value of 9.4 mmol/L has the highest contribution to mortality. Other factors raising the patient’s mortality risk are mean blood pressure of 41 mmHg, the motor score of 1 (i.e., no movements in response to stimulus), platelets of 29.4 × 1000 (K/mcL), and heart rate of 147 beats per minute. Besides, RDW (red cell distribution width) of 19.4 percent, blood potassium of 5.58 mmol/L, respiratory rate of 40 breaths per minute, eyes score of 1 (i.e., no eye-opening), and blood calcium amount of 6.7 mg/dL were among other influential factors toward patients’ mortality. The patient’s age (53 years old), unit stay type of "transfer" (transferred to ICU), MCHC of 33.6 g/dL, WBC of 8.4 × 1000 K/mcL, and blood Sodium of 125.2 mmol/L were the main factors reducing the patient’s mortality risk. As expected, this patient has an expired status upon discharge from ICU.

As another example, the feature values of a patient with a 79 percent mortality probability in the oncology category are explained to understand the underlying factors contributing to the patient’s high predicted mortality probability. According to S8 Fig part (f), the bicarbonate value of 12 mEq/L is the most critical feature increasing the patient’s mortality risk. Mean blood pressure of 40 mmHg, BUN (blood urea nitrogen) of 54.6 mg/dL, blood calcium of 5.725 mg/dL, blood potassium of 6.1 mmol/L, and patient’s need for intubation and ventilation are among the other factors increasing the patient’s mortality risk. On the other hand, features reducing the patient’s mortality risk are MCV (mean corpuscular volume) of 115.7 fL, RBC of 1.967 mil/mcL, a verbal score of 5 (i.e., oriented), and a motor score of 6 (i.e., obeys commands for movement) which are all within their normal range of values. In addition, a seriously low value of platelets counts (9,700 << 150,000 per microliter of blood), a dangerously low body temperature (34.9 < 36.1 Celsius), a high blood creatinine level (1.7 > 1.35 mg/dL), and a high body weight of 105 Kg (high BMI based on the patient’s height) have all contributed to increasing the patients’ mortality probability and ultimate expiration upon discharge from the ICU. Observations from the force plots are mostly in line with the clinical knowledge in this area; thus, these tools are highly useful in explaining the decisions made by the black-box CatBoost model regarding the patients’ mortality/survival status. Similar conclusions can be derived for other patients across various disease categories (refer to S8 and S9 Figs).

In this section, we also applied LIME on the CatBoost classifier to examine and explain the importance of various features in the mortality prediction of some representative individuals.

**Fig 5:**
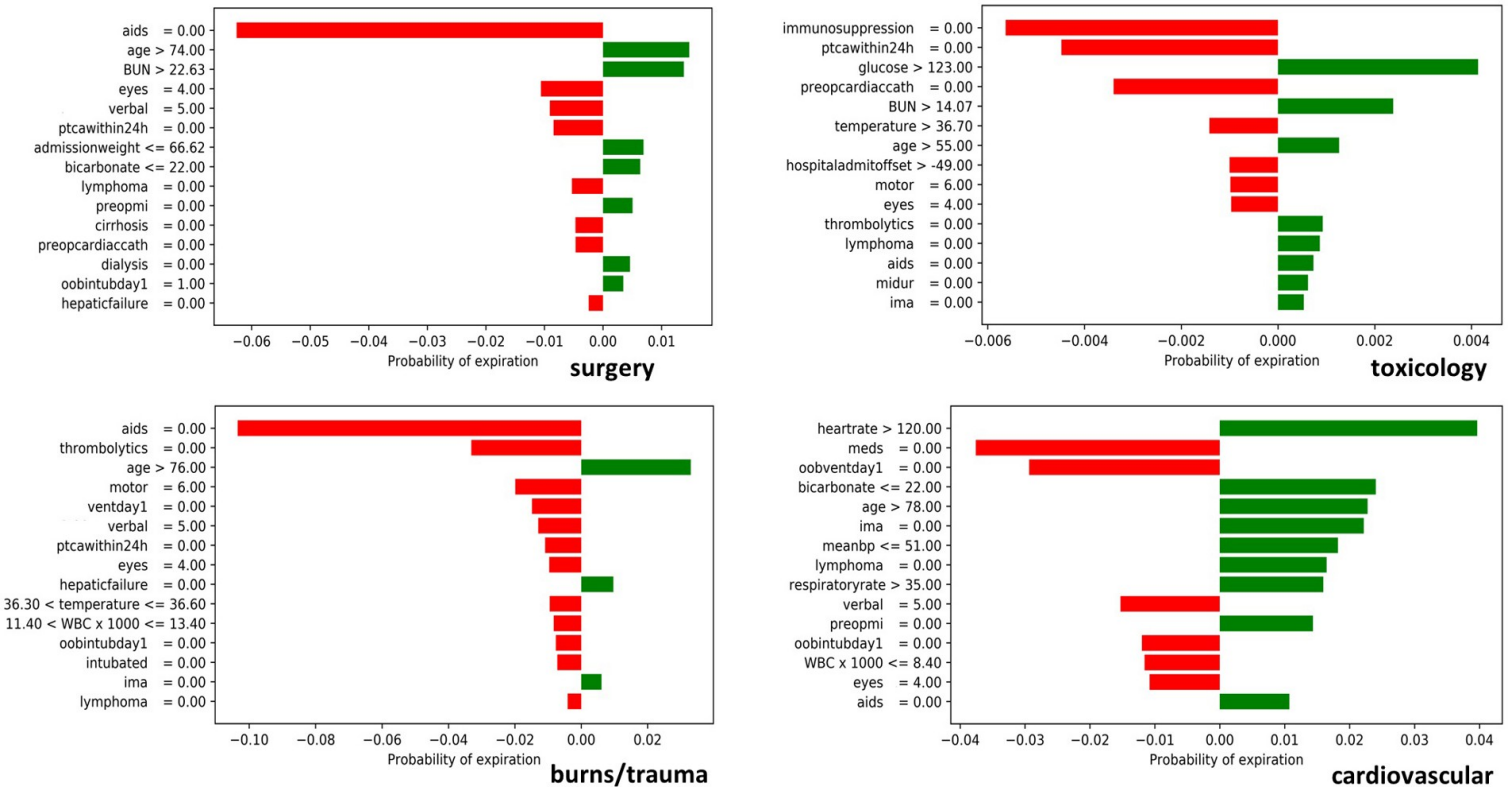
Feature importance of individual patients calculated using LIME in surgery, toxicology, burns-trauma, and cardiovascular disease groups.

We selected four patients from each disease group using a submodular pick algorithm [87] and applied LIME to explain their mortality prediction. The submodular pick algorithm picked a set of patient records with diverse characteristics to provide a global view of feature importance. Fig 5 shows LIME explanations for four patients in surgery, toxicology, burns/trauma, and cardiovascular disease groups (graphs for the rest of the disease groups are shown in S10 and S11 Figs). The vertical axis in these figures shows features at various levels of importance (highest to lowest from top to bottom) in mortality prediction and their respective values that increase or decrease the survival or expiration probabilities. The horizontal axis shows the likelihood of expiration. The bar lengths in the graphs correspond with the importance of each respective feature value in mortality prediction, such that longer bars show higher importance and shorter bars show lower importance, respectively. The bar colors in graphs represent if the associated feature value increases the survival (red) or expiration (green) probability.

The observations in Figs 5 and S10 and S11 are mostly in line with clinical findings; for example, not being diagnosed with aids for a patient in the burns/trauma group increases their survival probability and helps in keeping him "alive." The cardiovascular patient’s heart rate is over 120 beats per minute, which is the most important condition leading to their “expired” outcome. The leading feature of the patient’s expiration in the endocrine disease group is age (> 74), which conforms to medical evidence (older patients have a higher mortality probability than younger patients). There are few counterintuitive observations of feature effects in the plots; however, these features appear in lower importance ranking, and their effect values are negligible (e.g., aids and lymphoma in toxicology and cardiovascular groups).

### 4.4. Partial dependence (PD) and individual conditional expectation (ICE) plots for important features in the mortality prediction model

**Fig 6:**
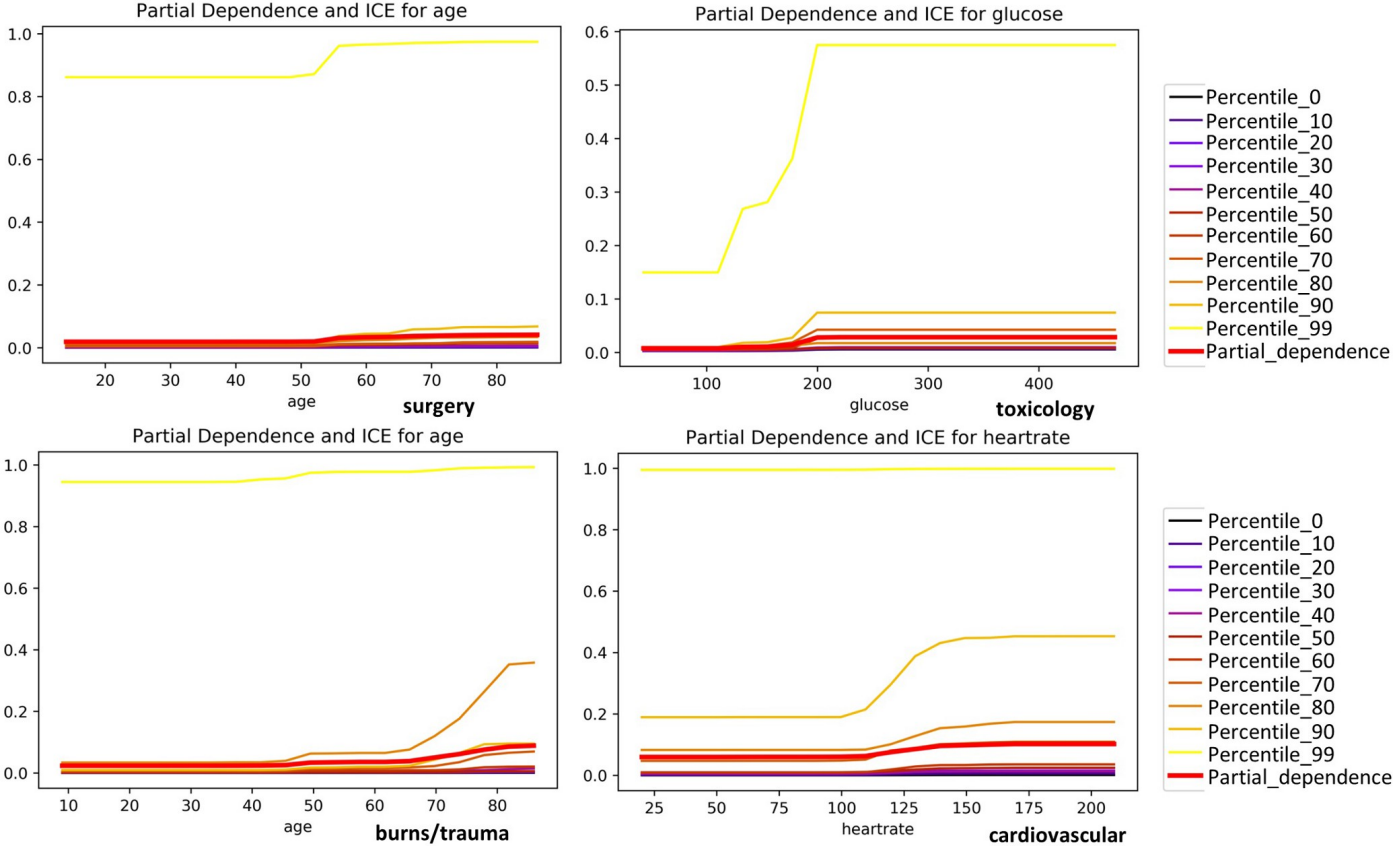
Partial dependence (PD) and individual conditional expectation (ICE) plots for the most important features in the prediction of ICU discharge status in surgery, toxicology, burns-trauma, and cardiovascular disease groups.

In this section, we created partial dependence (PD) and individual conditional expectation (ICE) plots for the most important features in the mortality prediction using the CatBoost model. The goal is to explain the monotonic behavior of each feature in patient mortality prediction across various disease groups. PD and ICE plots are calculated on the same scale and shown in the same plot. This helps to compare the average global prediction behavior of the entire model with the local prediction behavior of specific data points; this provides an opportunity to evaluate the reliability of average prediction behaviors using PD plots. We created PD and ICE plots for the most important feature at each percentile of the patient’s predicted mortality probability. Studying ICE plots for all patients is overwhelming; thus, plots were drawn only for the deciles of predicted mortality probabilities and are shown in Figs 6 and S12 and S13.

Figs 6 and S12 and S13 show the monotonic decreasing/increasing behavior of the most important features for patients at different percentiles of mortality probability (0 to 1) in each disease category. The graphs for the patients in all deciles show a steady increase in mortality probability when "age" increases. For patients at the 90th and 99th percentile of mortality probability, the increase rate in mortality probability with "age" is significantly higher than patients in lower mortality percentiles. The monotonic increase in heart rate from 100 to 130 beats per minute is associated with a significant rise in mortality probability of patients in cardiovascular and renal disease groups. For patients in the oncology group, the graphs for all percentiles show a sharp increase in the mortality probability when the respiratory rate increases from 30 to 35 per minute. For patients in the hematology group, a drop in platelets count to below 100,000 per microliter of blood significantly increases the mortality probability.

**Fig 7:**
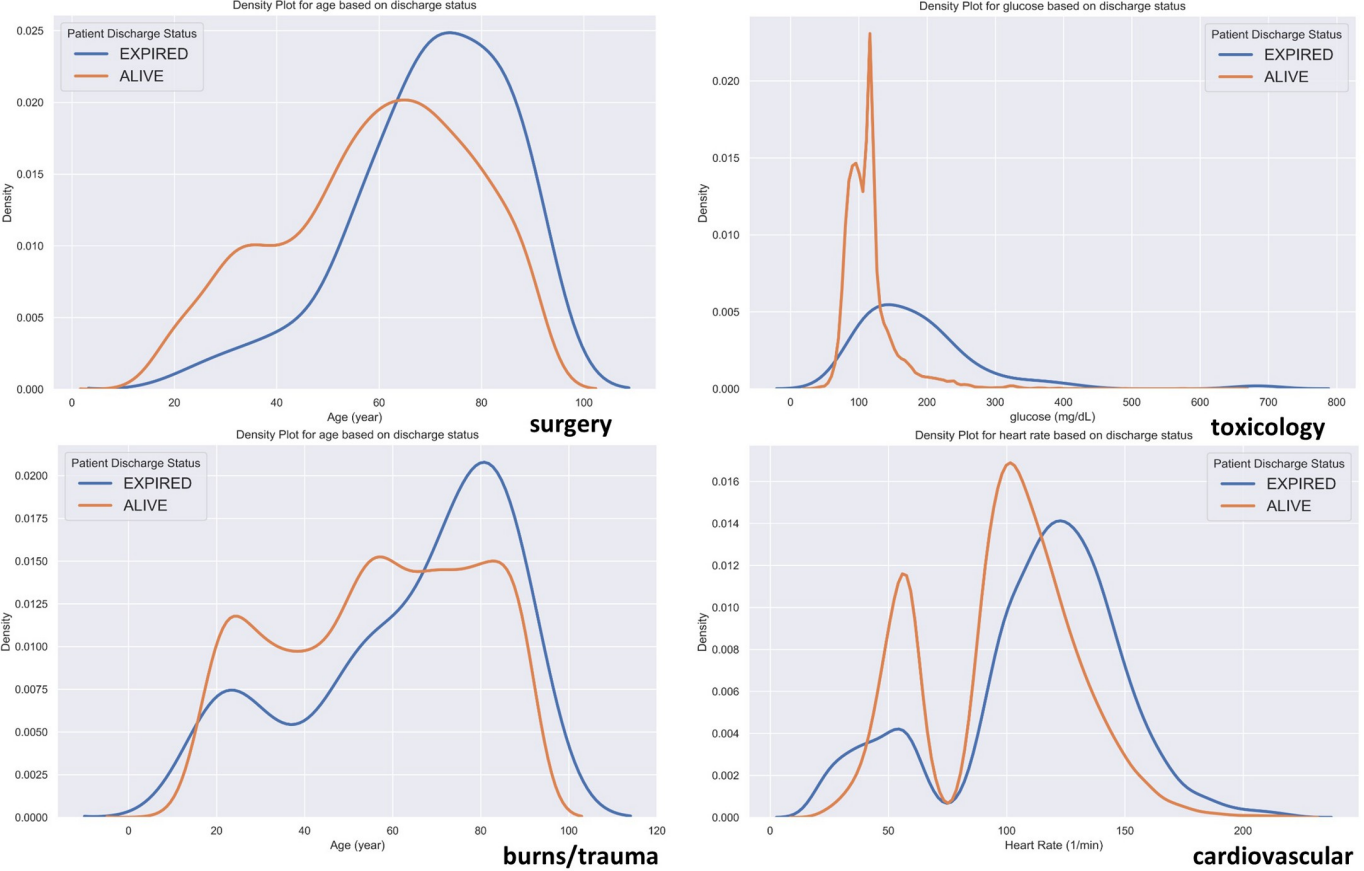
Bar and KDE plots for the most important features in predicting ICU discharge status for surgery, toxicology, burns-trauma, and cardiovascular disease groups.

It is also noteworthy to study the histograms of these features in mortality prediction (Figs 7 and S14 and S15). In this way, we can roughly verify the patterns observed in the PD and ICE plots.

In Figs 7 and S14 and S15, bar charts show histograms of categorical features, and Kernel Density Estimation (KDE) plots display the distributions of continuous features. The represented feature in each graph is the most important in predicting the patients’ discharge status in the mentioned disease group. The bar and KDE plots for the surgery, toxicology, burns/trauma, and cardiovascular disease groups are shown in Fig 7; plots for the rest of the disease groups are shown in S14 and S15 Figs. KDE plot is an effective tool for estimating the probability density function of continuous random variables. These plots show that there are distinguishably different distributions for the studied features based on their discharge status; this further confirms the usefulness of these features in explaining the variability of the data (class labels). The points where the KDE distributions of “Alive” and “Expired” patients intersect are key feature points in predicting patients’ discharge status. These value points conform to the previous observations in Figs 6 and S12 and S13 regarding the critical domains of feature values.

### 4.5. E-CatBoost: An improved model through feature selection

Patient records in ICU databases are stored by numerous variables, which do not have equal relevancy and importance in mortality prediction. The efficiency of ICU mortality prediction frameworks can be improved by developing models that can make accurate predictions provided with only a few input features. Medical data is expensive to collect and is not available at the same level for all patients; thus, reducing the amount of required input data to the prediction model is highly beneficial to the medical informatics area.

We studied the important features in mortality prediction in the previous sections. Based on the acquired results, we decided to limit the information provided to each model to the top ten most important features revealed by the SHAP values. Thus, we propose E-CatBoost, a highly optimized and efficient CatBoost-based model with improved features to predict patient mortality. E-CatBoost is considered efficient as it uses a significantly lower number of features (ten) to predict patient discharge status compared to the fifty features used by CatBoost. A lower number of required features for mortality prediction simplifies the patient evaluation process and helps medical experts make faster life-saving decisions about the patients’ treatment needs. For details regarding the required computation time to train and test the E-CatBoost and CatBoost models, refer to S27 Table. The validation results of the E-CatBoost model are shown in Table 6.

**Table 6:** Cross-validation (mean AUROC [standard deviation AUROC]) results of E-CatBoost model using 10-fold cross-validation.

Disease group	AUROC	Disease group	AUROC
**burns-trauma**	0.91 [0.03]	**neurologic**	0.89 [0.01]
**cardiovascular**	0.88[0.01]	**oncology**	0.86 [0.03]
**endocrine**	0.88 [0.01]	**pulmonary**	0.83 [0.02]
**gastrointestinal**	0.86 [0.02]	**renal**	0.84 [0.01]
**hematology**	0.86 [0.03]	**surgery**	0.88 [0.04]
**infectious diseases**	0.84 [0.02]	**toxicology**	0.91 [0.06]

Table 6 shows the AUROC results of the E-CatBoost model using the top ten most important features in each disease category. Based on the results, when we limit the input information to the model, AUROC values reduce between 0.01 and 0.04. Although this modification significantly reduces the amount of required information and the time spent gathering the data, AUROC scores decreased in negligible amounts. Although E-CatBoost uses only ten features to make mortality predictions, its performance on the entire patient population (AUROC = 0.87) is comparable to the state-of-the-art mortality prediction models tested on the eICU-CRD dataset (BiLSTM [74] = 0.84, community-based federated learning (CBFL) [27] = 0.70–0.75, Discharge Readiness Score (DRS) [75] = 0.86–0.94, and two-level attention-based LSTM [76] = 0.89). Also, E-CatBoost’s validation results showed that the model’s AUROC scores are 2 to 12 percent higher than the most commonly used illness severity scoring systems [10, 63–67] despite using a significantly lower amount of input information. For instance, APACHE IVa uses 142 patient variables for mortality prediction compared to only ten features used by E-CatBoost. Therefore, E-CatBoost accelerates the mortality prediction while keeping the prediction accuracy high.

## 5. Conclusion

In this study, we proposed an efficient machine learning model named E-CatBoost for the early prediction of the discharge status of patients admitted to the ICUs using the information available during the first 24 hours of admission. The model was trained and validated using the eICU Collaborative Research Database v2.0. The proposed method provides invaluable support for clinical decision-making and ICU management systems.

The study’s contributions are: 1- We proposed highly accurate and efficient mortality prediction models and compared their performance against a comprehensive list of baselines. We also interpreted the prediction of our proposed method using post-hoc explanation tools. Our proposed model (E-CatBoost) requires a significantly lower number of input features than the compared baseline models. 2- We developed and validated our models on the recently released eICU Collaborative Research Database v2.0, an extensive multi-center electronic database containing the data for over 200,000 ICU admissions at 208 hospitals throughout the United States. 3- Most previous studies [21–24] have proposed a single mortality prediction model for their entire patient population or have solely focused on a specific patient group. However, in this paper, we divided patients into twelve representative disease groups and tuned the baseline and proposed mortality prediction models for each group. 4- We identified the most critical risk factors in increasing the patient mortality probability in ICUs using Shapley values. Moreover, we studied how changing each underlying factor increases/decreases the mortality probability in each disease group using force, PD, and ICE plots. We found the critical ranges of values in each feature based on its correspondence with the mortality risk. Also, in addition to making global inferences, we studied the important factors and their critical ranges associated with heightened mortality probability in individual patients using LIME and force plots. This helps medical specialists to have a clear understanding of the decision-making process of our proposed model. 5- We performed feature selection on the entire set of biological, physiological, and medical variables and used the top ten most informative features to make predictions using the CatBoost model. In this way, we proposed E-CatBoost, a highly optimized and efficient CatBoost-based model with improved features to predict patient mortality.

The developed E-Catboost model has excellent potential to be generalized and used in clinical practice. The AUROC scores for the entire patient population were 0.91 [std:0.0038] for CatBoost and 0.87 [std:0.004] for E-CatBoost models; their performance was 7 to 18 (CatBoost) and 2 to 12 (E-CatBoost) percent higher than the most commonly used illness scoring systems [10, 63–67]. Also, the AUROC scores for the defined disease groups were 0.85 [std:0.02] to 0.92 [std:0.007] for CatBoost and 0.83 [std:0.02] to 0.91 [std:0.008] for E-CatBoost models; their performance was superior to illness severity scoring systems [10, 63–67] across all disease groups except pulmonary. Based on Shapley values, age, mean blood pressure, platelets count, heart and respiratory rate, blood glucose level, and having diabetes were found as the most important features for mortality prediction of patients in the defined disease groups (endocrine, gastrointestinal, hematology, infectious diseases, neurologic, oncology, pulmonary, renal, surgery, toxicology, burns-trauma, and cardiovascular).

In short, our model can visually explain to clinicians which patient characteristics are associated with high/low mortality risks and discuss the important domain values in each feature that increases/decreases the mortality probability. Age, heart rate, respiratory rate, blood urea nitrogen, blood creatinine level, and the verbal score features had the highest cross-disease importance in determining the patients’ discharge status.

It is advisable to externally validate E-CatBoost using other electronic ICU databases for future study. Additionally, an expansion of the proposed model (E-CatBoost) to predict the readmission probability and length of stay is desirable; it would lead to a more comprehensive and robust ICU monitoring system and could better suit the needs of clinical decision-makers.

## Supporting information

S1 Table Description of the patient tables used in the analysis. (DOCX)

S2 Table Full description of features used in the analysis. (DOCX)

S3 Table Descriptive statistics of numerical features in the burns-trauma disease group. (DOCX)

S4 Table Descriptive statistics of categorical features in the burns-trauma disease group. (DOCX)

S5 Table Descriptive statistics of numerical features in the cardiovascular disease group. (DOCX)

S6 Table Descriptive statistics of categorical features in the cardiovascular disease group. (DOCX)

S7 Table Descriptive statistics of numerical features in the endocrine disease group. (DOCX)

S8 Table Descriptive statistics of categorical features in the endocrine disease group. (DOCX)

S9 Table Descriptive statistics of numerical features in the gastrointestinal disease group. (DOCX)

S10 Table Descriptive statistics of categorical features in the gastrointestinal disease group. (DOCX)

S11 Table Descriptive statistics of numerical features in the hematology disease group. (DOCX)

S12 Table Descriptive statistics of categorical features in the hematology disease group. (DOCX)

S13 Table Descriptive statistics of numerical features in the infectious disease group. (DOCX)

S14 Table Descriptive statistics of categorical features in the infectious disease group. (DOCX)

S15 Table Descriptive statistics of numerical features in the neurologic disease group. (DOCX)

S16 Table Descriptive statistics of categorical features in the neurologic disease group. (DOCX)

S17 Table Descriptive statistics of numerical features in the oncology disease group. (DOCX)

S18 Table Descriptive statistics of categorical features in the oncology disease group. (DOCX)

S19 Table Descriptive statistics of numerical features in the pulmonary disease group. (DOCX)

S20 Table Descriptive statistics of categorical features in the pulmonary disease group. (DOCX)

S21 Table Descriptive statistics of numerical features in the renal disease group. (DOCX)

S22 Table Descriptive statistics of categorical features in the renal disease group. (DOCX)

S23 Table Descriptive statistics of numerical features in the surgery disease group. (DOCX)

S24 Table Descriptive statistics of categorical features in the surgery disease group. (DOCX)

S25 Table Descriptive statistics of numerical features in the toxicology disease group. (DOCX)

S26 Table Descriptive statistics of categorical features in the toxicology disease group. (DOCX)

S27 Table The required computation time (seconds) for training and testing E-CatBoost and CatBoost models. (DOCX)

S1 Fig Feature importance plots based on Shapley values for endocrine, gastrointestinal, hematology, and infectious disease groups. (TIF)

S2 Fig Feature importance plots based on Shapley values for surgery, toxicology, pulmonary, and renal disease groups. (TIF)

S3 Fig Force plots of the most important features in mortality prediction of patients in hematology and infectious disease groups. (TIF)

S4 Fig Force plots of the most important features in mortality prediction of patients in neurologic and oncology disease groups. (TIF)

S5 Fig Force plots of the most important features in mortality prediction of patients in pulmonary and renal disease groups. (TIF)

S6 Fig Force plots of the most important features in mortality prediction of patients in surgery and toxicology disease groups. (TIF)

S7 Fig Force plots of the most important features in mortality prediction of patients in burns-trauma and cardiovascular disease groups. (TIF)

S8 Fig Detailed explanation of features for patients with the highest mortality probability in hematology, infectious diseases, neurologic, oncology, and pulmonary disease groups using Shapley values (force plots). (TIF)

S9 Fig Detailed explanation of features for patients with the highest mortality probability in renal, surgery, toxicology, burns-trauma, and cardiovascular disease groups using Shapley values (force plots). (TIF)

S10 Fig Feature importance of individual patients calculated using LIME in endocrine, gastrointestinal, hematology, and infectious disease groups. (TIF)

S11 Fig Feature importance of individual patients calculated using LIME in neurologic, oncology, pulmonary, and renal disease groups. (TIF)

S12 Fig Partial dependence (PD) and individual conditional expectation (ICE) plots for the most important features in the prediction of ICU discharge status in endocrine, gastrointestinal, hematology, and infectious disease groups. (TIF)

S13 Fig Partial dependence (PD) and individual conditional expectation (ICE) plots for the most important features in the prediction of ICU discharge status in neurologic, oncology, pulmonary, and renal disease groups. (TIF)

S14 Fig Bar and KDE plots for the most important features in predicting ICU discharge status in endocrine, gastrointestinal, hematology, and infectious disease groups. (TIF)

S15 Fig Bar and KDE plots for the most important features in predicting ICU discharge status in neurologic, oncology, pulmonary, and renal disease groups. (TIF)
