# Supplementary material for: E-CatBoost: An efficient machine learning framework for predicting ICU mortality using the eICU Collaborative Research Database
Source: PLoS One. 2022 May 5;17(5):e0262895. doi: 10.1371/journal.pone.0262895 (PMC9070907; doi:10.1371/journal.pone.0262895)
Supplement: S1 Table — (DOCX) [file pone.0262895.s001.docx]

**S1 Table. Description of the patient tables used in the analysis**

| **Table Name** | **Description** |
| --- | --- |
| *Patient* | Details of patients' admission to ICU, demographics, and discharge status |
| *ApacheApsVar* | Data used for calculation of Acute Physiology Score (APS) III, which is a part of the APACHE system |
| *ApachePatientResult* | Details of ICU care process (e.g., actual ventilation days) |
| *ApachePredVar* | Variables used for creating APACHE predictions |
| *Diagnosis* | List and details of patients' diagnoses |
| *Lab* | Results of patients' standard laboratory tests |
