# Supplementary material for: E-CatBoost: An efficient machine learning framework for predicting ICU mortality using the eICU Collaborative Research Database
Source: PLoS One. 2022 May 5;17(5):e0262895. doi: 10.1371/journal.pone.0262895 (PMC9070907; doi:10.1371/journal.pone.0262895)
Supplement: S2 Table — (DOCX) [file pone.0262895.s002.docx]

**S2 Table. Full description of features used in the analysis**

| **Feature name** | **Values** | **Feature type** |
| --- | --- | --- |
| intubated | (No/Yes) Determines whether the patient is intubated at the time of worst ABG result | Categorical |
| dialysis | (No/Yes) Determines whether the patient is on dialysis | Categorical |
| temperature | Apache API's worst Celsius temperature | Numerical |
| respiratoryrate | Apache API's worst respiratory value | Numerical |
| sodium | Apache API's worst sodium lab level | Numerical |
| heartrate | Apache API's worst heart rate | Numerical |
| meanbp | Apache API's worst mean blood pressure | Numerical |
| hematocrit | Apache API's worst hematocrit lab level | Numerical |
| BUN | Apache API's worst BUN lab level | Numerical |
| glucose | Apache API's worst glucose lab level | Numerical |
| gender | Patient’s gender | Categorical |
| age | Patient’s age at the time of admission | Numerical |
| ethnicity | Patient’s reported ethnicity | Categorical |
| admissionheight | Patient’s height in "cm" | Numerical |
| unitstaytype | Patient’s unit type | Categorical |
| admissionweight | Patient’s weight in "kg" | Numerical |
| preopmi | (No/Yes) Determines whether the pre-operative myocardial infarction was present | Categorical |
| ptcawithin24h | (No/Yes) Determines whether the patient had PTCA within 24 hours of admission | Categorical |
| thrombolytics | (No/Yes) Determines whether the patient had thrombolytics | Categorical |
| aids | (No/Yes) Determines whether the patient had AIDS | Categorical |
| hepaticfailure | (No/Yes) Determines whether the patient had a hepatic failure | Categorical |
| lymphoma | (No/Yes) Determines whether the patient had lymphoma | Categorical |
| immunosuppression | (No/Yes) Determines whether the patient had immunosuppression | Categorical |
| cirrhosis | (No/Yes) Determines whether the patient had cirrhosis | Categorical |
| activetx | (No/Yes) Determines whether the patient had an active treatment | Categorical |
| midur | (No/Yes) Determines whether the patient had a MI within six months | Categorical |
| oobventday1 | (No/Yes) Determines whether the patient had been ventilated at any time during apache day | Categorical |
| oobintubday1 | (No/Yes) Determines whether the patient had been intubated at any time during apache day | Categorical |
| diabetes | (No/Yes) Determines whether the patient had diabetes | Categorical |
| unitadmitsource | Patient’s admission source | Categorical |
| ima | Determines whether 'Internal Mammary Artery Graft' field was selected in eCare | Categorical |
| calcium | Average calcium value (apache day) | Numerical |
| meds | NULL when not populated; set to 1 when “unable to score due to meds” is selected and no GCS score is available for the APACHE day; set to 0 when “unable to score due to meds” is not selected and a valid GCS score is set | Categorical |
| potassium | Average potassium value (apache day) | Numerical |
| chloride | Average chloride value (apache day) | Numerical |
| verbal | GCS verbal score from worst GCS | Numerical |
| motor | GCS motor score from worst GCS | Numerical |
| eyes | GCS eyes score from worst GCS | Numerical |
| anion gap | Average anion gap value (apache day) | Numerical |
| MCV | Average MCV value (apache day) | Numerical |
| diagnosisstring | Full pathstring of the diagnosis selected in eCareManager | Categorical |
| RBC | Average RBC value (apache day) | Numerical |
| bicarbonate | Average bicarbonate value (apache day) | Numerical |
| ventday1 | Indicates whether the patient was ventilated for the worst respiratory rate | Categorical |
| platelets × 1000 | Average platelets value (apache day) | Numerical |
| hospitaladmitoffset | Number of minutes from unit admit time that the patient was admitted to the hospital | Numerical |
| RDW | Average RDW value (apache day) | Numerical |
| WBC × 1000 | Average WBC value (apache day) | Numerical |
| Hgb | Average Hgb value (apache day) | Numerical |
| MCHC | Average MCHC value (apache day) | Numerical |
| MCH | Average MCH value (apache day) | Numerical |
| unittype | Patient’s admitted unit type | Categorical |
| creatinine | Average creatinine value (apache day) | Numerical |
| preopcardiaccath | Indicates whether the patient had a pre-operative cardiac catheterization | Categorical |
| actualicumortality | Patient’s mortality status upon discharge from the ICU | Categorical |
