# Supplementary material for: E-CatBoost: An efficient machine learning framework for predicting ICU mortality using the eICU Collaborative Research Database
Source: PLoS One. 2022 May 5;17(5):e0262895. doi: 10.1371/journal.pone.0262895 (PMC9070907; doi:10.1371/journal.pone.0262895)
Supplement: S3 Table — (DOCX) [file pone.0262895.s003.docx]

**S3 Table. Descriptive statistics of numerical features in the burns-trauma disease group**

| **Variable** | **Count** | **Mean** | **SD** | **Min.** | **Q_1_** | **Median** | **Q_3_** | **Max.** |
| --- | --- | --- | --- | --- | --- | --- | --- | --- |
| age | 4915 | 57.15 | 22.11 | 8.00 | 39.00 | 59.00 | 76.00 | 90.00 |
| admissionheight | 4915 | 171.82 | 11.24 | 62.00 | 165.00 | 172.70 | 180.30 | 210.80 |
| hospitaladmitoffset | 4915 | -1504.25 | 5721.07 | -156098.00 | -492.00 | -225.00 | -92.00 | 299.00 |
| admissionweight | 4915 | 81.42 | 22.91 | 14.40 | 65.80 | 78.80 | 93.00 | 302.80 |
| temperature | 4915 | 36.52 | 0.96 | 20.00 | 36.30 | 36.60 | 36.80 | 42.00 |
| respiratoryrate | 4915 | 22.25 | 14.19 | 4.00 | 10.00 | 22.00 | 32.00 | 60.00 |
| heartrate | 4915 | 103.15 | 30.47 | 20.00 | 91.00 | 106.00 | 122.00 | 211.00 |
| meanbp | 4915 | 86.83 | 40.28 | 40.00 | 55.00 | 67.00 | 122.00 | 200.00 |
| hematocrit | 4915 | 32.56 | 6.18 | 8.20 | 28.80 | 32.56 | 36.60 | 62.00 |
| verbal | 4915 | 3.83 | 1.59 | 1.00 | 3.00 | 5.00 | 5.00 | 5.00 |
| motor | 4915 | 5.36 | 1.40 | 1.00 | 5.00 | 6.00 | 6.00 | 6.00 |
| eyes | 4915 | 3.35 | 1.04 | 1.00 | 3.00 | 4.00 | 4.00 | 4.00 |
| potassium | 4915 | 4.09 | 0.51 | 2.50 | 3.80 | 4.09 | 4.30 | 7.90 |
| creatinine | 4915 | 1.12 | 0.91 | 0.19 | 0.71 | 0.91 | 1.12 | 15.20 |
| sodium | 4915 | 139.03 | 4.14 | 110.43 | 137.00 | 139.03 | 141.00 | 172.09 |
| BUN | 4915 | 19.38 | 14.16 | 2.00 | 11.00 | 16.00 | 21.00 | 150.00 |
| glucose | 4915 | 133.91 | 44.43 | 21.00 | 106.50 | 129.00 | 144.00 | 592.00 |
| chloride | 4915 | 105.81 | 5.23 | 68.75 | 103.00 | 105.81 | 109.00 | 142.55 |
| calcium | 4915 | 8.18 | 0.68 | 5.20 | 7.80 | 8.18 | 8.60 | 12.30 |
| Hgb | 4915 | 11.21 | 2.05 | 2.70 | 9.80 | 11.21 | 12.60 | 39.60 |
| WBC x 1000 | 4915 | 11.55 | 5.26 | 0.30 | 8.40 | 11.39 | 13.30 | 143.30 |
| platelets x 1000 | 4915 | 194.87 | 78.15 | 17.00 | 149.00 | 194.87 | 222.00 | 879.00 |
| RBC | 4915 | 3.71 | 0.66 | 0.92 | 3.27 | 3.71 | 4.14 | 6.46 |
| bicarbonate | 4915 | 24.18 | 3.57 | 3.00 | 22.00 | 24.18 | 26.00 | 50.00 |
| MCV | 4915 | 90.66 | 5.43 | 63.00 | 87.98 | 90.66 | 93.27 | 118.00 |
| MCHC | 4915 | 33.46 | 1.19 | 26.40 | 32.90 | 33.46 | 34.10 | 37.30 |
| MCH | 4915 | 30.31 | 1.94 | 17.80 | 29.60 | 30.31 | 31.10 | 40.80 |
| RDW | 4915 | 14.42 | 1.71 | 11.20 | 13.30 | 14.35 | 14.75 | 25.95 |
