# Supplementary material for: E-CatBoost: An efficient machine learning framework for predicting ICU mortality using the eICU Collaborative Research Database
Source: PLoS One. 2022 May 5;17(5):e0262895. doi: 10.1371/journal.pone.0262895 (PMC9070907; doi:10.1371/journal.pone.0262895)
Supplement: S4 Table — (DOCX) [file pone.0262895.s004.docx]

**S4 Table. Descriptive statistics of categorical features in the burns-trauma disease group**

| **Variable** | **Values** | **Frequency** | **Percentage Frequency** |
| --- | --- | --- | --- |
| intubated | No | 4132 | 84.07 |
|  | Yes | 783 | 15.93 |
| dialysis | No | 4843 | 98.54 |
|  | Yes | 72 | 1.46 |
| gender | Male | 3118 | 63.44 |
|  | Female | 1787 | 36.36 |
|  | Unknown/Other | 10 | 0.20 |
| ethnicity | Caucasian | 3923 | 79.82 |
|  | African American | 408 | 8.30 |
|  | Hispanic | 223 | 4.54 |
|  | Other/Unknown | 212 | 4.31 |
|  | Asian | 60 | 1.22 |
|  | Native American | 43 | 0.87 |
|  | Missing | 46 | 0.94 |
| unitstaytype | admit | 4449 | 90.52 |
|  | readmit | 236 | 4.80 |
|  | transfer | 230 | 4.68 |
| preopmi | No | 4915 | 100.00 |
|  | Yes | 0 | 0.00 |
| preopcardiaccath | No | 4915 | 100.00 |
|  | Yes | 0 | 0.00 |
| ptcawithin24h | No | 4876 | 99.21 |
|  | Yes | 39 | 0.79 |
| thrombolytics | No | 4910 | 99.90 |
|  | Yes | 5 | 0.10 |
| aids | No | 4913 | 99.96 |
|  | Yes | 2 | 0.04 |
| hepaticfailure | No | 4886 | 99.41 |
|  | Yes | 29 | 0.59 |
| lymphoma | No | 4908 | 99.86 |
|  | Yes | 7 | 0.14 |
| immunosuppression | No | 4877 | 99.23 |
|  | Yes | 38 | 0.77 |
| cirrhosis | No | 4881 | 99.31 |
|  | Yes | 34 | 0.69 |
| activetx | Yes | 2385 | 48.52 |
|  | No | 2530 | 51.48 |
| midur | No | 4899 | 99.67 |
|  | Yes | 16 | 0.33 |
| oobventday1 | No | 3275 | 66.63 |
|  | Yes | 1640 | 33.37 |
| oobintubday1 | No | 3392 | 69.01 |
|  | Yes | 1523 | 30.99 |
| diabetes | No | 4292 | 87.32 |
|  | Yes | 623 | 12.68 |
| unitadmitsource | Emergency Department | 3116 | 63.40 |
|  | Floor | 372 | 7.57 |
|  | Operating Room | 707 | 14.38 |
|  | Direct Admit | 158 | 3.21 |
|  | Recovery Room | 238 | 4.84 |
|  | Step-Down Unit (SDU) | 112 | 2.28 |
|  | Acute Care/Floor | 69 | 1.40 |
|  | Other Hospital | 58 | 1.18 |
|  | PACU | 61 | 1.24 |
|  | Other ICU | 13 | 0.26 |
|  | Chest Pain Center | 2 | 0.04 |
|  | ICU | 3 | 0.06 |
|  | ICU to SDU | 2 | 0.04 |
|  | Missing | 4 | 0.08 |
| ima | No | 4913 | 99.96 |
|  | Yes | 2 | 0.04 |
| meds | No | 4841 | 98.49 |
|  | Yes | 61 | 1.24 |
|  | Missing | 13 | 0.26 |
| ventday1 | No | 3638 | 74.02 |
|  | Yes | 1277 | 25.98 |
| unittype | Med-Surg ICU | 2912 | 59.25 |
|  | MICU | 172 | 3.50 |
|  | Cardiac ICU | 147 | 2.99 |
|  | SICU | 722 | 14.69 |
|  | CCU-CTICU | 77 | 1.57 |
|  | Neuro ICU | 819 | 16.66 |
|  | CTICU | 37 | 0.75 |
|  | CSICU | 29 | 0.59 |
| actualicumortality | Alive | 4660 | 94.81 |
|  | Expired | 255 | 5.19 |
