# Supplementary material for: E-CatBoost: An efficient machine learning framework for predicting ICU mortality using the eICU Collaborative Research Database
Source: PLoS One. 2022 May 5;17(5):e0262895. doi: 10.1371/journal.pone.0262895 (PMC9070907; doi:10.1371/journal.pone.0262895)
Supplement: S5 Table — (DOCX) [file pone.0262895.s005.docx]

**S5 Table. Descriptive statistics of numerical features in the cardiovascular disease group**

| **Variable** | **Count** | **Mean** | **SD** | **Min.** | **Q_1_** | **Median** | **Q_3_** | **Max.** |
| --- | --- | --- | --- | --- | --- | --- | --- | --- |
| age | 57174 | 66.17 | 15.10 | 0.00 | 57.00 | 68.00 | 78.00 | 90.00 |
| admissionheight | 57174 | 169.54 | 11.66 | 47.60 | 162.56 | 170.10 | 177.80 | 249.70 |
| hospitaladmitoffset | 57174 | -2218.80 | 7575.31 | -910552.00 | -1257.00 | -296.00 | -102.00 | 306.00 |
| admissionweight | 57174 | 84.89 | 25.97 | 0.40 | 67.60 | 81.30 | 97.90 | 396.90 |
| temperature | 57174 | 36.36 | 1.02 | 20.00 | 36.10 | 36.40 | 36.70 | 42.00 |
| respiratoryrate | 57174 | 25.03 | 14.71 | 4.00 | 11.00 | 27.00 | 35.00 | 60.00 |
| heartrate | 57174 | 99.38 | 32.47 | 20.00 | 81.00 | 103.00 | 120.00 | 220.00 |
| meanbp | 57174 | 84.57 | 43.23 | 40.00 | 51.00 | 62.00 | 124.00 | 200.00 |
| hematocrit | 57174 | 32.35 | 6.20 | 6.80 | 28.50 | 32.35 | 35.90 | 67.00 |
| verbal | 57174 | 4.05 | 1.52 | 1.00 | 4.00 | 5.00 | 5.00 | 5.00 |
| motor | 57174 | 5.47 | 1.31 | 1.00 | 6.00 | 6.00 | 6.00 | 6.00 |
| eyes | 57174 | 3.51 | 0.93 | 1.00 | 3.00 | 4.00 | 4.00 | 4.00 |
| potassium | 57174 | 4.12 | 0.56 | 1.80 | 3.80 | 4.10 | 4.40 | 9.60 |
| creatinine | 57174 | 1.65 | 1.61 | 0.10 | 0.83 | 1.19 | 1.65 | 28.11 |
| sodium | 57174 | 138.46 | 4.67 | 13.60 | 136.00 | 138.46 | 141.00 | 175.50 |
| BUN | 57174 | 28.03 | 20.18 | 1.00 | 15.00 | 23.00 | 33.00 | 256.00 |
| glucose | 57174 | 143.20 | 57.88 | 3.00 | 108.00 | 134.00 | 156.00 | 1169.00 |
| chloride | 57174 | 104.80 | 5.97 | 67.00 | 102.00 | 104.80 | 108.00 | 146.50 |
| calcium | 57174 | 8.29 | 0.73 | 3.20 | 7.90 | 8.29 | 8.70 | 18.60 |
| Hgb | 57174 | 10.92 | 2.05 | 2.70 | 9.50 | 10.92 | 12.20 | 23.77 |
| WBC x 1000 | 57174 | 12.46 | 8.14 | 0.00 | 8.40 | 11.90 | 14.03 | 374.54 |
| platelets x 1000 | 57174 | 196.27 | 86.30 | 1.67 | 145.00 | 196.27 | 227.50 | 1577.00 |
| RBC | 57174 | 3.69 | 0.69 | 0.92 | 3.23 | 3.69 | 4.10 | 8.00 |
| bicarbonate | 57174 | 23.94 | 4.41 | 4.00 | 22.00 | 23.94 | 26.00 | 55.00 |
| MCV | 57174 | 90.15 | 6.22 | 50.05 | 87.10 | 90.15 | 93.00 | 137.95 |
| MCHC | 57174 | 32.99 | 1.30 | 24.75 | 32.40 | 32.99 | 33.80 | 61.00 |
| MCH | 57174 | 29.74 | 2.28 | 12.60 | 29.00 | 29.74 | 30.90 | 57.00 |
| RDW | 57174 | 15.37 | 2.13 | 10.80 | 14.00 | 15.37 | 15.80 | 56.60 |
