# Supplementary material for: E-CatBoost: An efficient machine learning framework for predicting ICU mortality using the eICU Collaborative Research Database
Source: PLoS One. 2022 May 5;17(5):e0262895. doi: 10.1371/journal.pone.0262895 (PMC9070907; doi:10.1371/journal.pone.0262895)
Supplement: S6 Table — (DOCX) [file pone.0262895.s006.docx]

**S6 Table. Descriptive statistics of categorical features in the cardiovascular disease group**

| **Variable** | **Values** | **Frequency** | **Percentage Frequency** |
| --- | --- | --- | --- |
| intubated | No | 46851 | 81.94 |
|  | Yes | 10323 | 18.06 |
| dialysis | No | 54561 | 95.43 |
|  | Yes | 2613 | 4.57 |
| gender | Male | 32176 | 56.28 |
|  | Female | 24983 | 43.70 |
|  | Unknown/Other | 7 | 0.01 |
|  | Missing | 8 | 0.01 |
| ethnicity | Caucasian | 43379 | 75.87 |
|  | African American | 6485 | 11.34 |
|  | Hispanic | 2834 | 4.96 |
|  | Other/Unknown | 2677 | 4.68 |
|  | Asian | 867 | 1.52 |
|  | Native American | 327 | 0.57 |
|  | Missing | 605 | 1.06 |
| unitstaytype | admit | 51725 | 90.47 |
|  | readmit | 3516 | 6.15 |
|  | transfer | 1933 | 3.38 |
| preopmi | No | 56892 | 99.51 |
|  | Yes | 282 | 0.49 |
| preopcardiaccath | No | 56479 | 98.78 |
|  | Yes | 695 | 1.22 |
| ptcawithin24h | No | 50825 | 88.90 |
|  | Yes | 6349 | 11.10 |
| thrombolytics | No | 55457 | 97.00 |
|  | Yes | 1717 | 3.00 |
| aids | No | 57128 | 99.92 |
|  | Yes | 46 | 0.08 |
| hepaticfailure | No | 56462 | 98.75 |
|  | Yes | 712 | 1.25 |
| lymphoma | No | 56890 | 99.50 |
|  | Yes | 284 | 0.50 |
| immunosuppression | No | 55763 | 97.53 |
|  | Yes | 1411 | 2.47 |
| cirrhosis | No | 56317 | 98.50 |
|  | Yes | 857 | 1.50 |
| activetx | Yes | 38880 | 68.00 |
|  | No | 18294 | 32.00 |
| midur | No | 56347 | 98.55 |
|  | Yes | 827 | 1.45 |
| oobventday1 | No | 36445 | 63.74 |
|  | Yes | 20729 | 36.26 |
| oobintubday1 | No | 40568 | 70.96 |
|  | Yes | 16606 | 29.04 |
| diabetes | No | 42762 | 74.79 |
|  | Yes | 14412 | 25.21 |
| unitadmitsource | Emergency Department | 27261 | 47.68 |
|  | Floor | 8793 | 15.38 |
|  | Operating Room | 9522 | 16.65 |
|  | Direct Admit | 4116 | 7.20 |
|  | Recovery Room | 1694 | 2.96 |
|  | Step-Down Unit (SDU) | 1829 | 3.20 |
|  | Acute Care/Floor | 1776 | 3.11 |
|  | Other Hospital | 1252 | 2.19 |
|  | PACU | 352 | 0.62 |
|  | Other ICU | 217 | 0.38 |
|  | Chest Pain Center | 247 | 0.43 |
|  | ICU | 25 | 0.04 |
|  | ICU to SDU | 18 | 0.03 |
|  | Observation | 7 | 0.01 |
|  | Other | 2 | 0.00 |
|  | Missing | 63 | 0.11 |
| ima | No | 54029 | 94.50 |
|  | Yes | 3145 | 5.50 |
| meds | No | 56307 | 98.48 |
|  | Yes | 761 | 1.33 |
|  | Missing | 106 | 0.19 |
| ventday1 | No | 42464 | 74.27 |
|  | Yes | 14710 | 25.73 |
| unittype | Med-Surg ICU | 30562 | 53.45 |
|  | MICU | 4634 | 8.11 |
|  | Cardiac ICU | 5016 | 8.77 |
|  | SICU | 3341 | 5.84 |
|  | CCU-CTICU | 6047 | 10.58 |
|  | Neuro ICU | 1240 | 2.17 |
|  | CTICU | 3123 | 5.46 |
|  | CSICU | 3211 | 5.62 |
| actualicumortality | Alive | 53024 | 92.74 |
|  | Expired | 4150 | 7.26 |
