# Supplementary material for: E-CatBoost: An efficient machine learning framework for predicting ICU mortality using the eICU Collaborative Research Database
Source: PLoS One. 2022 May 5;17(5):e0262895. doi: 10.1371/journal.pone.0262895 (PMC9070907; doi:10.1371/journal.pone.0262895)
Supplement: S7 Table — (DOCX) [file pone.0262895.s007.docx]

**S7 Table. Descriptive statistics of numerical features in the endocrine disease group**

| **Variable** | **Count** | **Mean** | **SD** | **Min.** | **Q_1_** | **Median** | **Q_3_** | **Max.** |
| --- | --- | --- | --- | --- | --- | --- | --- | --- |
| age | 13667 | 59.53 | 18.45 | 0.00 | 48.00 | 62.00 | 74.00 | 90.00 |
| admissionheight | 13667 | 169.02 | 11.56 | 33.60 | 160.02 | 168.00 | 177.80 | 213.40 |
| hospitaladmitoffset | 13667 | -1697.65 | 5668.75 | -180999.00 | -591.00 | -242.00 | -99.00 | 316.00 |
| admissionweight | 13667 | 84.66 | 27.69 | 0.40 | 65.74 | 80.00 | 98.42 | 362.80 |
| temperature | 13667 | 36.41 | 0.94 | 20.00 | 36.20 | 36.50 | 36.70 | 41.80 |
| respiratoryrate | 13667 | 23.31 | 14.24 | 4.00 | 10.00 | 25.00 | 33.00 | 60.00 |
| heartrate | 13667 | 102.75 | 29.51 | 20.00 | 91.00 | 106.00 | 122.00 | 218.00 |
| meanbp | 13667 | 84.11 | 40.32 | 40.00 | 53.00 | 64.00 | 120.00 | 200.00 |
| hematocrit | 13667 | 32.57 | 5.80 | 9.20 | 29.30 | 32.57 | 35.70 | 62.30 |
| verbal | 13667 | 4.19 | 1.40 | 1.00 | 4.00 | 5.00 | 5.00 | 5.00 |
| motor | 13667 | 5.57 | 1.13 | 1.00 | 6.00 | 6.00 | 6.00 | 6.00 |
| eyes | 13667 | 3.57 | 0.85 | 1.00 | 3.50 | 4.00 | 4.00 | 4.00 |
| potassium | 13667 | 4.09 | 0.60 | 1.75 | 3.70 | 4.08 | 4.37 | 8.60 |
| creatinine | 13667 | 1.72 | 1.77 | 0.10 | 0.79 | 1.17 | 1.74 | 37.35 |
| sodium | 13667 | 137.77 | 5.72 | 104.33 | 135.00 | 137.77 | 140.33 | 180.20 |
| BUN | 13667 | 28.81 | 21.88 | 1.00 | 14.00 | 23.50 | 35.00 | 217.00 |
| glucose | 13667 | 184.43 | 93.86 | 3.00 | 125.50 | 172.00 | 213.50 | 1478.00 |
| chloride | 13667 | 104.44 | 6.82 | 67.00 | 101.00 | 104.44 | 108.00 | 150.67 |
| calcium | 13667 | 8.31 | 0.74 | 3.94 | 7.90 | 8.31 | 8.70 | 18.60 |
| Hgb | 13667 | 11.00 | 1.96 | 3.00 | 9.70 | 11.00 | 12.10 | 19.40 |
| WBC x 1000 | 13667 | 12.11 | 7.97 | 0.00 | 8.30 | 12.10 | 13.50 | 317.75 |
| platelets x 1000 | 13667 | 214.24 | 86.13 | 2.00 | 164.75 | 214.24 | 245.00 | 1173.50 |
| RBC | 13667 | 3.74 | 0.66 | 0.98 | 3.34 | 3.74 | 4.11 | 8.00 |
| bicarbonate | 13667 | 22.77 | 5.20 | 4.00 | 20.00 | 22.77 | 25.50 | 49.00 |
| MCV | 13667 | 89.01 | 6.10 | 54.10 | 86.00 | 89.01 | 92.00 | 137.95 |
| MCHC | 13667 | 33.20 | 1.38 | 25.90 | 32.55 | 33.20 | 34.00 | 42.40 |
| MCH | 13667 | 29.54 | 2.18 | 15.10 | 28.90 | 29.54 | 30.60 | 43.00 |
| RDW | 13667 | 15.10 | 2.05 | 10.70 | 13.80 | 15.10 | 15.40 | 47.30 |
