# Supplementary material for: E-CatBoost: An efficient machine learning framework for predicting ICU mortality using the eICU Collaborative Research Database
Source: PLoS One. 2022 May 5;17(5):e0262895. doi: 10.1371/journal.pone.0262895 (PMC9070907; doi:10.1371/journal.pone.0262895)
Supplement: S8 Table — (DOCX) [file pone.0262895.s008.docx]

**S8 Table. Descriptive statistics of categorical features in the endocrine disease group**

| **Variable** | **Values** | **Frequency** | **Percentage Frequency** |
| --- | --- | --- | --- |
| intubated | No | 11952 | 87.45 |
|  | Yes | 1715 | 12.55 |
| dialysis | No | 13016 | 95.24 |
|  | Yes | 651 | 4.76 |
| gender | Male | 6925 | 50.67 |
|  | Female | 6738 | 49.30 |
|  | Unknown/Other | 2 | 0.01 |
|  | Missing | 2 | 0.01 |
| ethnicity | Caucasian | 9222 | 67.48 |
|  | African American | 2082 | 15.23 |
|  | Hispanic | 1282 | 9.38 |
|  | Other/Unknown | 658 | 4.81 |
|  | Asian | 169 | 1.24 |
|  | Native American | 126 | 0.92 |
|  | Missing | 128 | 0.94 |
| unitstaytype | admit | 12651 | 92.57 |
|  | readmit | 636 | 4.65 |
|  | transfer | 380 | 2.78 |
| preopmi | No | 13625 | 99.69 |
|  | Yes | 42 | 0.31 |
| preopcardiaccath | No | 13566 | 99.26 |
|  | Yes | 101 | 0.74 |
| ptcawithin24h | No | 13092 | 95.79 |
|  | Yes | 575 | 4.21 |
| thrombolytics | No | 13563 | 99.24 |
|  | Yes | 104 | 0.76 |
| aids | No | 13657 | 99.93 |
|  | Yes | 10 | 0.07 |
| hepaticfailure | No | 13511 | 98.86 |
|  | Yes | 156 | 1.14 |
| lymphoma | No | 13621 | 99.66 |
|  | Yes | 46 | 0.34 |
| immunosuppression | No | 13418 | 98.18 |
|  | Yes | 249 | 1.82 |
| cirrhosis | No | 13462 | 98.50 |
|  | Yes | 205 | 1.50 |
| activetx | Yes | 7696 | 56.31 |
|  | No | 5971 | 43.69 |
| midur | No | 13541 | 99.08 |
|  | Yes | 126 | 0.92 |
| oobventday1 | No | 10269 | 75.14 |
|  | Yes | 3398 | 24.86 |
| oobintubday1 | No | 11027 | 80.68 |
|  | Yes | 2640 | 19.32 |
| diabetes | No | 6012 | 43.99 |
|  | Yes | 7655 | 56.01 |
| unitadmitsource | Emergency Department | 8728 | 63.86 |
|  | Floor | 1956 | 14.31 |
|  | Operating Room | 1179 | 8.63 |
|  | Direct Admit | 765 | 5.60 |
|  | Recovery Room | 324 | 2.37 |
|  | Step-Down Unit (SDU) | 213 | 1.56 |
|  | Acute Care/Floor | 211 | 1.54 |
|  | Other Hospital | 196 | 1.43 |
|  | PACU | 18 | 0.13 |
|  | Other ICU | 45 | 0.33 |
|  | Chest Pain Center | 14 | 0.10 |
|  | ICU | 4 | 0.03 |
|  | ICU to SDU | 5 | 0.04 |
|  | Observation | 1 | 0.01 |
|  | Missing | 8 | 0.06 |
| ima | No | 13300 | 97.31 |
|  | Yes | 367 | 2.69 |
| meds | No | 13476 | 98.60 |
|  | Yes | 167 | 1.22 |
|  | Missing | 24 | 0.18 |
| ventday1 | No | 11179 | 81.80 |
|  | Yes | 2488 | 18.20 |
| unittype | Med-Surg ICU | 8729 | 63.87 |
|  | MICU | 1151 | 8.42 |
|  | Cardiac ICU | 1150 | 8.41 |
|  | SICU | 749 | 5.48 |
|  | CCU-CTICU | 800 | 5.85 |
|  | Neuro ICU | 337 | 2.47 |
|  | CTICU | 639 | 4.68 |
|  | CSICU | 112 | 0.82 |
| actualicumortality | Alive | 13062 | 95.57 |
|  | Expired | 605 | 4.43 |
