# Supplementary material for: E-CatBoost: An efficient machine learning framework for predicting ICU mortality using the eICU Collaborative Research Database
Source: PLoS One. 2022 May 5;17(5):e0262895. doi: 10.1371/journal.pone.0262895 (PMC9070907; doi:10.1371/journal.pone.0262895)
Supplement: S9 Table — (DOCX) [file pone.0262895.s009.docx]

**S9 Table. Descriptive statistics of numerical features in the gastrointestinal disease group**

| **Variable** | **Count** | **Mean** | **SD** | **Min.** | **Q_1_** | **Median** | **Q_3_** | **Max.** |
| --- | --- | --- | --- | --- | --- | --- | --- | --- |
| age | 16694 | 64.07 | 16.61 | 0.00 | 53.00 | 65.00 | 77.00 | 90.00 |
| admissionheight | 16694 | 168.99 | 11.81 | 58.80 | 160.20 | 170.00 | 177.80 | 210.80 |
| hospitaladmitoffset | 16694 | -2767.09 | 7569.27 | -230202.00 | -1677.50 | -329.00 | -118.00 | 342.00 |
| admissionweight | 16694 | 82.78 | 27.14 | 0.40 | 64.90 | 78.00 | 95.25 | 396.90 |
| temperature | 16694 | 36.45 | 0.88 | 20.00 | 36.20 | 36.50 | 36.70 | 41.90 |
| respiratoryrate | 16694 | 24.55 | 14.46 | 4.00 | 11.00 | 27.00 | 35.00 | 60.00 |
| heartrate | 16694 | 106.90 | 29.78 | 20.00 | 94.00 | 109.00 | 126.00 | 220.00 |
| meanbp | 16694 | 81.67 | 41.53 | 40.00 | 50.00 | 62.00 | 118.00 | 200.00 |
| hematocrit | 16694 | 29.52 | 6.65 | 6.10 | 24.90 | 29.52 | 33.60 | 62.20 |
| verbal | 16694 | 4.13 | 1.45 | 1.00 | 4.00 | 5.00 | 5.00 | 5.00 |
| motor | 16694 | 5.59 | 1.10 | 1.00 | 6.00 | 6.00 | 6.00 | 6.00 |
| eyes | 16694 | 3.57 | 0.84 | 1.00 | 3.00 | 4.00 | 4.00 | 4.00 |
| potassium | 16694 | 4.09 | 0.60 | 1.90 | 3.70 | 4.09 | 4.40 | 8.40 |
| creatinine | 16694 | 1.59 | 1.58 | 0.11 | 0.77 | 1.12 | 1.61 | 27.23 |
| sodium | 16694 | 138.50 | 5.03 | 104.25 | 136.00 | 138.50 | 141.00 | 172.00 |
| BUN | 16694 | 29.82 | 23.18 | 1.00 | 14.00 | 24.33 | 36.38 | 263.33 |
| glucose | 16694 | 139.82 | 57.77 | 3.00 | 105.00 | 131.00 | 155.00 | 1293.00 |
| chloride | 16694 | 105.74 | 6.31 | 67.00 | 102.50 | 105.74 | 109.50 | 145.33 |
| calcium | 16694 | 7.98 | 0.76 | 3.20 | 7.57 | 7.98 | 8.40 | 18.60 |
| Hgb | 16694 | 10.15 | 2.08 | 3.05 | 8.55 | 10.10 | 11.40 | 20.30 |
| WBC x 1000 | 16694 | 12.48 | 8.76 | 0.00 | 7.90 | 11.70 | 14.55 | 359.10 |
| platelets x 1000 | 16694 | 196.40 | 101.67 | 1.00 | 133.00 | 196.00 | 237.00 | 1617.00 |
| RBC | 16694 | 3.44 | 0.71 | 0.92 | 2.94 | 3.44 | 3.85 | 7.05 |
| bicarbonate | 16694 | 22.99 | 4.57 | 4.00 | 20.50 | 22.99 | 25.50 | 47.00 |
| MCV | 16694 | 89.97 | 6.81 | 58.50 | 86.70 | 89.97 | 93.20 | 139.50 |
| MCHC | 16694 | 33.10 | 1.33 | 25.25 | 32.40 | 33.10 | 33.93 | 40.20 |
| MCH | 16694 | 29.79 | 2.53 | 16.40 | 28.90 | 29.79 | 31.00 | 48.80 |
| RDW | 16694 | 16.04 | 2.48 | 10.90 | 14.50 | 16.04 | 16.70 | 56.60 |
