# Supplementary material for: E-CatBoost: An efficient machine learning framework for predicting ICU mortality using the eICU Collaborative Research Database
Source: PLoS One. 2022 May 5;17(5):e0262895. doi: 10.1371/journal.pone.0262895 (PMC9070907; doi:10.1371/journal.pone.0262895)
Supplement: S10 Table — (DOCX) [file pone.0262895.s010.docx]

**S10 Table. Descriptive statistics of categorical features in the gastrointestinal disease group**

| **Variable** | **Values** | **Frequency** | **Percentage Frequency** |
| --- | --- | --- | --- |
| intubated | No | 14357 | 86.00 |
|  | Yes | 2337 | 14.00 |
| dialysis | No | 16114 | 96.53 |
|  | Yes | 580 | 3.47 |
| gender | Male | 8903 | 53.33 |
|  | Female | 7790 | 46.66 |
|  | Unknown/Other | 1 | 0.01 |
| ethnicity | Caucasian | 12728 | 76.24 |
|  | African American | 1702 | 10.20 |
|  | Hispanic | 947 | 5.67 |
|  | Other/Unknown | 785 | 4.70 |
|  | Asian | 241 | 1.44 |
|  | Native American | 148 | 0.89 |
|  | Missing | 143 | 0.86 |
| unitstaytype | admit | 14699 | 88.05 |
|  | readmit | 1257 | 7.53 |
|  | transfer | 738 | 4.42 |
| preopmi | No | 16687 | 99.96 |
|  | Yes | 7 | 0.04 |
| preopcardiaccath | No | 16678 | 99.90 |
|  | Yes | 16 | 0.10 |
| ptcawithin24h | No | 16412 | 98.31 |
|  | Yes | 282 | 1.69 |
| thrombolytics | No | 16672 | 99.87 |
|  | Yes | 22 | 0.13 |
| aids | No | 16681 | 99.92 |
|  | Yes | 13 | 0.08 |
| hepaticfailure | No | 15608 | 93.49 |
|  | Yes | 1086 | 6.51 |
| lymphoma | No | 16618 | 99.54 |
|  | Yes | 76 | 0.46 |
| immunosuppression | No | 16203 | 97.06 |
|  | Yes | 491 | 2.94 |
| cirrhosis | No | 15597 | 93.43 |
|  | Yes | 1097 | 6.57 |
| activetx | Yes | 10594 | 63.46 |
|  | No | 6100 | 36.54 |
| midur | No | 16594 | 99.40 |
|  | Yes | 100 | 0.60 |
| oobventday1 | No | 11966 | 71.68 |
|  | Yes | 4728 | 28.32 |
| oobintubday1 | No | 12590 | 75.42 |
|  | Yes | 4104 | 24.58 |
| diabetes | No | 13339 | 79.90 |
|  | Yes | 3355 | 20.10 |
| unitadmitsource | Emergency Department | 7877 | 47.18 |
|  | Floor | 2799 | 16.77 |
|  | Operating Room | 2604 | 15.60 |
|  | Direct Admit | 812 | 4.86 |
|  | Recovery Room | 818 | 4.90 |
|  | Step-Down Unit (SDU) | 486 | 2.91 |
|  | Acute Care/Floor | 524 | 3.14 |
|  | Other Hospital | 397 | 2.38 |
|  | PACU | 260 | 1.56 |
|  | Other ICU | 58 | 0.35 |
|  | Chest Pain Center | 19 | 0.11 |
|  | ICU | 12 | 0.07 |
|  | ICU to SDU | 6 | 0.04 |
|  | Observation | 2 | 0.01 |
|  | Missing | 20 | 0.12 |
| ima | No | 16660 | 99.80 |
|  | Yes | 34 | 0.20 |
| meds | No | 16487 | 98.76 |
|  | Yes | 185 | 1.11 |
|  | Missing | 22 | 0.13 |
| ventday1 | No | 13240 | 79.31 |
|  | Yes | 3454 | 20.69 |
| unittype | Med-Surg ICU | 11080 | 66.37 |
|  | MICU | 1601 | 9.59 |
|  | Cardiac ICU | 1084 | 6.49 |
|  | SICU | 1500 | 8.99 |
|  | CCU-CTICU | 668 | 4.00 |
|  | Neuro ICU | 296 | 1.77 |
|  | CTICU | 264 | 1.58 |
|  | CSICU | 201 | 1.20 |
| actualicumortality | Alive | 15515 | 92.94 |
|  | Expired | 1179 | 7.06 |
