# Supplementary material for: E-CatBoost: An efficient machine learning framework for predicting ICU mortality using the eICU Collaborative Research Database
Source: PLoS One. 2022 May 5;17(5):e0262895. doi: 10.1371/journal.pone.0262895 (PMC9070907; doi:10.1371/journal.pone.0262895)
Supplement: S11 Table — (DOCX) [file pone.0262895.s011.docx]

**S11 Table. Descriptive statistics of numerical features in the hematology disease group**

| **Variable** | **Count** | **Mean** | **SD** | **Min.** | **Q_1_** | **Median** | **Q_3_** | **Max.** |
| --- | --- | --- | --- | --- | --- | --- | --- | --- |
| age | 6957 | 65.05 | 17.03 | 7.00 | 54.00 | 67.00 | 79.00 | 90.00 |
| admissionheight | 6957 | 168.75 | 11.86 | 58.80 | 162.00 | 168.00 | 177.80 | 213.40 |
| hospitaladmitoffset | 6957 | -2900.59 | 8604.76 | -180999.00 | -1338.00 | -288.00 | -116.00 | 225.00 |
| admissionweight | 6957 | 80.92 | 25.47 | 0.40 | 64.20 | 76.88 | 92.60 | 349.20 |
| temperature | 6957 | 36.43 | 0.93 | 23.00 | 36.20 | 36.44 | 36.70 | 41.50 |
| respiratoryrate | 6957 | 24.89 | 14.21 | 4.00 | 11.00 | 27.00 | 35.00 | 60.00 |
| heartrate | 6957 | 105.42 | 30.49 | 20.00 | 92.00 | 108.00 | 125.00 | 218.00 |
| meanbp | 6957 | 79.32 | 40.59 | 40.00 | 50.00 | 60.00 | 115.00 | 200.00 |
| hematocrit | 6957 | 27.58 | 6.53 | 7.20 | 23.10 | 27.58 | 30.90 | 67.00 |
| verbal | 6957 | 4.07 | 1.45 | 1.00 | 4.00 | 5.00 | 5.00 | 5.00 |
| motor | 6957 | 5.52 | 1.19 | 1.00 | 6.00 | 6.00 | 6.00 | 6.00 |
| eyes | 6957 | 3.55 | 0.88 | 1.00 | 3.00 | 4.00 | 4.00 | 4.00 |
| potassium | 6957 | 4.13 | 0.63 | 2.20 | 3.73 | 4.10 | 4.40 | 8.90 |
| creatinine | 6957 | 1.84 | 1.86 | 0.10 | 0.80 | 1.29 | 1.90 | 26.15 |
| sodium | 6957 | 138.24 | 5.02 | 107.50 | 136.00 | 138.24 | 141.00 | 168.00 |
| BUN | 6957 | 33.28 | 24.66 | 1.00 | 16.00 | 28.00 | 41.00 | 251.00 |
| glucose | 6957 | 136.06 | 55.79 | 20.00 | 103.00 | 128.00 | 149.00 | 890.00 |
| chloride | 6957 | 105.14 | 6.16 | 68.50 | 102.00 | 105.14 | 109.00 | 136.00 |
| calcium | 6957 | 8.11 | 0.78 | 5.00 | 7.70 | 8.11 | 8.55 | 18.60 |
| Hgb | 6957 | 9.48 | 2.01 | 2.20 | 8.05 | 9.30 | 10.47 | 22.60 |
| WBC x 1000 | 6957 | 13.37 | 18.33 | 0.00 | 7.33 | 11.20 | 14.30 | 456.45 |
| platelets x 1000 | 6957 | 181.29 | 109.47 | 1.00 | 110.00 | 181.00 | 227.00 | 1807.00 |
| RBC | 6957 | 3.24 | 0.70 | 0.89 | 2.76 | 3.24 | 3.61 | 7.12 |
| bicarbonate | 6957 | 23.38 | 4.77 | 4.00 | 21.00 | 23.38 | 26.00 | 49.00 |
| MCV | 6957 | 89.31 | 7.26 | 59.00 | 86.00 | 89.31 | 92.90 | 139.50 |
| MCHC | 6957 | 33.07 | 1.44 | 24.40 | 32.33 | 33.07 | 34.00 | 61.00 |
| MCH | 6957 | 29.55 | 2.70 | 17.00 | 28.60 | 29.55 | 30.93 | 57.00 |
| RDW | 6957 | 16.66 | 2.81 | 11.50 | 14.88 | 16.66 | 17.45 | 60.90 |
