# Supplementary material for: E-CatBoost: An efficient machine learning framework for predicting ICU mortality using the eICU Collaborative Research Database
Source: PLoS One. 2022 May 5;17(5):e0262895. doi: 10.1371/journal.pone.0262895 (PMC9070907; doi:10.1371/journal.pone.0262895)
Supplement: S12 Table — (DOCX) [file pone.0262895.s012.docx]

**S12 Table. Descriptive statistics of categorical features in the hematology disease group**

| **Variable** | **Values** | **Frequency** | **Percentage Frequency** |
| --- | --- | --- | --- |
| intubated | No | 5980 | 85.96 |
|  | Yes | 977 | 14.04 |
| dialysis | No | 6672 | 95.90 |
|  | Yes | 285 | 4.10 |
| gender | Male | 3653 | 52.51 |
|  | Female | 3304 | 47.49 |
| ethnicity | Caucasian | 4823 | 69.33 |
|  | African American | 838 | 12.05 |
|  | Hispanic | 830 | 11.93 |
|  | Other/Unknown | 292 | 4.20 |
|  | Asian | 104 | 1.49 |
|  | Native American | 43 | 0.62 |
|  | Missing | 27 | 0.39 |
| unitstaytype | admit | 6279 | 90.25 |
|  | readmit | 514 | 7.39 |
|  | transfer | 164 | 2.36 |
| preopmi | No | 6937 | 99.71 |
|  | Yes | 20 | 0.29 |
| preopcardiaccath | No | 6916 | 99.41 |
|  | Yes | 41 | 0.59 |
| ptcawithin24h | No | 6760 | 97.17 |
|  | Yes | 197 | 2.83 |
| thrombolytics | No | 6942 | 99.78 |
|  | Yes | 15 | 0.22 |
| aids | No | 6950 | 99.90 |
|  | Yes | 7 | 0.10 |
| hepaticfailure | No | 6720 | 96.59 |
|  | Yes | 237 | 3.41 |
| lymphoma | No | 6883 | 98.94 |
|  | Yes | 74 | 1.06 |
| immunosuppression | No | 6598 | 94.84 |
|  | Yes | 359 | 5.16 |
| cirrhosis | No | 6704 | 96.36 |
|  | Yes | 253 | 3.64 |
| activetx | Yes | 4449 | 63.95 |
|  | No | 2508 | 36.05 |
| midur | No | 6905 | 99.25 |
|  | Yes | 52 | 0.75 |
| oobventday1 | No | 5099 | 73.29 |
|  | Yes | 1858 | 26.71 |
| oobintubday1 | No | 5456 | 78.42 |
|  | Yes | 1501 | 21.58 |
| diabetes | No | 5649 | 81.20 |
|  | Yes | 1308 | 18.80 |
| unitadmitsource | Emergency Department | 3952 | 56.81 |
|  | Floor | 1497 | 21.52 |
|  | Operating Room | 479 | 6.89 |
|  | Direct Admit | 338 | 4.86 |
|  | Recovery Room | 153 | 2.20 |
|  | Step-Down Unit (SDU) | 172 | 2.47 |
|  | Acute Care/Floor | 164 | 2.36 |
|  | Other Hospital | 140 | 2.01 |
|  | PACU | 16 | 0.23 |
|  | Other ICU | 22 | 0.32 |
|  | Chest Pain Center | 11 | 0.16 |
|  | ICU | 2 | 0.03 |
|  | ICU to SDU | 5 | 0.07 |
|  | Missing | 6 | 0.09 |
| ima | No | 6878 | 98.86 |
|  | Yes | 79 | 1.14 |
| meds | No | 6857 | 98.56 |
|  | Yes | 98 | 1.41 |
|  | Missing | 2 | 0.03 |
| ventday1 | No | 5616 | 80.72 |
|  | Yes | 1341 | 19.28 |
| unittype | Med-Surg ICU | 4475 | 64.32 |
|  | MICU | 523 | 7.52 |
|  | Cardiac ICU | 638 | 9.17 |
|  | SICU | 555 | 7.98 |
|  | CCU-CTICU | 305 | 4.38 |
|  | Neuro ICU | 153 | 2.20 |
|  | CTICU | 244 | 3.51 |
|  | CSICU | 64 | 0.92 |
| actualicumortality | Alive | 6363 | 91.46 |
|  | Expired | 594 | 8.54 |
