# Supplementary material for: E-CatBoost: An efficient machine learning framework for predicting ICU mortality using the eICU Collaborative Research Database
Source: PLoS One. 2022 May 5;17(5):e0262895. doi: 10.1371/journal.pone.0262895 (PMC9070907; doi:10.1371/journal.pone.0262895)
Supplement: S13 Table — (DOCX) [file pone.0262895.s013.docx]

**S13 Table. Descriptive statistics of numerical features in the infectious disease group**

| **Variable** | **Count** | **Mean** | **SD** | **Min.** | **Q_1_** | **Median** | **Q_3_** | **Max.** |
| --- | --- | --- | --- | --- | --- | --- | --- | --- |
| age | 9508 | 63.92 | 17.13 | 15.00 | 54.00 | 66.00 | 77.00 | 90.00 |
| admissionheight | 9508 | 168.67 | 12.03 | 57.50 | 160.00 | 168.00 | 177.80 | 213.00 |
| hospitaladmitoffset | 9508 | -2689.81 | 7546.12 | -180999.00 | -1339.25 | -305.00 | -124.00 | 306.00 |
| admissionweight | 9508 | 84.01 | 29.54 | 0.40 | 64.40 | 78.10 | 97.43 | 334.20 |
| temperature | 9508 | 36.62 | 1.19 | 20.80 | 36.30 | 36.60 | 36.90 | 41.90 |
| respiratoryrate | 9508 | 27.10 | 14.17 | 4.00 | 12.00 | 29.00 | 36.00 | 60.00 |
| heartrate | 9508 | 109.17 | 29.06 | 20.00 | 96.00 | 111.00 | 128.00 | 217.00 |
| meanbp | 9508 | 78.28 | 41.36 | 40.00 | 49.00 | 59.00 | 114.00 | 200.00 |
| hematocrit | 9508 | 31.04 | 5.93 | 7.70 | 27.20 | 31.04 | 34.40 | 62.00 |
| verbal | 9508 | 3.89 | 1.52 | 1.00 | 3.00 | 5.00 | 5.00 | 5.00 |
| motor | 9508 | 5.45 | 1.18 | 1.00 | 5.50 | 6.00 | 6.00 | 6.00 |
| eyes | 9508 | 3.48 | 0.90 | 1.00 | 3.00 | 4.00 | 4.00 | 4.00 |
| potassium | 9508 | 4.06 | 0.62 | 1.82 | 3.65 | 4.00 | 4.40 | 8.60 |
| creatinine | 9508 | 1.82 | 1.75 | 0.10 | 0.80 | 1.26 | 1.96 | 19.80 |
| sodium | 9508 | 138.15 | 5.39 | 104.50 | 135.00 | 138.15 | 141.00 | 174.00 |
| BUN | 9508 | 31.33 | 23.21 | 1.00 | 15.00 | 26.00 | 39.00 | 217.00 |
| glucose | 9508 | 144.99 | 62.98 | 3.00 | 105.00 | 132.00 | 163.00 | 1169.00 |
| chloride | 9508 | 105.03 | 6.66 | 60.67 | 101.00 | 105.03 | 109.00 | 143.67 |
| calcium | 9508 | 8.05 | 0.77 | 4.60 | 7.60 | 8.05 | 8.50 | 16.30 |
| Hgb | 9508 | 10.39 | 1.97 | 3.70 | 9.00 | 10.39 | 11.60 | 22.70 |
| WBC x 1000 | 9508 | 14.18 | 10.79 | 0.00 | 8.80 | 13.10 | 16.80 | 357.12 |
| platelets x 1000 | 9508 | 207.64 | 107.42 | 1.00 | 140.88 | 203.00 | 254.00 | 1229.50 |
| RBC | 9508 | 3.53 | 0.67 | 1.25 | 3.07 | 3.53 | 3.95 | 7.19 |
| bicarbonate | 9508 | 23.20 | 4.96 | 6.00 | 20.00 | 23.20 | 26.00 | 53.00 |
| MCV | 9508 | 90.30 | 7.09 | 59.50 | 86.50 | 90.30 | 94.00 | 129.20 |
| MCHC | 9508 | 32.76 | 1.39 | 25.25 | 32.00 | 32.76 | 33.60 | 42.40 |
| MCH | 9508 | 29.59 | 2.51 | 16.70 | 28.40 | 29.59 | 31.00 | 44.97 |
| RDW | 9508 | 15.86 | 2.36 | 11.00 | 14.25 | 15.80 | 16.70 | 32.70 |
| anion gap | 9508 | 10.97 | 3.91 | -8.00 | 9.00 | 10.97 | 12.20 | 42.30 |
