# Supplementary material for: E-CatBoost: An efficient machine learning framework for predicting ICU mortality using the eICU Collaborative Research Database
Source: PLoS One. 2022 May 5;17(5):e0262895. doi: 10.1371/journal.pone.0262895 (PMC9070907; doi:10.1371/journal.pone.0262895)
Supplement: S14 Table — (DOCX) [file pone.0262895.s014.docx]

**S14 Table. Descriptive statistics of categorical features in the infectious disease group**

| **Variable** | **Values** | **Frequency** | **Percentage Frequency** |
| --- | --- | --- | --- |
| intubated | No | 8037 | 84.53 |
|  | Yes | 1471 | 15.47 |
| dialysis | No | 9064 | 95.33 |
|  | Yes | 444 | 4.67 |
| gender | Male | 4961 | 52.18 |
|  | Female | 4546 | 47.81 |
|  | Unknown/Other | 1 | 0.01 |
| ethnicity | Caucasian | 7151 | 75.21 |
|  | African American | 949 | 9.98 |
|  | Hispanic | 703 | 7.39 |
|  | Other/Unknown | 456 | 4.80 |
|  | Asian | 130 | 1.37 |
|  | Native American | 59 | 0.62 |
|  | Missing | 60 | 0.63 |
| unitstaytype | admit | 8373 | 88.06 |
|  | readmit | 708 | 7.45 |
|  | transfer | 427 | 4.49 |
| preopmi | No | 9506 | 99.98 |
|  | Yes | 2 | 0.02 |
| preopcardiaccath | No | 9502 | 99.94 |
|  | Yes | 6 | 0.06 |
| ptcawithin24h | No | 9381 | 98.66 |
|  | Yes | 127 | 1.34 |
| thrombolytics | No | 9490 | 99.81 |
|  | Yes | 18 | 0.19 |
| aids | No | 9443 | 99.32 |
|  | Yes | 65 | 0.68 |
| hepaticfailure | No | 9377 | 98.62 |
|  | Yes | 131 | 1.38 |
| lymphoma | No | 9448 | 99.37 |
|  | Yes | 60 | 0.63 |
| immunosuppression | No | 9149 | 96.22 |
|  | Yes | 359 | 3.78 |
| cirrhosis | No | 9324 | 98.06 |
|  | Yes | 184 | 1.94 |
| activetx | Yes | 6114 | 64.30 |
|  | No | 3394 | 35.70 |
| midur | No | 9467 | 99.57 |
|  | Yes | 41 | 0.43 |
| oobventday1 | No | 6252 | 65.76 |
|  | Yes | 3256 | 34.24 |
| oobintubday1 | No | 7050 | 74.15 |
|  | Yes | 2458 | 25.85 |
| diabetes | No | 7336 | 77.16 |
|  | Yes | 2172 | 22.84 |
| unitadmitsource | Emergency Department | 5495 | 57.79 |
|  | Floor | 1959 | 20.60 |
|  | Operating Room | 598 | 6.29 |
|  | Direct Admit | 407 | 4.28 |
|  | Recovery Room | 207 | 2.18 |
|  | Step-Down Unit (SDU) | 265 | 2.79 |
|  | Acute Care/Floor | 264 | 2.78 |
|  | Other Hospital | 221 | 2.32 |
|  | PACU | 38 | 0.40 |
|  | Other ICU | 29 | 0.31 |
|  | Chest Pain Center | 8 | 0.08 |
|  | ICU | 3 | 0.03 |
|  | ICU to SDU | 5 | 0.05 |
|  | Missing | 9 | 0.09 |
| ima | No | 9500 | 99.92 |
|  | Yes | 8 | 0.08 |
| meds | No | 9366 | 98.51 |
|  | Yes | 130 | 1.37 |
|  | Missing | 12 | 0.13 |
| ventday1 | No | 7042 | 74.06 |
|  | Yes | 2466 | 25.94 |
| unittype | Med-Surg ICU | 6424 | 67.56 |
|  | MICU | 779 | 8.19 |
|  | Cardiac ICU | 945 | 9.94 |
|  | SICU | 489 | 5.14 |
|  | CCU-CTICU | 539 | 5.67 |
|  | Neuro ICU | 146 | 1.54 |
|  | CTICU | 119 | 1.25 |
|  | CSICU | 67 | 0.70 |
| actualicumortality | Alive | 8709 | 91.60 |
|  | Expired | 799 | 8.40 |
