# Supplementary material for: E-CatBoost: An efficient machine learning framework for predicting ICU mortality using the eICU Collaborative Research Database
Source: PLoS One. 2022 May 5;17(5):e0262895. doi: 10.1371/journal.pone.0262895 (PMC9070907; doi:10.1371/journal.pone.0262895)
Supplement: S15 Table — (DOCX) [file pone.0262895.s015.docx]

**S15 Table. Descriptive statistics of numerical features in the neurologic disease group**

| **Variable** | **Count** | **Mean** | **SD** | **Min.** | **Q_1_** | **Median** | **Q_3_** | **Max.** |
| --- | --- | --- | --- | --- | --- | --- | --- | --- |
| age | 25779 | 61.48 | 17.66 | 0.00 | 50.00 | 63.00 | 75.00 | 90.00 |
| admissionheight | 25779 | 169.48 | 11.62 | 33.60 | 162.60 | 170.00 | 177.80 | 227.30 |
| hospitaladmitoffset | 25779 | -1926.61 | 6263.43 | -243160.00 | -729.00 | -247.00 | -83.00 | 255.00 |
| admissionweight | 25779 | 81.74 | 24.12 | 0.50 | 65.50 | 78.10 | 94.30 | 302.80 |
| temperature | 25779 | 36.48 | 0.93 | 20.00 | 36.20 | 36.50 | 36.80 | 42.10 |
| respiratoryrate | 25779 | 23.78 | 14.71 | 4.00 | 10.00 | 25.00 | 34.00 | 60.00 |
| heartrate | 25779 | 98.36 | 31.25 | 20.00 | 70.00 | 103.00 | 119.00 | 220.00 |
| meanbp | 25779 | 93.57 | 42.90 | 40.00 | 56.00 | 72.00 | 129.00 | 200.00 |
| hematocrit | 25779 | 34.12 | 5.70 | 7.30 | 31.30 | 34.12 | 37.30 | 61.50 |
| verbal | 25779 | 3.57 | 1.63 | 1.00 | 2.00 | 4.00 | 5.00 | 5.00 |
| motor | 25779 | 5.22 | 1.42 | 1.00 | 5.00 | 6.00 | 6.00 | 6.00 |
| eyes | 25779 | 3.24 | 1.06 | 1.00 | 3.00 | 4.00 | 4.00 | 4.00 |
| potassium | 25779 | 3.99 | 0.52 | 1.75 | 3.70 | 3.99 | 4.20 | 7.45 |
| creatinine | 25779 | 1.32 | 1.33 | 0.10 | 0.70 | 0.97 | 1.32 | 30.34 |
| sodium | 25779 | 138.97 | 4.94 | 100.00 | 137.00 | 138.97 | 141.00 | 180.20 |
| BUN | 25779 | 22.35 | 17.75 | 1.00 | 12.00 | 18.00 | 24.00 | 256.00 |
| glucose | 25779 | 137.29 | 54.77 | 3.00 | 104.50 | 131.00 | 147.50 | 1169.00 |
| chloride | 25779 | 105.12 | 5.99 | 67.00 | 102.00 | 105.12 | 108.00 | 150.67 |
| calcium | 25779 | 8.39 | 0.68 | 4.90 | 8.10 | 8.39 | 8.80 | 16.30 |
| Hgb | 25779 | 11.49 | 1.98 | 3.20 | 10.30 | 11.49 | 12.70 | 22.70 |
| WBC x 1000 | 25779 | 11.36 | 6.77 | 0.00 | 8.00 | 11.30 | 12.80 | 290.35 |
| platelets x 1000 | 25779 | 205.79 | 81.26 | 1.00 | 162.00 | 205.79 | 236.00 | 1617.00 |
| RBC | 25779 | 3.84 | 0.66 | 0.92 | 3.48 | 3.84 | 4.23 | 8.00 |
| bicarbonate | 25779 | 24.18 | 4.02 | 4.00 | 22.00 | 24.18 | 26.00 | 62.00 |
| MCV | 25779 | 90.41 | 6.07 | 56.70 | 87.80 | 90.41 | 93.00 | 137.95 |
| MCHC | 25779 | 33.19 | 1.26 | 24.70 | 32.70 | 33.19 | 33.90 | 39.00 |
| MCH | 25779 | 30.00 | 2.23 | 15.70 | 29.20 | 30.00 | 31.00 | 45.80 |
| RDW | 25779 | 14.90 | 2.00 | 0.00 | 13.60 | 14.90 | 15.10 | 56.60 |
