# Supplementary material for: E-CatBoost: An efficient machine learning framework for predicting ICU mortality using the eICU Collaborative Research Database
Source: PLoS One. 2022 May 5;17(5):e0262895. doi: 10.1371/journal.pone.0262895 (PMC9070907; doi:10.1371/journal.pone.0262895)
Supplement: S16 Table — (DOCX) [file pone.0262895.s016.docx]

**S16 Table. Descriptive statistics of categorical features in the neurologic disease group**

| **Variable** | **Values** | **Frequency** | **Percentage Frequency** |
| --- | --- | --- | --- |
| intubated | No | 22041 | 85.50 |
|  | Yes | 3738 | 14.50 |
| dialysis | No | 25112 | 97.41 |
|  | Yes | 667 | 2.59 |
| gender | Male | 13532 | 52.49 |
|  | Female | 12238 | 47.47 |
|  | Unknown/Other | 8 | 0.03 |
|  | Missing | 1 | 0.00 |
| ethnicity | Caucasian | 19428 | 75.36 |
|  | African American | 2867 | 11.12 |
|  | Hispanic | 1437 | 5.57 |
|  | Other/Unknown | 1217 | 4.72 |
|  | Asian | 389 | 1.51 |
|  | Native American | 246 | 0.95 |
|  | Missing | 195 | 0.76 |
| unitstaytype | admit | 23232 | 90.12 |
|  | readmit | 1583 | 6.14 |
|  | transfer | 964 | 3.74 |
| preopmi | No | 25773 | 99.98 |
|  | Yes | 6 | 0.02 |
| preopcardiaccath | No | 25764 | 99.94 |
|  | Yes | 15 | 0.06 |
| ptcawithin24h | No | 25354 | 98.35 |
|  | Yes | 425 | 1.65 |
| thrombolytics | No | 25724 | 99.79 |
|  | Yes | 55 | 0.21 |
| aids | No | 25749 | 99.88 |
|  | Yes | 30 | 0.12 |
| hepaticfailure | No | 25368 | 98.41 |
|  | Yes | 411 | 1.59 |
| lymphoma | No | 25685 | 99.64 |
|  | Yes | 94 | 0.36 |
| immunosuppression | No | 25279 | 98.06 |
|  | Yes | 500 | 1.94 |
| cirrhosis | No | 25266 | 98.01 |
|  | Yes | 513 | 1.99 |
| activetx | Yes | 13893 | 53.89 |
|  | No | 11886 | 46.11 |
| midur | No | 25688 | 99.65 |
|  | Yes | 91 | 0.35 |
| oobventday1 | No | 18015 | 69.88 |
|  | Yes | 7764 | 30.12 |
| oobintubday1 | No | 19050 | 73.90 |
|  | Yes | 6729 | 26.10 |
| diabetes | No | 21061 | 81.70 |
|  | Yes | 4718 | 18.30 |
| unitadmitsource | Emergency Department | 13951 | 54.12 |
|  | Floor | 3476 | 13.48 |
|  | Operating Room | 2322 | 9.01 |
|  | Direct Admit | 1795 | 6.96 |
|  | Recovery Room | 1585 | 6.15 |
|  | Step-Down Unit (SDU) | 654 | 2.54 |
|  | Acute Care/Floor | 635 | 2.46 |
|  | Other Hospital | 843 | 3.27 |
|  | PACU | 301 | 1.17 |
|  | Other ICU | 138 | 0.54 |
|  | Chest Pain Center | 23 | 0.09 |
|  | ICU | 28 | 0.11 |
|  | ICU to SDU | 3 | 0.01 |
|  | Observation | 1 | 0.00 |
|  | Missing | 24 | 0.09 |
| ima | No | 25726 | 99.79 |
|  | Yes | 53 | 0.21 |
| meds | No | 25411 | 98.57 |
|  | Yes | 318 | 1.23 |
|  | Missing | 50 | 0.19 |
| ventday1 | No | 19725 | 76.52 |
|  | Yes | 6054 | 23.48 |
| unittype | Med-Surg ICU | 13948 | 54.11 |
|  | MICU | 1962 | 7.61 |
|  | Cardiac ICU | 1495 | 5.80 |
|  | SICU | 1862 | 7.22 |
|  | CCU-CTICU | 866 | 3.36 |
|  | Neuro ICU | 5120 | 19.86 |
|  | CTICU | 330 | 1.28 |
|  | CSICU | 196 | 0.76 |
| actualicumortality | Alive | 24268 | 94.14 |
|  | Expired | 1511 | 5.86 |
