# Supplementary material for: E-CatBoost: An efficient machine learning framework for predicting ICU mortality using the eICU Collaborative Research Database
Source: PLoS One. 2022 May 5;17(5):e0262895. doi: 10.1371/journal.pone.0262895 (PMC9070907; doi:10.1371/journal.pone.0262895)
Supplement: S17 Table — (DOCX) [file pone.0262895.s017.docx]

**S17 Table. Descriptive statistics of numerical features in the oncology disease group**

| **Variable** | **Count** | **Mean** | **SD** | **Min.** | **Q_1_** | **Median** | **Q_3_** | **Max.** |
| --- | --- | --- | --- | --- | --- | --- | --- | --- |
| age | 3710 | 64.99 | 13.20 | 19.00 | 57.00 | 66.00 | 75.00 | 90.00 |
| admissionheight | 3710 | 169.76 | 11.45 | 61.00 | 162.60 | 170.20 | 177.80 | 203.20 |
| hospitaladmitoffset | 3710 | -3313.10 | 7719.62 | -109190.00 | -2808.00 | -544.50 | -205.25 | 205.00 |
| admissionweight | 3710 | 78.83 | 23.17 | 0.40 | 62.60 | 75.50 | 90.70 | 349.20 |
| temperature | 3710 | 36.48 | 0.89 | 20.30 | 36.20 | 36.50 | 36.70 | 41.30 |
| respiratoryrate | 3710 | 24.22 | 14.48 | 4.00 | 10.00 | 27.00 | 35.00 | 60.00 |
| heartrate | 3710 | 108.20 | 29.66 | 20.00 | 96.00 | 110.00 | 126.00 | 218.00 |
| meanbp | 3710 | 81.91 | 40.48 | 40.00 | 52.00 | 63.00 | 118.00 | 200.00 |
| hematocrit | 3710 | 29.96 | 5.64 | 8.90 | 26.40 | 29.96 | 33.10 | 58.80 |
| verbal | 3710 | 4.16 | 1.45 | 1.00 | 4.00 | 5.00 | 5.00 | 5.00 |
| motor | 3710 | 5.64 | 1.04 | 1.00 | 6.00 | 6.00 | 6.00 | 6.00 |
| eyes | 3710 | 3.60 | 0.81 | 1.00 | 3.50 | 4.00 | 4.00 | 4.00 |
| potassium | 3710 | 4.14 | 0.59 | 1.90 | 3.80 | 4.14 | 4.40 | 8.40 |
| creatinine | 3710 | 1.26 | 1.14 | 0.10 | 0.70 | 0.96 | 1.26 | 14.86 |
| sodium | 3710 | 137.45 | 4.77 | 104.33 | 135.00 | 137.45 | 140.00 | 173.80 |
| BUN | 3710 | 24.38 | 18.79 | 2.00 | 13.00 | 20.00 | 27.33 | 171.00 |
| glucose | 3710 | 142.95 | 50.24 | 14.00 | 112.00 | 139.00 | 158.00 | 844.00 |
| chloride | 3710 | 104.11 | 5.83 | 68.00 | 101.00 | 104.11 | 107.50 | 141.00 |
| calcium | 3710 | 8.16 | 0.83 | 3.94 | 7.70 | 8.16 | 8.60 | 16.60 |
| Hgb | 3710 | 10.13 | 1.85 | 2.70 | 8.80 | 10.13 | 11.29 | 21.90 |
| WBC x 1000 | 3710 | 13.23 | 15.62 | 0.00 | 7.70 | 11.70 | 14.20 | 317.75 |
| platelets x 1000 | 3710 | 199.08 | 106.80 | 2.00 | 134.00 | 199.08 | 243.00 | 902.00 |
| RBC | 3710 | 3.42 | 0.65 | 0.91 | 2.99 | 3.42 | 3.80 | 7.40 |
| bicarbonate | 3710 | 23.93 | 4.56 | 4.50 | 22.00 | 23.93 | 26.00 | 53.00 |
| MCV | 3710 | 89.81 | 6.40 | 63.50 | 86.50 | 89.81 | 93.00 | 127.00 |
| MCHC | 3710 | 33.05 | 1.25 | 25.80 | 32.40 | 33.05 | 33.80 | 38.10 |
| MCH | 3710 | 29.68 | 2.23 | 20.00 | 28.90 | 29.68 | 30.80 | 43.00 |
| RDW | 3710 | 16.17 | 2.55 | 11.40 | 14.40 | 16.17 | 17.09 | 34.10 |
