# Supplementary material for: E-CatBoost: An efficient machine learning framework for predicting ICU mortality using the eICU Collaborative Research Database
Source: PLoS One. 2022 May 5;17(5):e0262895. doi: 10.1371/journal.pone.0262895 (PMC9070907; doi:10.1371/journal.pone.0262895)
Supplement: S18 Table — (DOCX) [file pone.0262895.s018.docx]

**S18 Table. Descriptive statistics of categorical features in the oncology disease group**

| **Variable** | **Values** | **Frequency** | **Percentage Frequency** |
| --- | --- | --- | --- |
| intubated | No | 3244 | 87.44 |
|  | Yes | 466 | 12.56 |
| dialysis | No | 3668 | 98.87 |
|  | Yes | 42 | 1.13 |
| gender | Male | 1987 | 53.56 |
|  | Female | 1723 | 46.44 |
| ethnicity | Caucasian | 2765 | 74.53 |
|  | African American | 439 | 11.83 |
|  | Hispanic | 286 | 7.71 |
|  | Other/Unknown | 118 | 3.18 |
|  | Asian | 73 | 1.97 |
|  | Native American | 10 | 0.27 |
|  | Missing | 19 | 0.51 |
| unitstaytype | admit | 3333 | 89.84 |
|  | readmit | 285 | 7.68 |
|  | transfer | 92 | 2.48 |
| preopmi | No | 3708 | 99.95 |
|  | Yes | 2 | 0.05 |
| preopcardiaccath | No | 3708 | 99.95 |
|  | Yes | 2 | 0.05 |
| ptcawithin24h | No | 3643 | 98.19 |
|  | Yes | 67 | 1.81 |
| thrombolytics | No | 3699 | 99.70 |
|  | Yes | 11 | 0.30 |
| aids | No | 3707 | 99.92 |
|  | Yes | 3 | 0.08 |
| hepaticfailure | No | 3654 | 98.49 |
|  | Yes | 56 | 1.51 |
| lymphoma | No | 3626 | 97.74 |
|  | Yes | 84 | 2.26 |
| immunosuppression | No | 2950 | 79.51 |
|  | Yes | 760 | 20.49 |
| cirrhosis | No | 3667 | 98.84 |
|  | Yes | 43 | 1.16 |
| activetx | Yes | 2180 | 58.76 |
|  | No | 1530 | 41.24 |
| midur | No | 3692 | 99.51 |
|  | Yes | 18 | 0.49 |
| oobventday1 | No | 2617 | 70.54 |
|  | Yes | 1093 | 29.46 |
| oobintubday1 | No | 2858 | 77.04 |
|  | Yes | 852 | 22.96 |
| diabetes | No | 3141 | 84.66 |
|  | Yes | 569 | 15.34 |
| unitadmitsource | Emergency Department | 1203 | 32.43 |
|  | Floor | 762 | 20.54 |
|  | Operating Room | 843 | 22.72 |
|  | Direct Admit | 176 | 4.74 |
|  | Recovery Room | 410 | 11.05 |
|  | Step-Down Unit (SDU) | 97 | 2.61 |
|  | Acute Care/Floor | 80 | 2.16 |
|  | Other Hospital | 55 | 1.48 |
|  | PACU | 58 | 1.56 |
|  | Other ICU | 18 | 0.49 |
|  | ICU to SDU | 3 | 0.08 |
|  | Missing | 5 | 0.13 |
| ima | No | 3705 | 99.87 |
|  | Yes | 5 | 0.13 |
| meds | No | 3662 | 98.71 |
|  | Yes | 45 | 1.21 |
|  | Missing | 3 | 0.08 |
| ventday1 | No | 2906 | 78.33 |
|  | Yes | 804 | 21.67 |
| unittype | Med-Surg ICU | 2259 | 60.89 |
|  | MICU | 334 | 9.00 |
|  | Cardiac ICU | 217 | 5.85 |
|  | SICU | 420 | 11.32 |
|  | CCU-CTICU | 168 | 4.53 |
|  | Neuro ICU | 166 | 4.47 |
|  | CTICU | 91 | 2.45 |
|  | CSICU | 55 | 1.48 |
| actualicumortality | Alive | 3346 | 90.19 |
|  | Expired | 364 | 9.81 |
