# Supplementary material for: E-CatBoost: An efficient machine learning framework for predicting ICU mortality using the eICU Collaborative Research Database
Source: PLoS One. 2022 May 5;17(5):e0262895. doi: 10.1371/journal.pone.0262895 (PMC9070907; doi:10.1371/journal.pone.0262895)
Supplement: S19 Table — (DOCX) [file pone.0262895.s019.docx]

**S19 Table. Descriptive statistics of numerical features in the pulmonary disease group**

| **Variable** | **Count** | **Mean** | **SD** | **Min.** | **Q_1_** | **Median** | **Q_3_** | **Max.** |
| --- | --- | --- | --- | --- | --- | --- | --- | --- |
| age | 32831 | 64.76 | 16.11 | 0.00 | 55.00 | 67.00 | 77.00 | 90.00 |
| admissionheight | 32831 | 168.76 | 11.61 | 52.40 | 160.00 | 168.00 | 177.80 | 218.00 |
| hospitaladmitoffset | 32831 | -2782.72 | 7871.02 | -252904.00 | -1816.00 | -287.00 | -98.00 | 310.00 |
| admissionweight | 32831 | 84.47 | 29.07 | 0.40 | 64.90 | 79.50 | 98.20 | 396.90 |
| temperature | 32831 | 36.44 | 1.02 | 20.00 | 36.20 | 36.44 | 36.80 | 42.30 |
| respiratoryrate | 32831 | 27.63 | 14.43 | 4.00 | 13.00 | 29.00 | 37.00 | 60.00 |
| heartrate | 32831 | 106.36 | 29.81 | 20.00 | 94.00 | 109.00 | 125.00 | 220.00 |
| meanbp | 32831 | 84.11 | 42.26 | 40.00 | 51.00 | 63.00 | 122.00 | 200.00 |
| hematocrit | 32831 | 32.64 | 6.14 | 6.80 | 28.90 | 32.64 | 36.00 | 67.00 |
| verbal | 32831 | 3.58 | 1.72 | 1.00 | 1.00 | 5.00 | 5.00 | 5.00 |
| motor | 32831 | 5.24 | 1.45 | 1.00 | 5.00 | 6.00 | 6.00 | 6.00 |
| eyes | 32831 | 3.28 | 1.06 | 1.00 | 3.00 | 4.00 | 4.00 | 4.00 |
| potassium | 32831 | 4.14 | 0.60 | 1.80 | 3.75 | 4.13 | 4.43 | 9.70 |
| creatinine | 32831 | 1.53 | 1.47 | 0.10 | 0.76 | 1.11 | 1.57 | 26.00 |
| sodium | 32831 | 138.62 | 4.94 | 13.60 | 136.00 | 138.62 | 141.00 | 173.67 |
| BUN | 32831 | 28.10 | 19.76 | 1.00 | 15.00 | 24.00 | 34.00 | 229.00 |
| glucose | 32831 | 148.03 | 59.02 | 3.00 | 111.00 | 139.50 | 164.00 | 825.75 |
| chloride | 32831 | 103.68 | 6.40 | 67.00 | 100.00 | 103.68 | 107.00 | 145.33 |
| calcium | 32831 | 8.31 | 1.05 | 3.20 | 7.90 | 8.31 | 8.70 | 141.00 |
| Hgb | 32831 | 10.84 | 2.03 | 2.70 | 9.40 | 10.84 | 12.10 | 22.60 |
| WBC x 1000 | 32831 | 12.70 | 8.99 | 0.00 | 8.40 | 12.05 | 14.50 | 469.80 |
| platelets x 1000 | 32831 | 210.14 | 94.95 | 2.00 | 153.50 | 210.14 | 245.50 | 1807.00 |
| RBC | 32831 | 3.68 | 0.69 | 0.92 | 3.23 | 3.68 | 4.09 | 8.00 |
| bicarbonate | 32831 | 25.25 | 5.41 | 3.00 | 22.00 | 25.25 | 27.67 | 62.00 |
| MCV | 32831 | 90.72 | 6.64 | 54.10 | 87.40 | 90.72 | 94.00 | 137.95 |
| MCHC | 32831 | 32.62 | 1.41 | 25.55 | 32.00 | 32.62 | 33.40 | 61.00 |
| MCH | 32831 | 29.58 | 2.38 | 15.10 | 28.70 | 29.58 | 30.80 | 57.00 |
| RDW | 32831 | 15.65 | 2.21 | 0.00 | 14.20 | 15.65 | 16.20 | 47.30 |
