# Supplementary material for: E-CatBoost: An efficient machine learning framework for predicting ICU mortality using the eICU Collaborative Research Database
Source: PLoS One. 2022 May 5;17(5):e0262895. doi: 10.1371/journal.pone.0262895 (PMC9070907; doi:10.1371/journal.pone.0262895)
Supplement: S20 Table — (DOCX) [file pone.0262895.s020.docx]

**S20 Table. Descriptive statistics of categorical features in the pulmonary disease group**

| **Variable** | **Values** | **Frequency** | **Percentage Frequency** |
| --- | --- | --- | --- |
| intubated | No | 24875 | 75.77 |
|  | Yes | 7956 | 24.23 |
| dialysis | No | 31655 | 96.42 |
|  | Yes | 1176 | 3.58 |
| gender | Male | 16945 | 51.61 |
|  | Female | 15880 | 48.37 |
|  | Unknown/Other | 4 | 0.01 |
|  | Missing | 2 | 0.01 |
| ethnicity | Caucasian | 25115 | 76.50 |
|  | African American | 3811 | 11.61 |
|  | Hispanic | 1682 | 5.12 |
|  | Other/Unknown | 1301 | 3.96 |
|  | Asian | 410 | 1.25 |
|  | Native American | 230 | 0.70 |
|  | Missing | 282 | 0.86 |
| unitstaytype | admit | 28587 | 87.07 |
|  | readmit | 2682 | 8.17 |
|  | transfer | 1562 | 4.76 |
| preopmi | No | 32772 | 99.82 |
|  | Yes | 59 | 0.18 |
| preopcardiaccath | No | 32723 | 99.67 |
|  | Yes | 108 | 0.33 |
| ptcawithin24h | No | 31786 | 96.82 |
|  | Yes | 1045 | 3.18 |
| thrombolytics | No | 32686 | 99.56 |
|  | Yes | 145 | 0.44 |
| aids | No | 32780 | 99.84 |
|  | Yes | 51 | 0.16 |
| hepaticfailure | No | 32360 | 98.57 |
|  | Yes | 471 | 1.43 |
| lymphoma | No | 32630 | 99.39 |
|  | Yes | 201 | 0.61 |
| immunosuppression | No | 31665 | 96.45 |
|  | Yes | 1166 | 3.55 |
| cirrhosis | No | 32234 | 98.18 |
|  | Yes | 597 | 1.82 |
| activetx | Yes | 25033 | 76.25 |
|  | No | 7798 | 23.75 |
| midur | No | 32574 | 99.22 |
|  | Yes | 257 | 0.78 |
| oobventday1 | No | 13686 | 41.69 |
|  | Yes | 19145 | 58.31 |
| oobintubday1 | No | 19435 | 59.20 |
|  | Yes | 13396 | 40.80 |
| diabetes | No | 25597 | 77.97 |
|  | Yes | 7234 | 22.03 |
| unitadmitsource | Emergency Department | 17015 | 51.83 |
|  | Floor | 6532 | 19.90 |
|  | Operating Room | 2674 | 8.14 |
|  | Direct Admit | 1879 | 5.72 |
|  | Recovery Room | 754 | 2.30 |
|  | Step-Down Unit (SDU) | 1358 | 4.14 |
|  | Acute Care/Floor | 1429 | 4.35 |
|  | Other Hospital | 792 | 2.41 |
|  | PACU | 157 | 0.48 |
|  | Other ICU | 142 | 0.43 |
|  | Chest Pain Center | 33 | 0.10 |
|  | ICU | 24 | 0.07 |
|  | ICU to SDU | 9 | 0.03 |
|  | Observation | 4 | 0.01 |
|  | Missing | 29 | 0.09 |
| ima | No | 32513 | 99.03 |
|  | Yes | 318 | 0.97 |
| meds | No | 32215 | 98.12 |
|  | Yes | 547 | 1.67 |
|  | Missing | 69 | 0.21 |
| ventday1 | No | 17870 | 54.43 |
|  | Yes | 14961 | 45.57 |
| unittype | Med-Surg ICU | 20080 | 61.16 |
|  | MICU | 3856 | 11.74 |
|  | Cardiac ICU | 2380 | 7.25 |
|  | SICU | 1958 | 5.96 |
|  | CCU-CTICU | 1910 | 5.82 |
|  | Neuro ICU | 862 | 2.63 |
|  | CTICU | 801 | 2.44 |
|  | CSICU | 984 | 3.00 |
| actualicumortality | Alive | 29702 | 90.47 |
|  | Expired | 3129 | 9.53 |
