# Supplementary material for: E-CatBoost: An efficient machine learning framework for predicting ICU mortality using the eICU Collaborative Research Database
Source: PLoS One. 2022 May 5;17(5):e0262895. doi: 10.1371/journal.pone.0262895 (PMC9070907; doi:10.1371/journal.pone.0262895)
Supplement: S21 Table — (DOCX) [file pone.0262895.s021.docx]

**S21 Table. Descriptive statistics of numerical features in the renal disease group**

| **Variable** | **Count** | **Mean** | **SD** | **Min.** | **Q_1_** | **Median** | **Q_3_** | **Max.** |
| --- | --- | --- | --- | --- | --- | --- | --- | --- |
| age | 18136 | 64.82 | 16.41 | 0.00 | 55.00 | 66.00 | 77.25 | 90.00 |
| admissionheight | 18136 | 168.81 | 11.82 | 57.90 | 160.00 | 168.90 | 177.80 | 213.40 |
| hospitaladmitoffset | 18136 | -2428.81 | 7664.27 | -230202.00 | -968.00 | -271.00 | -108.00 | 276.00 |
| admissionweight | 18136 | 84.19 | 27.58 | 0.40 | 65.70 | 79.40 | 97.83 | 349.20 |
| temperature | 18136 | 36.38 | 1.04 | 20.00 | 36.10 | 36.40 | 36.70 | 41.90 |
| respiratoryrate | 18136 | 24.91 | 14.18 | 4.00 | 11.00 | 27.00 | 35.00 | 60.00 |
| heartrate | 18136 | 102.99 | 30.78 | 20.00 | 90.00 | 106.00 | 123.00 | 220.00 |
| meanbp | 18136 | 82.58 | 43.21 | 40.00 | 49.00 | 61.00 | 122.00 | 200.00 |
| hematocrit | 18136 | 30.97 | 6.09 | 6.80 | 27.10 | 30.97 | 34.30 | 62.00 |
| verbal | 18136 | 3.96 | 1.50 | 1.00 | 4.00 | 5.00 | 5.00 | 5.00 |
| motor | 18136 | 5.45 | 1.25 | 1.00 | 6.00 | 6.00 | 6.00 | 6.00 |
| eyes | 18136 | 3.47 | 0.93 | 1.00 | 3.00 | 4.00 | 4.00 | 4.00 |
| potassium | 18136 | 4.24 | 0.72 | 1.17 | 3.77 | 4.20 | 4.60 | 8.60 |
| creatinine | 18136 | 2.71 | 2.52 | 0.12 | 1.10 | 1.95 | 3.20 | 37.35 |
| sodium | 18136 | 137.76 | 6.15 | 100.00 | 135.00 | 137.76 | 141.00 | 178.00 |
| BUN | 18136 | 40.40 | 27.77 | 1.00 | 20.00 | 36.00 | 52.00 | 263.33 |
| glucose | 18136 | 147.63 | 71.43 | 3.00 | 105.00 | 133.00 | 164.00 | 1478.00 |
| chloride | 18136 | 104.09 | 7.57 | 60.67 | 100.00 | 104.09 | 108.50 | 146.40 |
| calcium | 18136 | 8.21 | 0.85 | 3.94 | 7.75 | 8.21 | 8.70 | 18.60 |
| Hgb | 18136 | 10.41 | 1.98 | 3.00 | 9.00 | 10.41 | 11.60 | 19.50 |
| WBC x 1000 | 18136 | 12.96 | 10.33 | 0.00 | 8.11 | 11.90 | 14.75 | 444.14 |
| platelets x 1000 | 18136 | 197.03 | 92.31 | 3.00 | 140.00 | 197.03 | 232.50 | 1219.00 |
| RBC | 18136 | 3.52 | 0.68 | 0.98 | 3.06 | 3.52 | 3.91 | 8.00 |
| bicarbonate | 18136 | 22.84 | 5.04 | 4.00 | 20.00 | 22.84 | 25.75 | 55.00 |
| MCV | 18136 | 90.43 | 6.77 | 55.00 | 87.00 | 90.43 | 94.00 | 137.95 |
| MCHC | 18136 | 32.91 | 1.38 | 25.25 | 32.20 | 32.91 | 33.70 | 40.60 |
| MCH | 18136 | 29.75 | 2.40 | 17.00 | 28.80 | 29.75 | 31.00 | 44.10 |
| RDW | 18136 | 15.88 | 2.29 | 10.80 | 14.40 | 15.88 | 16.57 | 33.05 |
