# Supplementary material for: E-CatBoost: An efficient machine learning framework for predicting ICU mortality using the eICU Collaborative Research Database
Source: PLoS One. 2022 May 5;17(5):e0262895. doi: 10.1371/journal.pone.0262895 (PMC9070907; doi:10.1371/journal.pone.0262895)
Supplement: S22 Table — (DOCX) [file pone.0262895.s022.docx]

**S22 Table. Descriptive statistics of categorical features in the renal disease group**

| **Variable** | **Values** | **Frequency** | **Percentage Frequency** |
| --- | --- | --- | --- |
| intubated | No | 15267 | 84.18 |
|  | Yes | 2869 | 15.82 |
| dialysis | No | 16227 | 89.47 |
|  | Yes | 1909 | 10.53 |
| gender | Male | 9494 | 52.35 |
|  | Female | 8636 | 47.62 |
|  | Unknown/Other | 4 | 0.02 |
|  | Missing | 2 | 0.01 |
| ethnicity | Caucasian | 13264 | 73.14 |
|  | African American | 2491 | 13.74 |
|  | Hispanic | 1165 | 6.42 |
|  | Other/Unknown | 748 | 4.12 |
|  | Asian | 226 | 1.25 |
|  | Native American | 126 | 0.69 |
|  | Missing | 116 | 0.64 |
| unitstaytype | admit | 16235 | 89.52 |
|  | readmit | 1224 | 6.75 |
|  | transfer | 677 | 3.73 |
| preopmi | No | 18121 | 99.92 |
|  | Yes | 15 | 0.08 |
| preopcardiaccath | No | 18104 | 99.82 |
|  | Yes | 32 | 0.18 |
| ptcawithin24h | No | 17402 | 95.95 |
|  | Yes | 734 | 4.05 |
| thrombolytics | No | 18025 | 99.39 |
|  | Yes | 111 | 0.61 |
| aids | No | 18114 | 99.88 |
|  | Yes | 22 | 0.12 |
| hepaticfailure | No | 17774 | 98.00 |
|  | Yes | 362 | 2.00 |
| lymphoma | No | 18030 | 99.42 |
|  | Yes | 106 | 0.58 |
| immunosuppression | No | 17591 | 96.99 |
|  | Yes | 545 | 3.01 |
| cirrhosis | No | 17683 | 97.50 |
|  | Yes | 453 | 2.50 |
| activetx | Yes | 12548 | 69.19 |
|  | No | 5588 | 30.81 |
| midur | No | 17958 | 99.02 |
|  | Yes | 178 | 0.98 |
| oobventday1 | No | 12238 | 67.48 |
|  | Yes | 5898 | 32.52 |
| oobintubday1 | No | 13757 | 75.85 |
|  | Yes | 4379 | 24.15 |
| diabetes | No | 12926 | 71.27 |
|  | Yes | 5210 | 28.73 |
| unitadmitsource | Emergency Department | 10543 | 58.13 |
|  | Floor | 3316 | 18.28 |
|  | Operating Room | 1186 | 6.54 |
|  | Direct Admit | 1038 | 5.72 |
|  | Recovery Room | 473 | 2.61 |
|  | Step-Down Unit (SDU) | 564 | 3.11 |
|  | Acute Care/Floor | 466 | 2.57 |
|  | Other Hospital | 363 | 2.00 |
|  | PACU | 59 | 0.33 |
|  | Other ICU | 74 | 0.41 |
|  | Chest Pain Center | 23 | 0.13 |
|  | ICU | 12 | 0.07 |
|  | ICU to SDU | 2 | 0.01 |
|  | Missing | 17 | 0.09 |
| ima | No | 17958 | 99.02 |
|  | Yes | 178 | 0.98 |
| meds | No | 17898 | 98.69 |
|  | Yes | 208 | 1.15 |
|  | Missing | 30 | 0.17 |
| ventday1 | No | 13740 | 75.76 |
|  | Yes | 4396 | 24.24 |
| unittype | Med-Surg ICU | 11295 | 62.28 |
|  | MICU | 1938 | 10.69 |
|  | Cardiac ICU | 1529 | 8.43 |
|  | SICU | 1359 | 7.49 |
|  | CCU-CTICU | 1142 | 6.30 |
|  | Neuro ICU | 282 | 1.55 |
|  | CTICU | 361 | 1.99 |
|  | CSICU | 230 | 1.27 |
| actualicumortality | Alive | 16601 | 91.54 |
|  | Expired | 1535 | 8.46 |
