# Supplementary material for: E-CatBoost: An efficient machine learning framework for predicting ICU mortality using the eICU Collaborative Research Database
Source: PLoS One. 2022 May 5;17(5):e0262895. doi: 10.1371/journal.pone.0262895 (PMC9070907; doi:10.1371/journal.pone.0262895)
Supplement: S23 Table — (DOCX) [file pone.0262895.s023.docx]

**S23 Table. Descriptive statistics of numerical features in the surgery disease group**

| **Variable** | **Count** | **Mean** | **SD** | **Min.** | **Q_1_** | **Median** | **Q_3_** | **Max.** |
| --- | --- | --- | --- | --- | --- | --- | --- | --- |
| age | 2655 | 59.95 | 18.64 | 14.00 | 47.00 | 62.00 | 75.00 | 90.00 |
| admissionheight | 2655 | 167.89 | 11.20 | 104.10 | 160.00 | 167.60 | 175.30 | 203.20 |
| hospitaladmitoffset | 2655 | -3209.52 | 6968.48 | -73751.00 | -2869.50 | -718.00 | -280.50 | 124.00 |
| admissionweight | 2655 | 84.54 | 27.49 | 14.40 | 66.10 | 80.30 | 97.50 | 349.20 |
| temperature | 2655 | 36.47 | 0.89 | 23.30 | 36.20 | 36.50 | 36.80 | 40.90 |
| respiratoryrate | 2655 | 21.77 | 14.40 | 4.00 | 9.00 | 16.00 | 33.00 | 60.00 |
| heartrate | 2655 | 103.72 | 29.30 | 20.00 | 92.00 | 107.00 | 122.00 | 212.00 |
| meanbp | 2655 | 84.15 | 42.03 | 40.00 | 53.00 | 64.00 | 120.00 | 200.00 |
| hematocrit | 2655 | 29.86 | 5.99 | 7.50 | 26.00 | 29.86 | 33.45 | 52.00 |
| verbal | 2655 | 3.96 | 1.59 | 1.00 | 3.00 | 5.00 | 5.00 | 5.00 |
| motor | 2655 | 5.55 | 1.19 | 1.00 | 6.00 | 6.00 | 6.00 | 6.00 |
| eyes | 2655 | 3.51 | 0.89 | 1.00 | 3.00 | 4.00 | 4.00 | 4.00 |
| potassium | 2655 | 4.15 | 0.53 | 2.20 | 3.80 | 4.15 | 4.40 | 7.60 |
| creatinine | 2655 | 1.26 | 1.22 | 0.12 | 0.70 | 0.95 | 1.26 | 16.89 |
| sodium | 2655 | 138.21 | 4.12 | 110.43 | 136.00 | 138.21 | 140.50 | 170.00 |
| BUN | 2655 | 20.61 | 15.57 | 1.00 | 11.00 | 17.00 | 23.10 | 171.00 |
| glucose | 2655 | 142.52 | 47.02 | 31.00 | 112.00 | 139.00 | 159.00 | 589.50 |
| chloride | 2655 | 105.31 | 5.16 | 78.00 | 102.33 | 105.31 | 108.00 | 133.50 |
| calcium | 2655 | 8.03 | 0.70 | 5.15 | 7.60 | 8.03 | 8.45 | 11.95 |
| Hgb | 2655 | 10.25 | 1.89 | 3.65 | 8.90 | 10.25 | 11.40 | 17.35 |
| WBC x 1000 | 2655 | 12.75 | 6.32 | 0.15 | 9.05 | 12.25 | 14.66 | 99.27 |
| platelets x 1000 | 2655 | 205.68 | 90.69 | 20.00 | 148.00 | 203.00 | 241.00 | 1034.00 |
| RBC | 2655 | 3.47 | 0.64 | 1.48 | 3.04 | 3.47 | 3.83 | 5.79 |
| bicarbonate | 2655 | 23.85 | 3.99 | 7.00 | 22.00 | 23.85 | 26.00 | 45.00 |
| MCV | 2655 | 89.51 | 6.15 | 62.50 | 86.45 | 89.51 | 93.00 | 123.60 |
| MCHC | 2655 | 33.12 | 1.28 | 27.20 | 32.45 | 33.12 | 33.95 | 38.00 |
| MCH | 2655 | 29.65 | 2.20 | 18.43 | 28.86 | 29.65 | 30.80 | 42.30 |
| RDW | 2655 | 15.20 | 2.09 | 11.40 | 13.90 | 15.20 | 15.70 | 31.15 |
| anion gap | 2655 | 9.73 | 3.17 | 1.00 | 8.00 | 9.73 | 11.00 | 31.50 |
