# Supplementary material for: E-CatBoost: An efficient machine learning framework for predicting ICU mortality using the eICU Collaborative Research Database
Source: PLoS One. 2022 May 5;17(5):e0262895. doi: 10.1371/journal.pone.0262895 (PMC9070907; doi:10.1371/journal.pone.0262895)
Supplement: S24 Table — (DOCX) [file pone.0262895.s024.docx]

**S24 Table. Descriptive statistics of categorical features in the surgery disease group**

| **Variable** | **Values** | **Frequency** | **Percentage Frequency** |
| --- | --- | --- | --- |
| intubated | No | 2159 | 81.32 |
|  | Yes | 496 | 18.68 |
| dialysis | No | 2576 | 97.02 |
|  | Yes | 79 | 2.98 |
| gender | Male | 1476 | 55.59 |
|  | Female | 1179 | 44.41 |
| ethnicity | Caucasian | 1981 | 74.61 |
|  | African American | 274 | 10.32 |
|  | Hispanic | 209 | 7.87 |
|  | Other/Unknown | 116 | 4.37 |
|  | Asian | 42 | 1.58 |
|  | Native American | 18 | 0.68 |
|  | Missing | 15 | 0.56 |
| unitstaytype | admit | 2368 | 89.19 |
|  | readmit | 187 | 7.04 |
|  | transfer | 100 | 3.77 |
| preopmi | No | 2645 | 99.62 |
|  | Yes | 10 | 0.38 |
| preopcardiaccath | No | 2641 | 99.47 |
|  | Yes | 14 | 0.53 |
| ptcawithin24h | No | 2602 | 98.00 |
|  | Yes | 53 | 2.00 |
| thrombolytics | No | 2645 | 99.62 |
|  | Yes | 10 | 0.38 |
| aids | No | 2654 | 99.96 |
|  | Yes | 1 | 0.04 |
| hepaticfailure | No | 2635 | 99.25 |
|  | Yes | 20 | 0.75 |
| lymphoma | No | 2649 | 99.77 |
|  | Yes | 6 | 0.23 |
| immunosuppression | No | 2603 | 98.04 |
|  | Yes | 52 | 1.96 |
| cirrhosis | No | 2626 | 98.91 |
|  | Yes | 29 | 1.09 |
| activetx | Yes | 1635 | 61.58 |
|  | No | 1020 | 38.42 |
| midur | No | 2633 | 99.17 |
|  | Yes | 22 | 0.83 |
| oobventday1 | No | 1625 | 61.21 |
|  | Yes | 1030 | 38.79 |
| oobintubday1 | No | 1740 | 65.54 |
|  | Yes | 915 | 34.46 |
| diabetes | No | 2168 | 81.66 |
|  | Yes | 487 | 18.34 |
| unitadmitsource | Emergency Department | 389 | 14.65 |
|  | Floor | 373 | 14.05 |
|  | Operating Room | 1084 | 40.83 |
|  | Direct Admit | 83 | 3.13 |
|  | Recovery Room | 427 | 16.08 |
|  | Step-Down Unit (SDU) | 53 | 2.00 |
|  | Acute Care/Floor | 61 | 2.30 |
|  | Other Hospital | 39 | 1.47 |
|  | PACU | 132 | 4.97 |
|  | Other ICU | 7 | 0.26 |
|  | ICU | 2 | 0.08 |
|  | ICU to SDU | 2 | 0.08 |
|  | Other | 1 | 0.04 |
|  | Missing | 2 | 0.08 |
| ima | No | 2594 | 97.70 |
|  | Yes | 61 | 2.30 |
| meds | No | 2615 | 98.49 |
|  | Yes | 38 | 1.43 |
|  | Missing | 2 | 0.08 |
| ventday1 | No | 1892 | 71.26 |
|  | Yes | 763 | 28.74 |
| unittype | Med-Surg ICU | 1571 | 59.17 |
|  | MICU | 145 | 5.46 |
|  | Cardiac ICU | 118 | 4.44 |
|  | SICU | 401 | 15.10 |
|  | CCU-CTICU | 111 | 4.18 |
|  | Neuro ICU | 119 | 4.48 |
|  | CTICU | 113 | 4.26 |
|  | CSICU | 77 | 2.90 |
| actualicumortality | Alive | 2543 | 95.78 |
|  | Expired | 112 | 4.22 |
