# Supplementary material for: E-CatBoost: An efficient machine learning framework for predicting ICU mortality using the eICU Collaborative Research Database
Source: PLoS One. 2022 May 5;17(5):e0262895. doi: 10.1371/journal.pone.0262895 (PMC9070907; doi:10.1371/journal.pone.0262895)
Supplement: S25 Table — (DOCX) [file pone.0262895.s025.docx]

**S25 Table. Descriptive statistics of numerical features in the toxicology disease group**

| **Variable** | **Count** | **Mean** | **SD** | **Min.** | **Q_1_** | **Median** | **Q_3_** | **Max.** |
| --- | --- | --- | --- | --- | --- | --- | --- | --- |
| age | 4153 | 43.84 | 15.88 | 0.00 | 31.00 | 44.00 | 55.00 | 90.00 |
| admissionheight | 4153 | 170.80 | 10.82 | 63.50 | 164.00 | 170.20 | 177.80 | 208.30 |
| hospitaladmitoffset | 4153 | -515.52 | 2262.16 | -51454.00 | -289.00 | -147.00 | -48.00 | 342.00 |
| admissionweight | 4153 | 81.19 | 22.76 | 0.50 | 65.70 | 77.70 | 92.98 | 214.20 |
| temperature | 4153 | 36.45 | 0.87 | 21.70 | 36.20 | 36.50 | 36.72 | 41.00 |
| respiratoryrate | 4153 | 23.25 | 14.57 | 4.00 | 10.00 | 24.00 | 33.00 | 60.00 |
| heartrate | 4153 | 102.49 | 30.02 | 20.00 | 92.00 | 107.00 | 122.00 | 215.00 |
| meanbp | 4153 | 87.29 | 37.77 | 40.00 | 56.00 | 69.00 | 120.00 | 200.00 |
| hematocrit | 4153 | 36.34 | 4.80 | 16.50 | 34.70 | 36.34 | 38.50 | 62.00 |
| verbal | 4153 | 3.54 | 1.64 | 1.00 | 2.00 | 4.00 | 5.00 | 5.00 |
| motor | 4153 | 5.14 | 1.50 | 1.00 | 5.00 | 6.00 | 6.00 | 6.00 |
| eyes | 4153 | 3.10 | 1.09 | 1.00 | 2.00 | 3.00 | 4.00 | 4.00 |
| potassium | 4153 | 3.86 | 0.45 | 2.10 | 3.60 | 3.86 | 4.07 | 6.46 |
| creatinine | 4153 | 1.02 | 0.88 | 0.14 | 0.68 | 0.85 | 1.02 | 17.82 |
| sodium | 4153 | 139.69 | 4.02 | 111.33 | 138.00 | 139.69 | 142.00 | 177.33 |
| BUN | 4153 | 14.07 | 11.67 | 1.00 | 8.00 | 12.00 | 14.50 | 204.75 |
| glucose | 4153 | 117.36 | 43.30 | 32.00 | 93.00 | 111.00 | 122.25 | 683.29 |
| chloride | 4153 | 106.34 | 5.23 | 68.50 | 104.00 | 106.34 | 109.00 | 142.50 |
| calcium | 4153 | 8.23 | 0.61 | 5.10 | 7.90 | 8.23 | 8.60 | 11.84 |
| Hgb | 4153 | 12.25 | 1.67 | 6.30 | 11.50 | 12.25 | 13.10 | 19.40 |
| WBC x 1000 | 4153 | 9.88 | 4.34 | 0.70 | 7.20 | 9.88 | 10.70 | 51.80 |
| platelets x 1000 | 4153 | 205.29 | 68.69 | 15.00 | 171.00 | 205.29 | 232.00 | 827.00 |
| RBC | 4153 | 4.03 | 0.54 | 1.83 | 3.81 | 4.03 | 4.29 | 6.47 |
| bicarbonate | 4153 | 23.93 | 3.58 | 7.25 | 22.00 | 23.93 | 26.00 | 48.00 |
| MCV | 4153 | 91.32 | 5.58 | 59.00 | 89.00 | 91.32 | 93.45 | 123.90 |
| MCHC | 4153 | 33.40 | 1.15 | 27.60 | 33.00 | 33.40 | 34.00 | 38.65 |
