# Supplementary material for: E-CatBoost: An efficient machine learning framework for predicting ICU mortality using the eICU Collaborative Research Database
Source: PLoS One. 2022 May 5;17(5):e0262895. doi: 10.1371/journal.pone.0262895 (PMC9070907; doi:10.1371/journal.pone.0262895)
Supplement: S26 Table — (DOCX) [file pone.0262895.s026.docx]

**S26 Table. Descriptive statistics of categorical features in the toxicology disease group**

| **Variable** | **Values** | **Frequency** | **Percentage Frequency** |
| --- | --- | --- | --- |
| intubated | No | 3477 | 83.72 |
|  | Yes | 676 | 16.28 |
| dialysis | No | 4140 | 99.69 |
|  | Yes | 13 | 0.31 |
| gender | Male | 2096 | 50.47 |
|  | Female | 2052 | 49.41 |
|  | Unknown/Other | 5 | 0.12 |
| ethnicity | Caucasian | 3323 | 80.01 |
|  | African American | 313 | 7.54 |
|  | Hispanic | 133 | 3.20 |
|  | Other/Unknown | 182 | 4.38 |
|  | Asian | 39 | 0.94 |
|  | Native American | 70 | 1.69 |
|  | Missing | 93 | 2.24 |
| unitstaytype | admit | 3877 | 93.35 |
|  | readmit | 207 | 4.98 |
|  | transfer | 69 | 1.66 |
| preopmi | No | 4153 | 100.00 |
|  | Yes | 0 | 0.00 |
| preopcardiaccath | No | 4152 | 99.98 |
|  | Yes | 1 | 0.02 |
| ptcawithin24h | No | 4108 | 98.92 |
|  | Yes | 45 | 1.08 |
| thrombolytics | No | 4148 | 99.88 |
|  | Yes | 5 | 0.12 |
| aids | No | 4151 | 99.95 |
|  | Yes | 2 | 0.05 |
| hepaticfailure | No | 4100 | 98.72 |
|  | Yes | 53 | 1.28 |
| lymphoma | No | 4147 | 99.86 |
|  | Yes | 6 | 0.14 |
| immunosuppression | No | 4141 | 99.71 |
|  | Yes | 12 | 0.29 |
| cirrhosis | No | 4083 | 98.31 |
|  | Yes | 70 | 1.69 |
| activetx | Yes | 1988 | 47.87 |
|  | No | 2165 | 52.13 |
| midur | No | 4144 | 99.78 |
|  | Yes | 9 | 0.22 |
| oobventday1 | No | 2791 | 67.20 |
|  | Yes | 1362 | 32.80 |
| oobintubday1 | No | 2896 | 69.73 |
|  | Yes | 1257 | 30.27 |
| diabetes | No | 3812 | 91.79 |
|  | Yes | 341 | 8.21 |
| unitadmitsource | Emergency Department | 3463 | 83.39 |
|  | Floor | 209 | 5.03 |
|  | Operating Room | 20 | 0.48 |
|  | Direct Admit | 231 | 5.56 |
|  | Recovery Room | 9 | 0.22 |
|  | Step-Down Unit (SDU) | 60 | 1.44 |
|  | Acute Care/Floor | 79 | 1.90 |
|  | Other Hospital | 61 | 1.47 |
|  | Other ICU | 16 | 0.39 |
|  | ICU | 4 | 0.10 |
|  | Missing | 1 | 0.02 |
| ima | No | 4152 | 99.98 |
|  | Yes | 1 | 0.02 |
| meds | No | 4094 | 98.58 |
|  | Yes | 51 | 1.23 |
|  | Missing | 8 | 0.19 |
| ventday1 | No | 3135 | 75.49 |
|  | Yes | 1018 | 24.51 |
| unittype | Med-Surg ICU | 3028 | 72.91 |
|  | MICU | 441 | 10.62 |
|  | Cardiac ICU | 222 | 5.35 |
|  | SICU | 90 | 2.17 |
|  | CCU-CTICU | 202 | 4.86 |
|  | Neuro ICU | 105 | 2.53 |
|  | CTICU | 13 | 0.31 |
|  | CSICU | 52 | 1.25 |
| actualicumortality | Alive | 4094 | 98.58 |
|  | Expired | 59 | 1.42 |
