# Supplementary material for: E-CatBoost: An efficient machine learning framework for predicting ICU mortality using the eICU Collaborative Research Database
Source: PLoS One. 2022 May 5;17(5):e0262895. doi: 10.1371/journal.pone.0262895 (PMC9070907; doi:10.1371/journal.pone.0262895)
Supplement: S27 Table — (DOCX) [file pone.0262895.s027.docx]

**S27 Table. The required computation time (seconds) for training and testing E-CatBoost and CatBoost models**

| **Models**  **Disease group** | **Hold-out set (train:80%-test:20%)** | | **10-fold CV** | |
| --- | --- | --- | --- | --- |
|  | **CatBoost** | **E-CatBoost** | **CatBoost** | **E-CatBoost** |
| **burns/trauma** | 13.54 | 6.53 | 178.22 | 45.16 |
| **cardiovascular** | 85.41 | 39.05 | 1215.64 | 472.76 |
| **endocrine** | 15.77 | 14.70 | 247.62 | 185.55 |
| **gastrointestinal** | 20.04 | 23.95 | 465.50 | 295.06 |
| **hematology** | 10.45 | 4.64 | 168.36 | 293.06 |
| **infectious diseases** | 17.18 | 5.73 | 511.43 | 315.05 |
| **neurologic** | 42.09 | 31.56 | 963.29 | 383.15 |
| **oncology** | 26.52 | 5.35 | 245.94 | 135.42 |
| **pulmonary** | 41.09 | 28.52 | 504.80 | 564.20 |
| **renal** | 34.54 | 25.77 | 862.90 | 211.59 |
| **surgery** | 25.87 | 6.19 | 380.53 | 188.67 |
| **toxicology** | 25.14 | 16.63 | 87.62 | 57.07 |
