# Supplementary figures and images for: E-CatBoost: An efficient machine learning framework for predicting ICU mortality using the eICU Collaborative Research Database
Source: PLoS One. 2022 May 5;17(5):e0262895. doi: 10.1371/journal.pone.0262895 (PMC9070907; doi:10.1371/journal.pone.0262895)

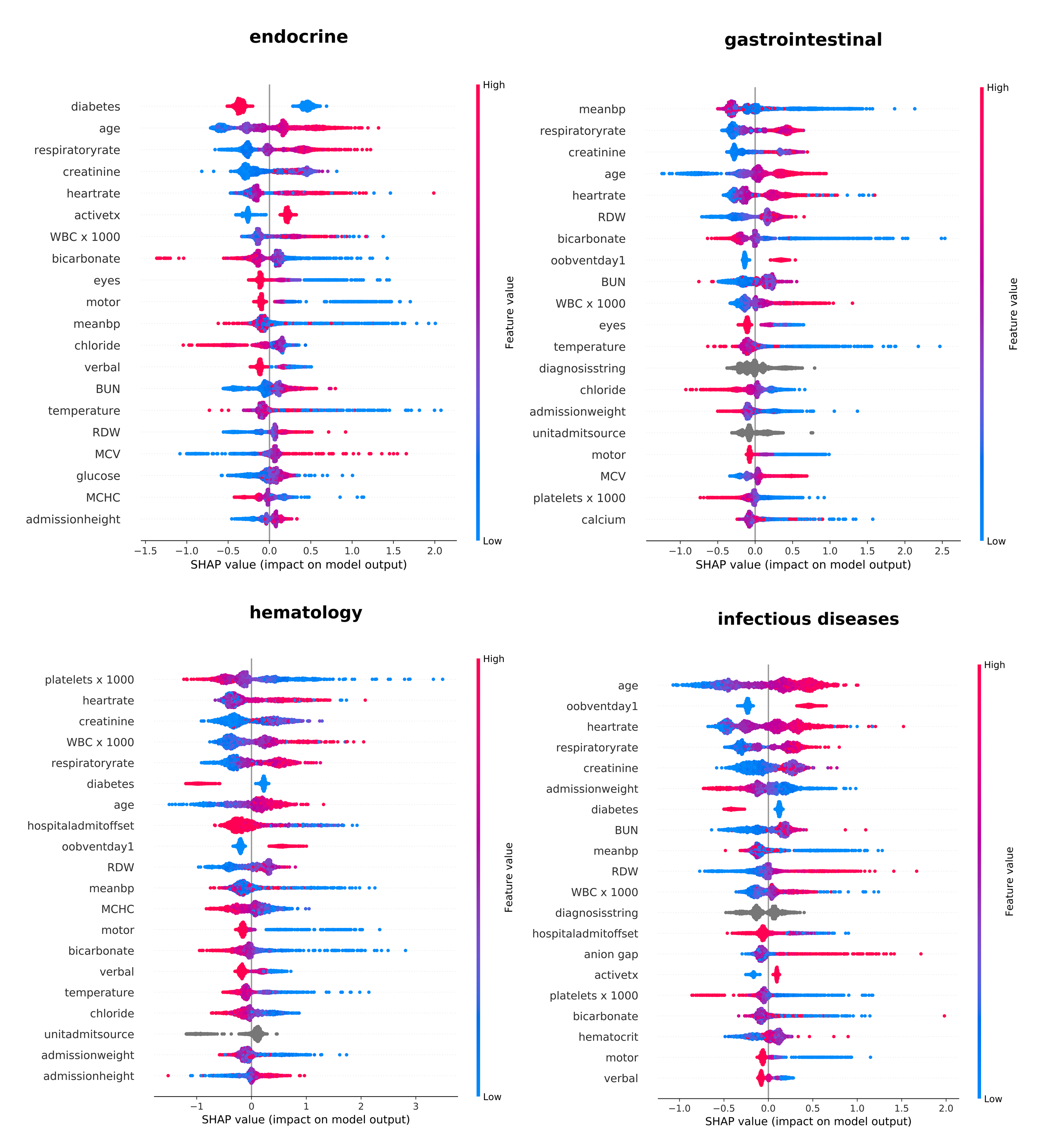

Supplement: S1 Fig — (TIF) [file pone.0262895.s028.tif]

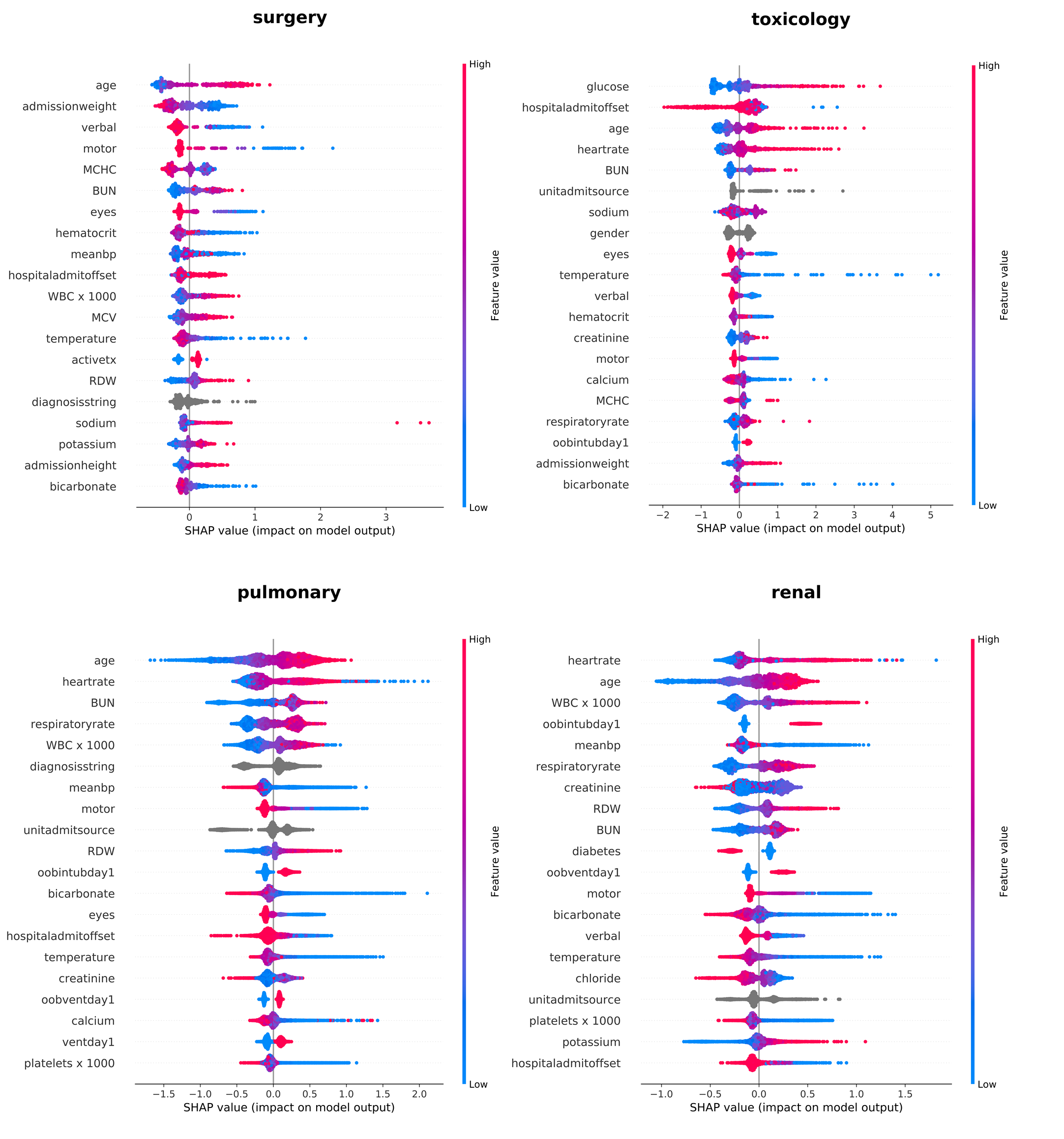

Supplement: S2 Fig — (TIF) [file pone.0262895.s029.tif]

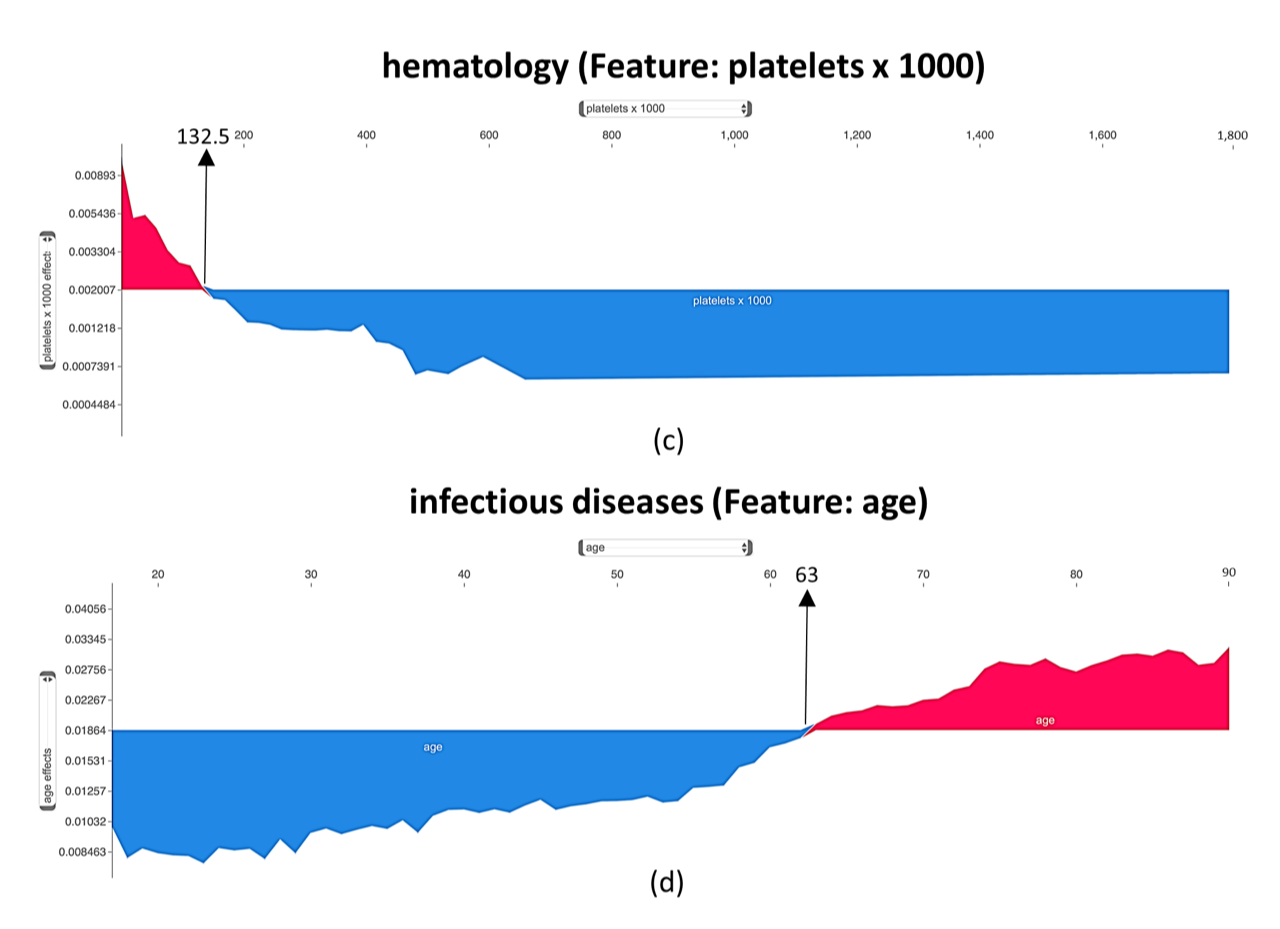

Supplement: S3 Fig — (TIF) [file pone.0262895.s030.tif]

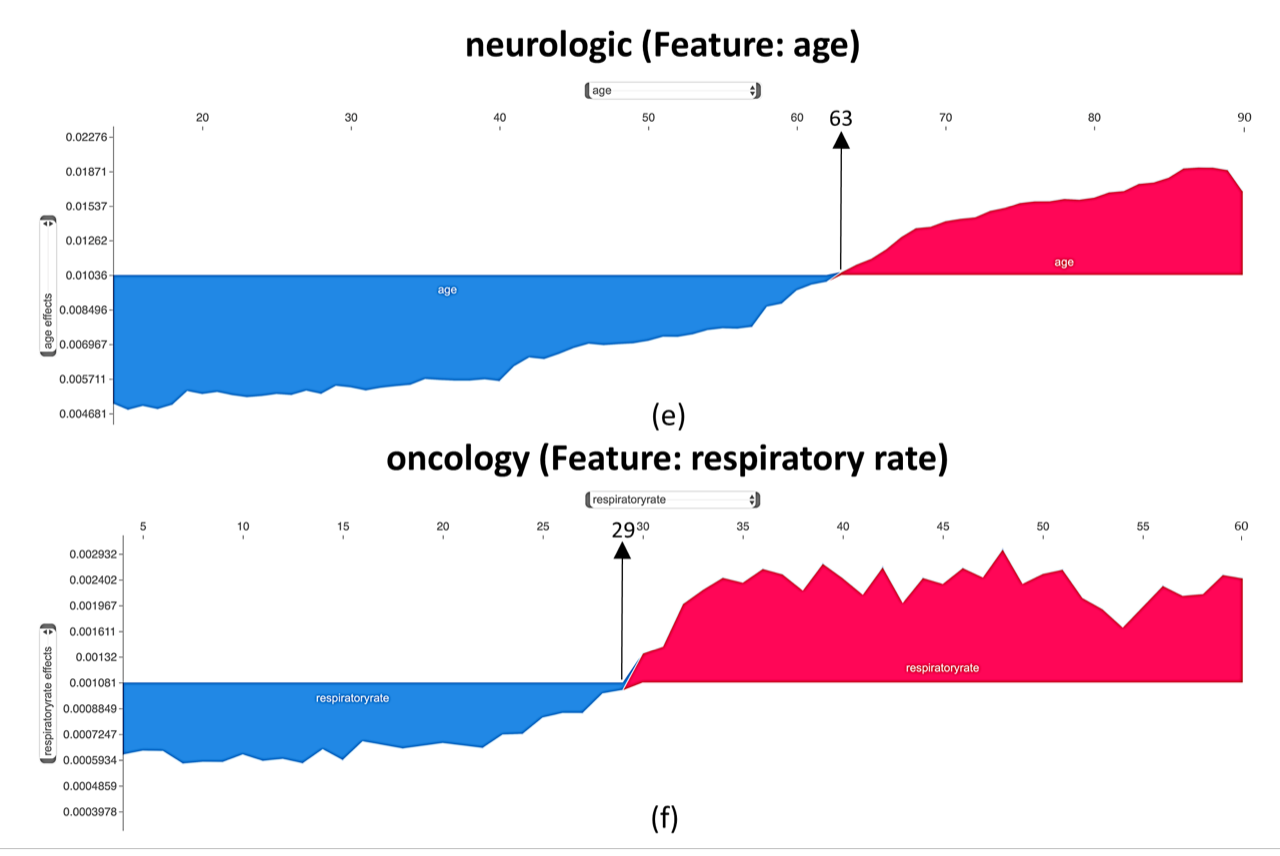

Supplement: S4 Fig — (TIF) [file pone.0262895.s031.tif]

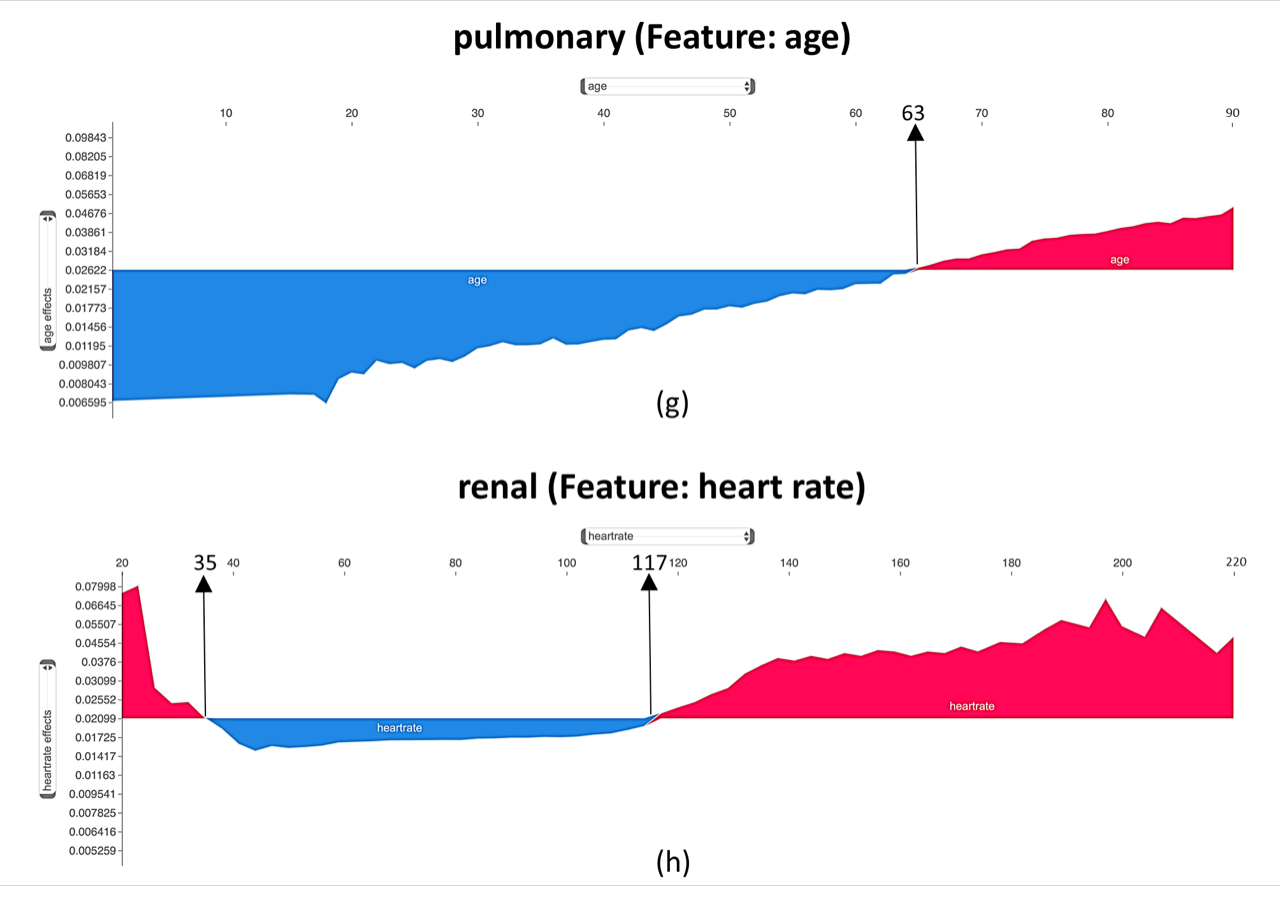

Supplement: S5 Fig — (TIF) [file pone.0262895.s032.tif]

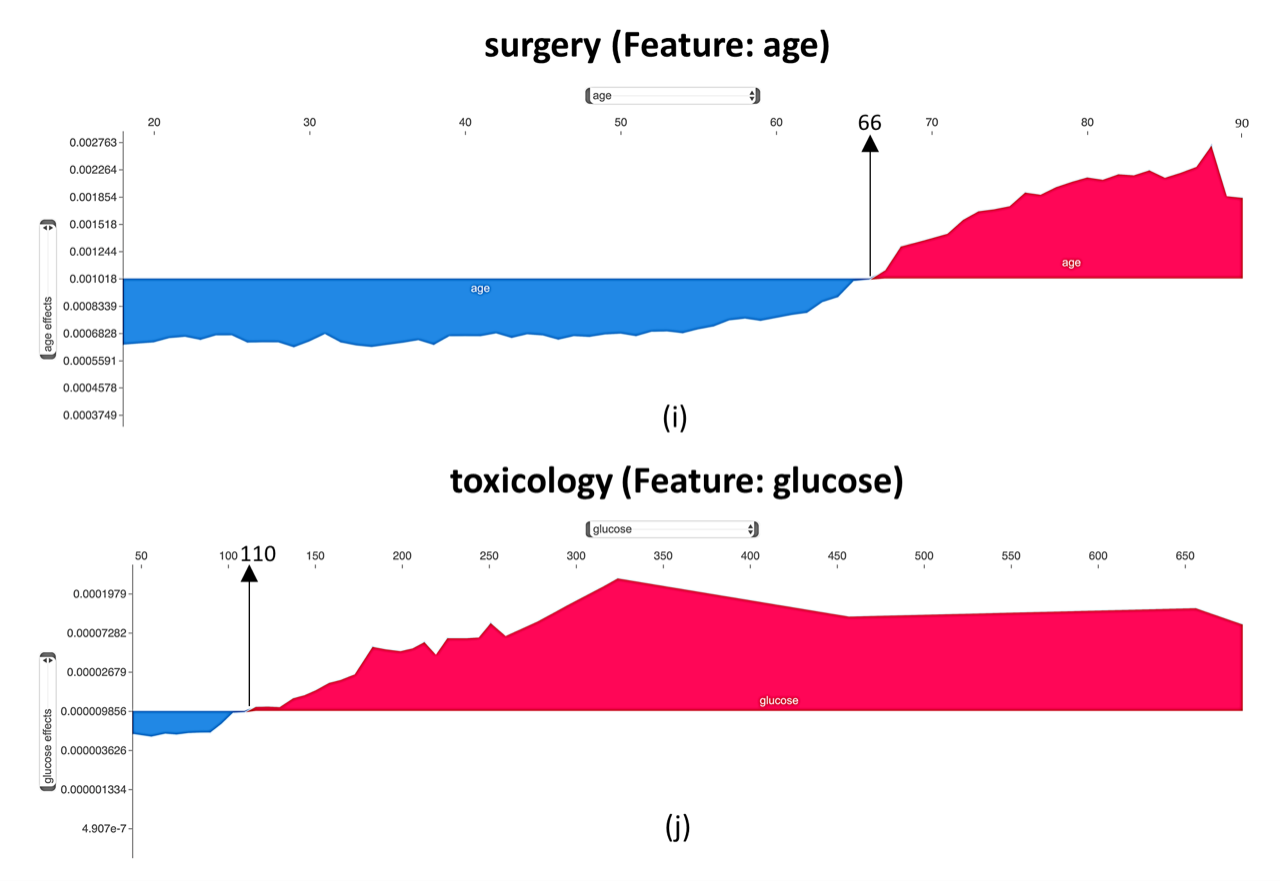

Supplement: S6 Fig — (TIF) [file pone.0262895.s033.tif]

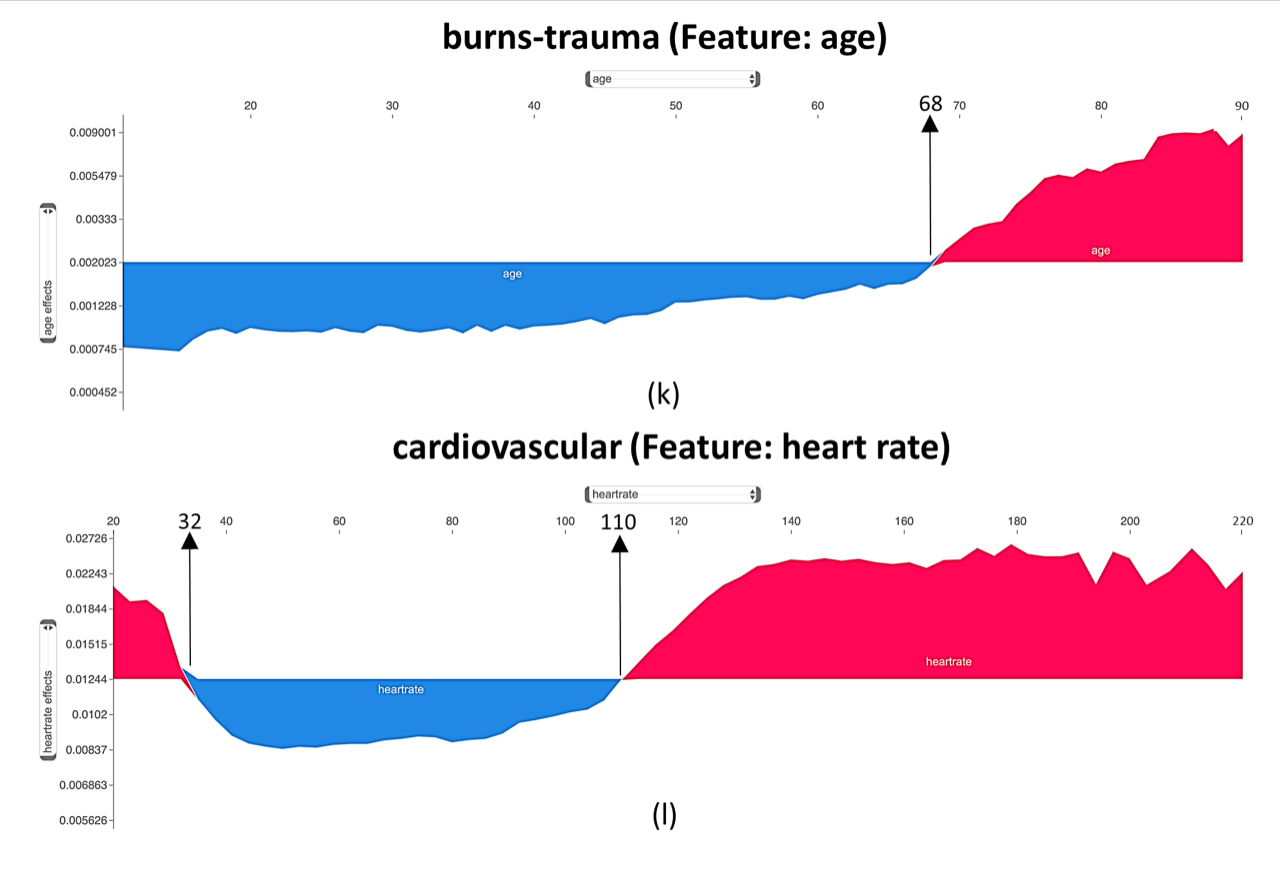

Supplement: S7 Fig — (TIF) [file pone.0262895.s034.tif]

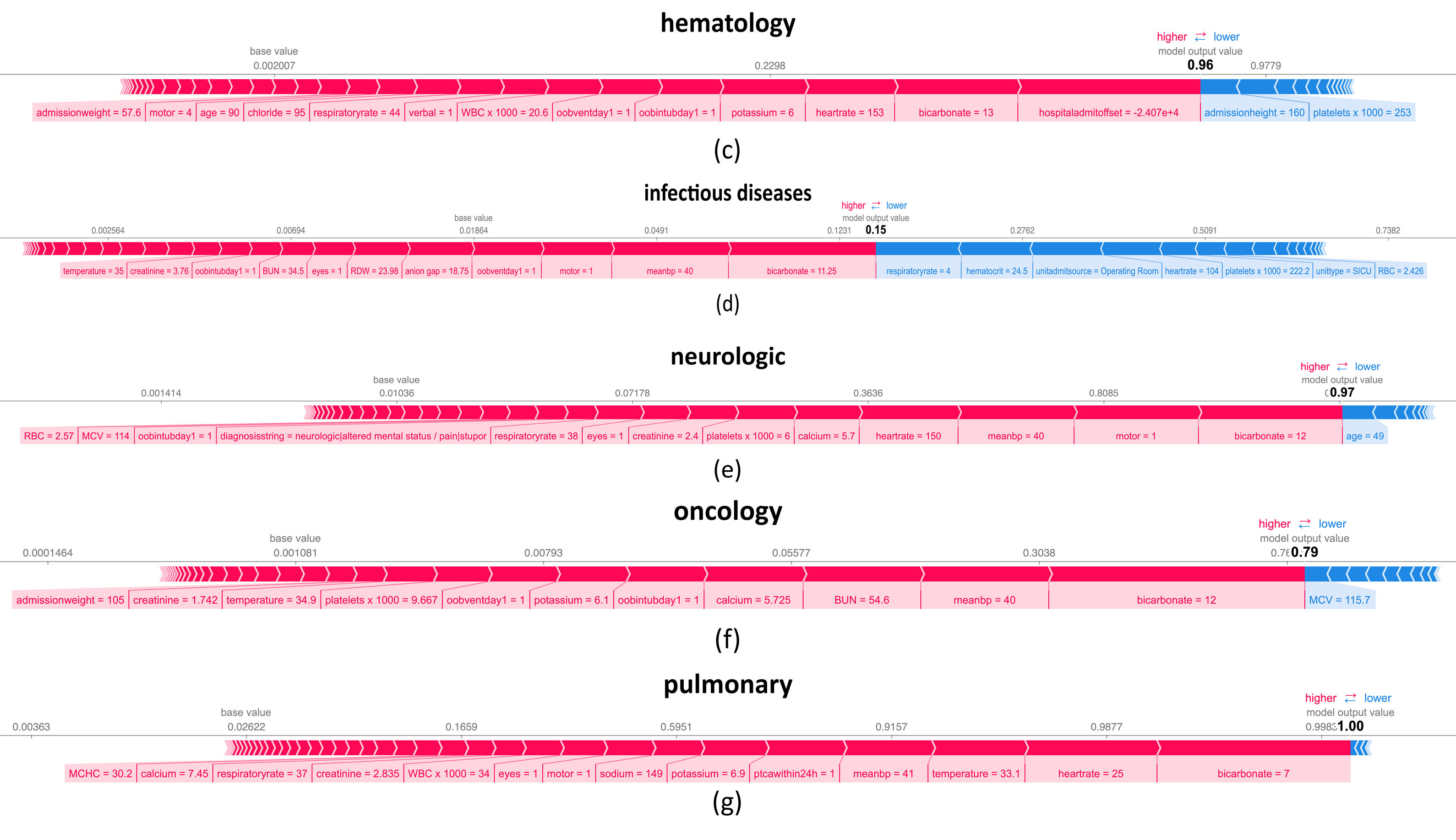

Supplement: S8 Fig — (TIF) [file pone.0262895.s035.tif]

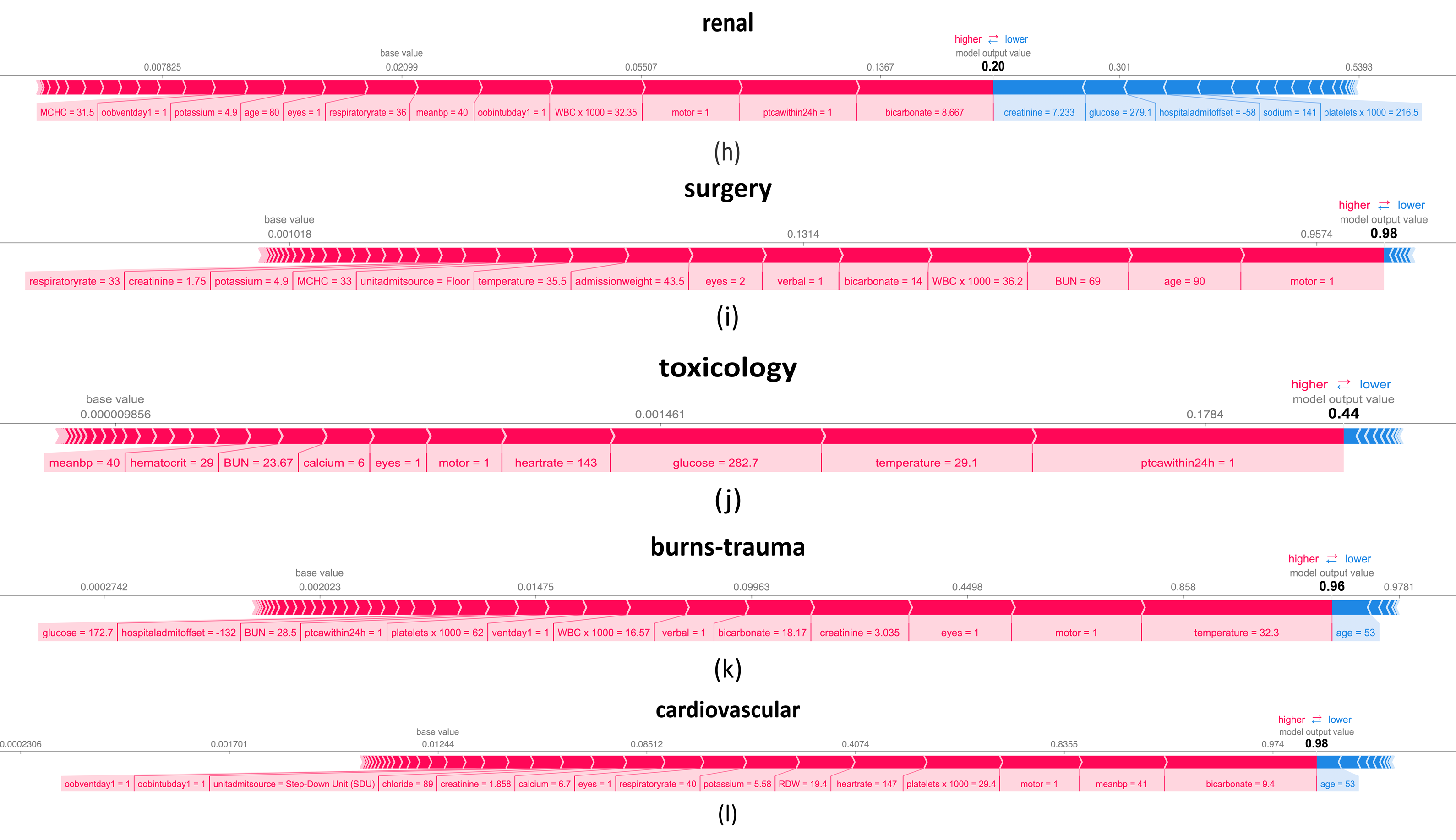

Supplement: S9 Fig — (TIF) [file pone.0262895.s036.tif]

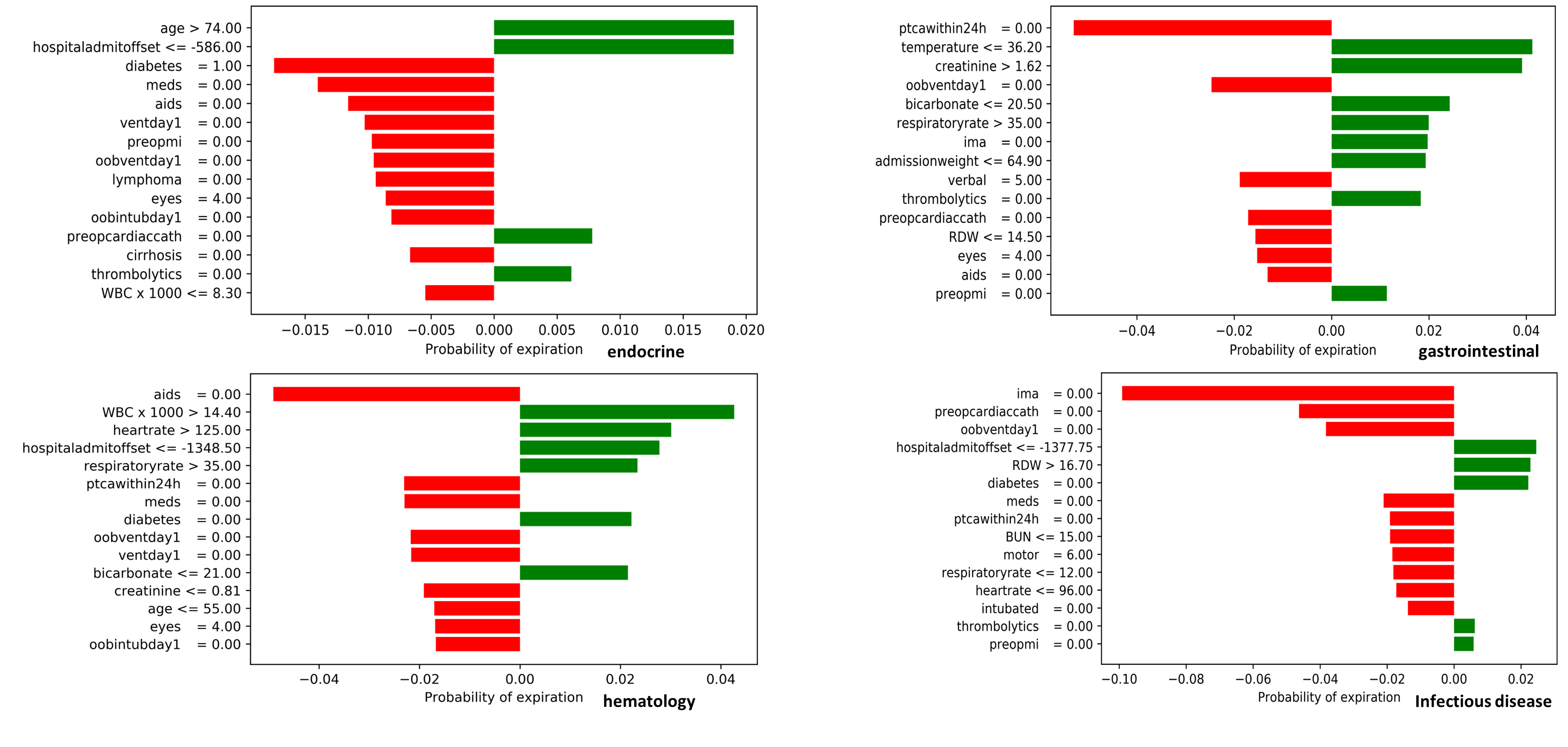

Supplement: S10 Fig — (TIF) [file pone.0262895.s037.tif]

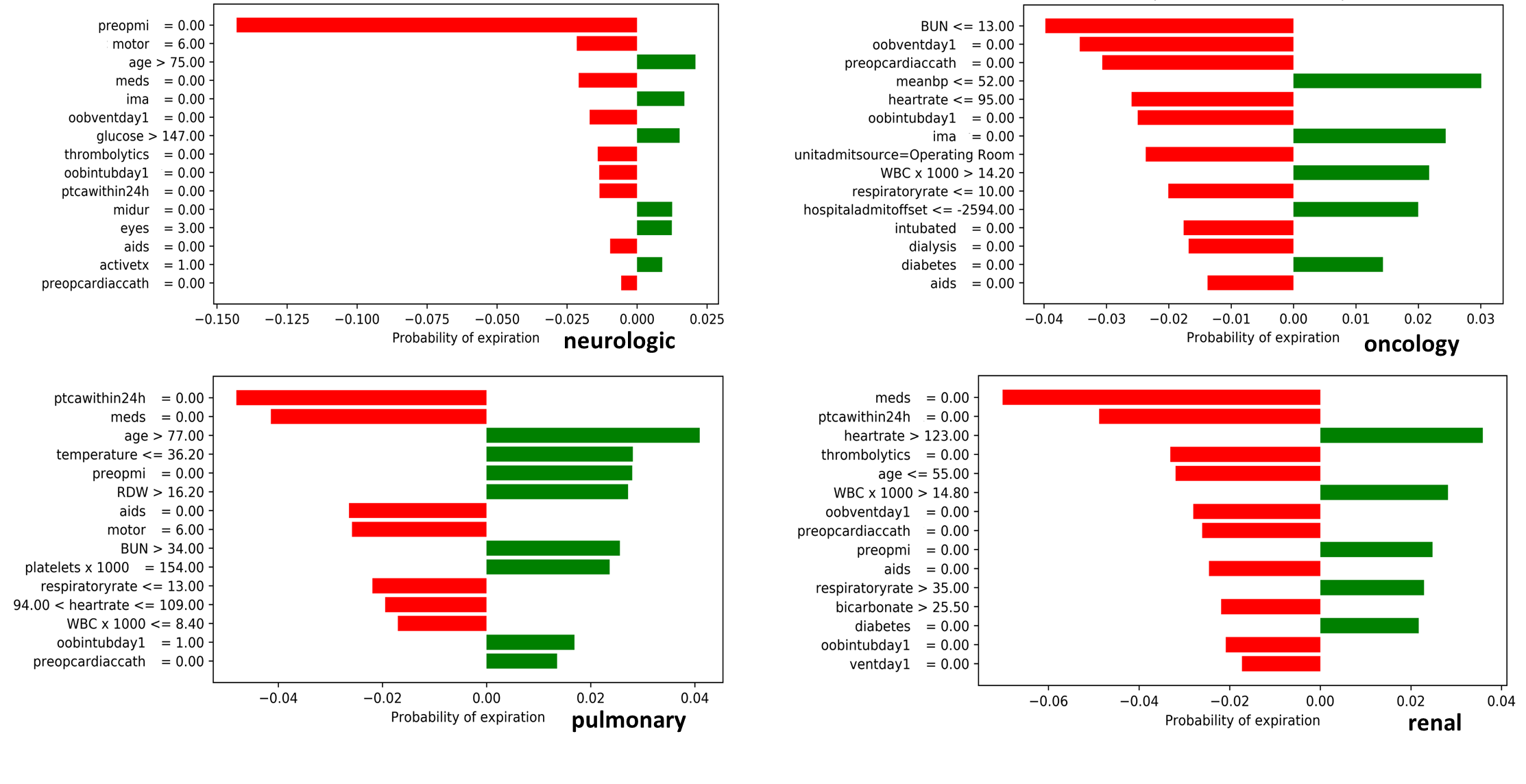

Supplement: S11 Fig — (TIF) [file pone.0262895.s038.tif]

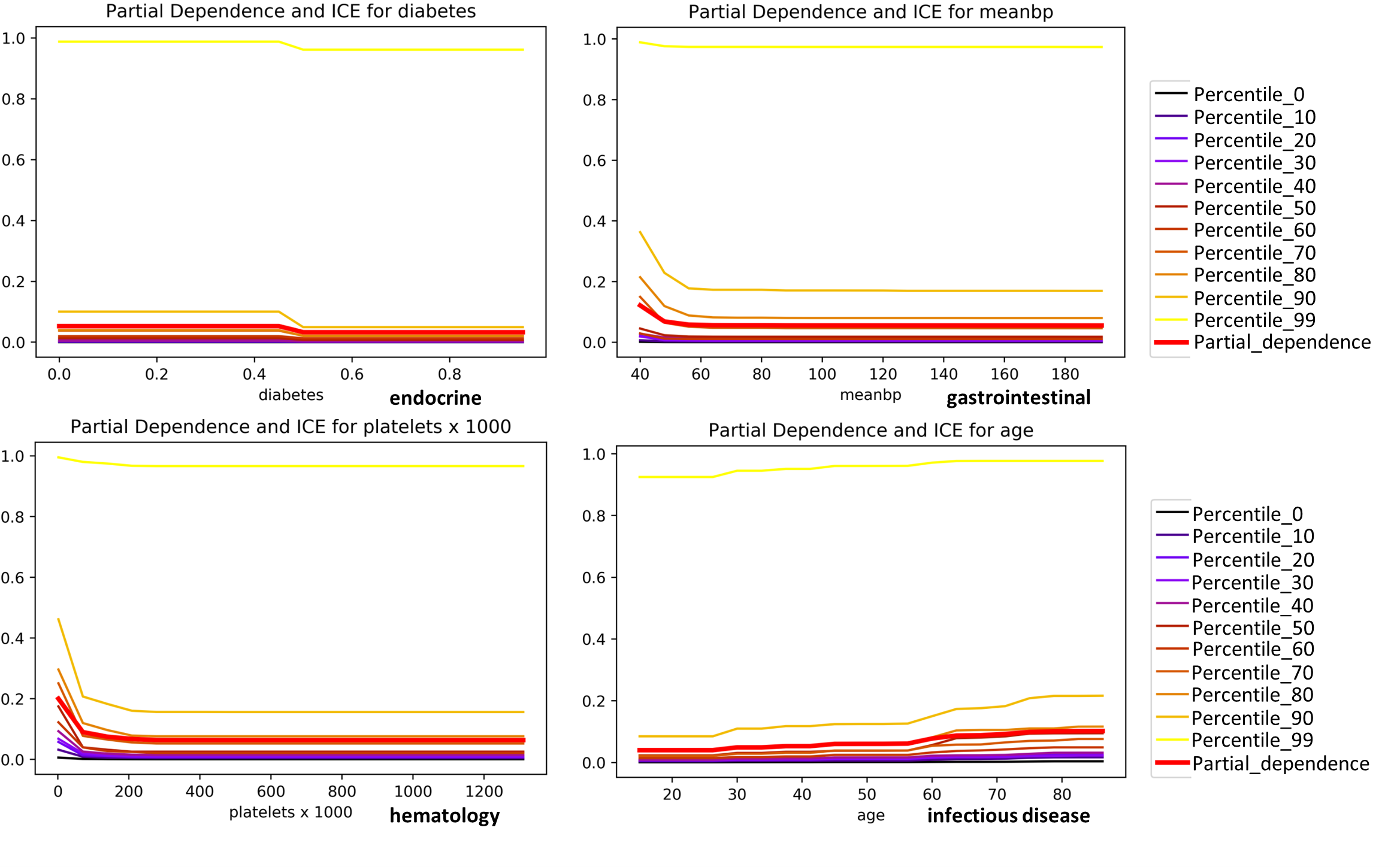

Supplement: S12 Fig — (TIF) [file pone.0262895.s039.tif]

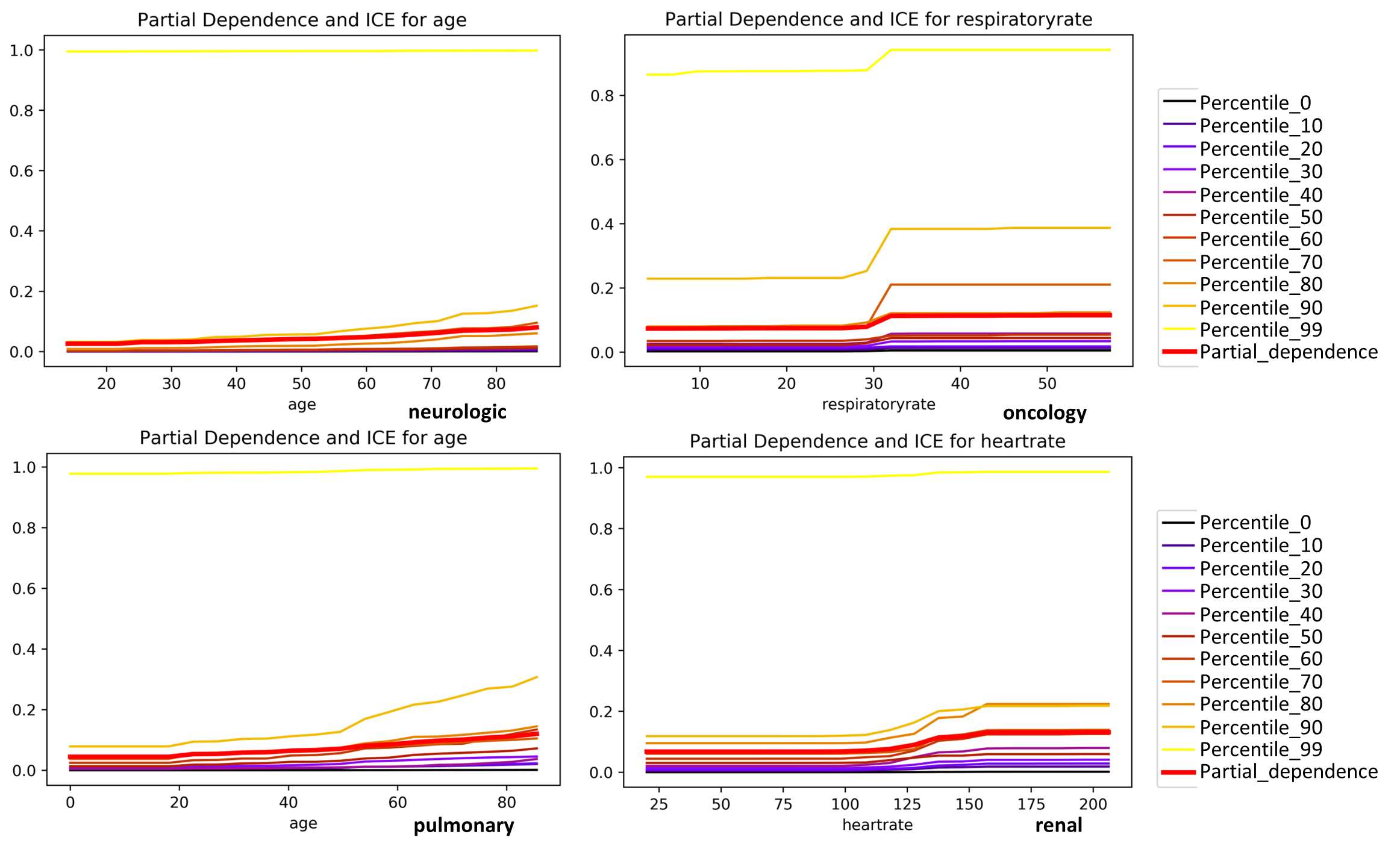

Supplement: S13 Fig — (TIF) [file pone.0262895.s040.tif]

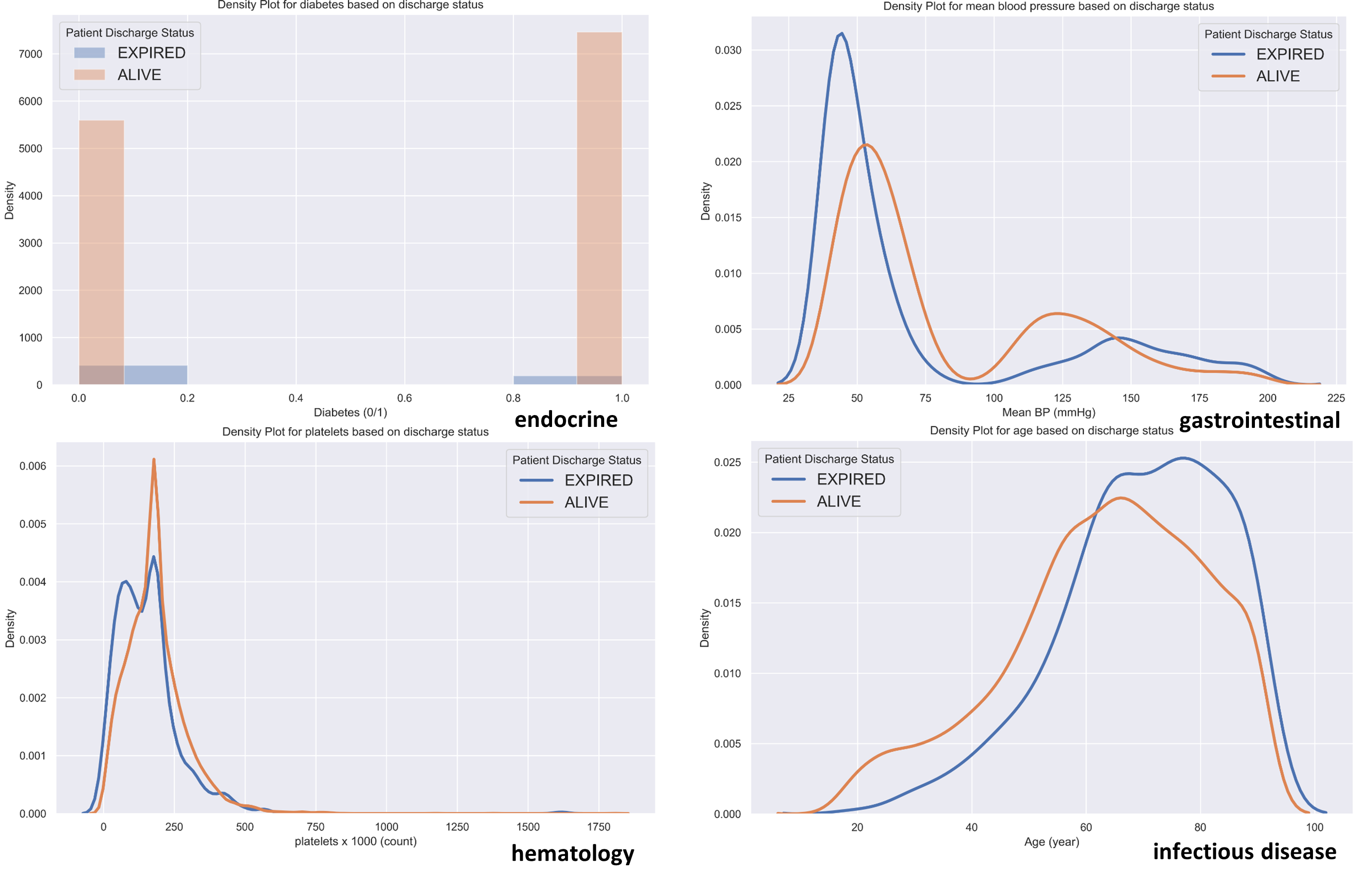

Supplement: S14 Fig — (TIF) [file pone.0262895.s041.tif]

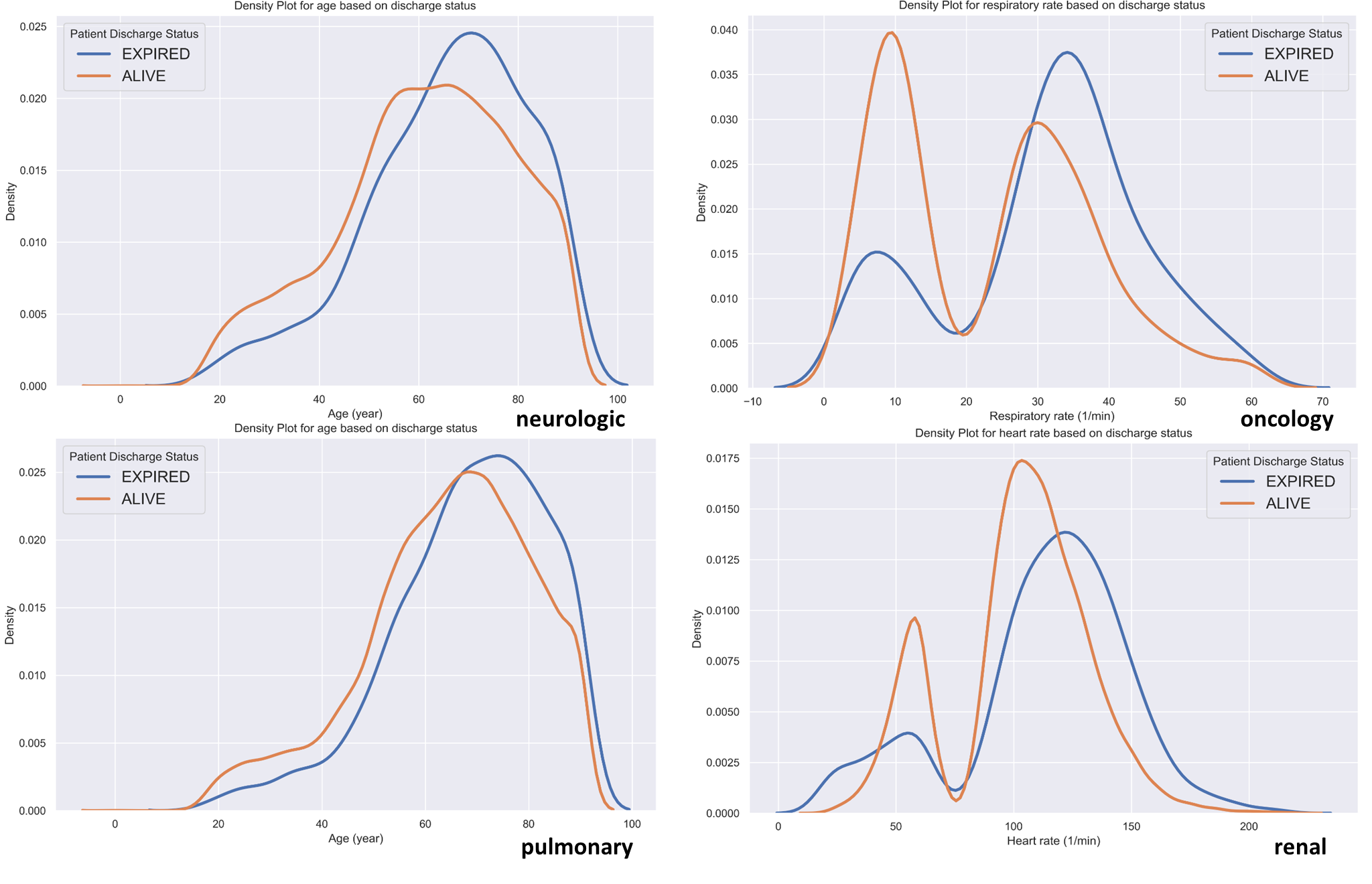

Supplement: S15 Fig — (TIF) [file pone.0262895.s042.tif]
